# A Novel Retrotransposon Inserted in the Dominant *Vrn-B1* Allele Confers Spring Growth Habit in Tetraploid Wheat (*Triticum turgidum* L.)

**DOI:** 10.1534/g3.111.001131

**Published:** 2011-12-01

**Authors:** C.-G. Chu, C. T. Tan, G.-T Yu, S. Zhong, S. S. Xu, L. Yan

**Affiliations:** * Department of Plant Pathology and; ‡ Department of Entomology, North Dakota State University, Fargo, North Dakota 58108; † Department of Plant and Soil Sciences, Oklahoma State University, Stillwater, Oklahoma 74078; § United States Department of Agriculture, Agricultural Research Service, Northern Crop Science Laboratory, Fargo, North Dakota 58102

**Keywords:** *Triticum turgidum*, tetraploid wheat, vernalization, *Vrn-B1*, retrotransposon

## Abstract

Vernalization genes determine winter/spring growth habit in temperate cereals and play important roles in plant development and environmental adaptation. In wheat (*Triticum* L. sp.), it was previously shown that allelic variation in the vernalization gene *VRN1* was due to deletions or insertions either in the promoter or in the first intron. Here, we report a novel *Vrn-B1* allele that has a retrotransposon in its promoter conferring spring growth habit. The *VRN-B1* gene was mapped in a doubled haploid population that segregated for winter-spring growth habit but was derived from two spring tetraploid wheat genotypes, the durum wheat (*T. turgidum* subsp. *durum*) variety ‘Lebsock’ and *T. turgidum* subsp. *carthlicum* accession PI 94749. Genetic analysis revealed that Lebsock carried the dominant *Vrn-A1* and recessive *vrn-B1* alleles, whereas PI 94749 had the recessive *vrn-A1* and dominant *Vrn-B1* alleles. The *Vrn-A1* allele in Lebsock was the same as the *Vrn-A1c* allele previously reported in hexaploid wheat. No differences existed between the *vrn-B1* and *Vrn-B1* alleles, except that a 5463-bp insertion was detected in the 5′-UTR region of the *Vrn-B1* allele. This insertion was a novel retrotransposon (designated as retrotrans_VRN), which was flanked by a 5-bp target site duplication and contained primer binding site and polypurine tract motifs, a 325-bp long terminal repeat, and an open reading frame encoding 1231 amino acids. The insertion of retrotrans_VRN resulted in expression of *Vrn-B1* without vernalization. Retrotrans_VRN is prevalent among *T. turgidum* subsp. *carthlicum* accessions, less prevalent among *T. turgidum* subsp. *dicoccum* accessions, and rarely found in other tetraploid wheat subspecies.

Vernalization genes determine winter/spring growth habit in temperate cereals and are one of the major genetic factors that affect plant life cycle and enable the crop plant to adapt to a wide range of environments (reviewed by Distelfeld *et al.* 2009; Rousset *et al.* 2011; Golovnina *et al.* 2010). On the basis of vernalization requirement, wheat (*Triticum* L. sp.) crops are traditionally divided into winter and spring wheat. Winter wheat requires an exposure to a period of low temperatures to induce flowering, whereas spring wheat precludes the requirement for low temperatures to flower (Stelmakh 1987). Increasing our understanding of allelic variation in vernalization genes will be useful for more effective development of wheat cultivars adapted to various environments.

In diploid wheat (*T. monococcum* L., 2*n* = 2*x* = 14, A^m^A^m^) and barley (*Hordeum vulgare* L., 2*n* = 2*x* = 14, HH), vernalization is controlled by at least three genes, *VRN1*, *VRN2*, and *VRN3* (Takahashi and Yasuda 1971; Tranquilli and Dubcovsky 2000; Yan *et al.* 2004b, 2006; Hemming *et al.* 2009). In hexaploid wheat (*T. aestivum* L, 2*n* = 6*x* = 42, AABBDD), the three homoeologues of the *VRN1* gene, *VRN-A1*, *VRN-B1*, and *VRN-D1* on the long arms of chromosomes 5A, 5B, and 5D, respectively, are major genes determining growth habit (Pugsley 1971; Law *et al.* 1976; Worland 1996; Dubcovsky *et al.* 1998; Snape *et al.* 2001; Barrett *et al.* 2002; Danyluk *et al.* 2003; Leonova *et al.* 2003; Trevaskis *et al.* 2003; Yan *et al.* 2003, 2004a; Fu *et al.* 2005). A dominant allele for any one of the three genes leads to spring growth habit regardless of the allelic state of the other vernalization genes, but the presence of recessive alleles for all three genes leads to winter growth habit (Tranquilli and Dubcovsky 2000; Yan *et al.* 2006; Zhang *et al.* 2008). *VRN1* is an ortholog of the *Arabidopsis* meristem identity gene *AP1* (Danyluk *et al.* 2003; Murai *et al.* 2003; Trevaskis *et al.* 2003; Yan *et al.* 2003), which encodes a MADS-box protein and is responsible for the initiation of the transition from vegetative to reproductive apices in *Arabidopsis* (Mandel *et al.* 1992).

As one of a few genes that have been successfully cloned in wheat, *VRN1* and its allelic variants have been extensively studied. The dominant *Vrn-A1* allele for spring growth habit originated from mutations either in the promoter or in the first intron of a recessive *vrn-A1* allele for wild-type winter growth habit in diploid, tetraploid (*T. turgidum* L., 2*n* = 4*x* = 28, AABB), and hexaploid wheat (Yan *et al.* 2004a; Fu *et al.* 2005; Dubcovsky *et al.* 2006; Pidal *et al.* 2009). In the promoter region, different lengths of deletions (*Vrn-A^m^1a*, *Vrn-A^m^1b*, *Vrn-A^m^1g*) and a 1-bp deletion (*Vrn-A^m^1f*) in the CArG-box were identified in *Vrn-A^m^1* of *T. monococcum* (Yan *et al.* 2003; Dubcovsky *et al.* 2006; Pidal *et al.* 2009). In addition to similar deletions (*Vrn-A1d* and *Vrn-A1e*) in the CArG-box region, a deletion in the VRN-box (*Vrn-A1b*) was found in tetraploid wheat (Yan *et al.* 2004a; Pidal *et al.* 2009). In hexaploid wheat, most spring cultivars carry a dominant *Vrn-A1a* allele that has a duplicated MITE insertion in the VRN-box (Yan *et al.* 2004a). On the contrary, the dominant *Vrn-B1* and *Vrn-D1* alleles do not harbor variation in their promoter regions, but they contain large deletions in the first intron in tetraploid wheat and hexaploid wheat (Yan *et al.* 2004a; Fu *et al.* 2005).

In this study, we discovered a novel allele of the dominant *Vrn-B1* gene in tetraploid wheat as a result of mapping genetic loci associated with growth habit in a doubled haploid (DH) population derived from two spring tetraploid wheat genotypes, a cultivar ‘Lebsock’ of durum wheat [*T. turgidum* subsp. *durum* (Desf.) Husnot, 2*n* = 4*x* = 28, AABB] and an accession (PI 94749) of *T. turgidum* subsp. *carthlicum* (Nevski in Kom.) Á. Löve & D. Löve (2*n* = 4*x* = 28, AABB). Variation between the *Vrn-B1* and *vrn-B1* alleles that segregate in this population was exclusive to a retrotransposon insertion in the 5′-untranslated region (UTR) of the *Vrn-B1* allele in PI 94749. Molecular markers were developed to determine the frequencies of the dominant *Vrn-B1* and recessive *vrn-B1* alleles in a global collection of tetraploid wheat. The evolutionary mechanism that induced the allelic variation and resulted in a conversion of winter to spring growth habit in tetraploid wheat is discussed.

## Materials and Methods

### Plant materials

A DH population (hereafter referred as to LP749), consisting of 146 lines derived from a cross between Lebsock and PI 94749 (Chu *et al.* 2010), was used to identify genes responsible for growth habit within the population (supporting information, Table S1). The F_1_ and F_2_ plants from the cross between Lebsock and PI 94749 and two backcross populations (Lebsock/PI 94749//Lebsock and Lebsock/2*PI 94749) were then generated to evaluate the genetic effects of each identified gene in the DH population.

### Evaluation of winter/spring growth habit

Growth habit was evaluated following the procedures described in Yan *et al.* (2003). Plants of LP749, two parental lines, F_1_ hybrids, and F_2_ and backcross populations were grown in a greenhouse at 20–25° without vernalization and under a 16 h photoperiod by providing additional artificial light. Under this temperature-photoperiod–controlled regime, plants that flowered within two months after planting were classified as spring types, and plants that were still in vegetative growth one month after the spring plants flowered were characterized as winter types. The winter-type plants were then vernalized to induce flowering and seed production. More than 20 plants for each of the two parental lines and their F_1_ hybrids were tested in three greenhouse seasons. Evaluation included 200 plants for the F_2_ population and 100 plants for each of the two BC_1_F_2_ populations. For the DH lines, growth habit was evaluated in two greenhouse seasons.

### QTL analysis

The linkage maps previously developed for the LP749 population (Chu *et al.* 2010) were used in this study. For QTL analysis, a subset of 188 markers that were spaced greater than 2 cM apart and gave the most complete genome coverage was used, and values of 0 and 1 were assigned to spring and winter growth habits, respectively. Composite interval-regression mapping (CIM) was performed using the computer program Map Manager QTX (Manly *et al.* 2001) to evaluate marker intervals associated with growth habit. A permutation test with 5000 permutations indicated that a logarithm of the odds (LOD) threshold of 2.91 in this population yielded an experiment-wise significance level of 0.05. Markers with significant (*P* < 0.001) main effects were tested against all other markers (Manly *et al.* 2001) in the dataset to identify significant (*P* < 10^−6^) interactions among QTL.

### Gene-specific marker development and sequence analysis

Allelic variation in the *VRN-A1* and *VRN-B1* genes is mostly confined to the promoter regions and the first introns of the genes (Fu *et al.* 2005; Yan *et al.* 2004a; Pidal *et al.* 2009), and 10 pairs of primers that were designed from those regions were used in this study (Table 1). In addition, several primers were designed to amplify the *VRN-A1* and *VRN-B1* genes based on the conserved sequence between *Vrn-A^m^1* (BAC 231A16 of *T. monococcum* accession DV92, GenBank ID AY188331) (Yan *et al.* 2003) or *VRN-A1* (BAC 1226C17, GenBank ID AY616452) and *VRN-B1* (BAC 1225D16, GenBank ID AY616453) of durum wheat ‘Langdon’ (Yan *et al.* 2004a). In addition to these, several primers specific to *VRN-B1* were designed to identify allelic variation in *VRN-B1* (Table 1).

**Table 1:** Primers used for detecting allelic variation in *VRN-A1* and *VRN-B1* in tetraploid wheat

Primer	Sequence (5′–3′)	Position		Reference
		Loci	Gene Region	
VRN1AF	GAAAGGAAAAATTCTGCTCG	*VRN-A1*	Promoter	Yan *et al.* (2004a)
VRN1AR	TGCACCTTCCC(C/G)CGCCCCAT	*VRN-A1*	Promoter	
Ex1/C/F	GTTCTCCACCGAGTCATGGT	*VRN-A1*	Intron 1	Fu *et al.* (2005)
Intr1/A/R3	AAGTAAGACAACACGAATGTGAGA	*VRN-A1*	Intron 1	
Intr1/C/F	GCACTCCTAACCCACTAACC	*VRN-A1*	Intron 1	
Intr1/AB/R	TCATCCATCATCAAGGCAAA	*VRN-A1*	Intron 1	
Intr1/B/F	CAAGTGGAACGGTTAGGACA	*VRN-B1*	Intron 1	
Intr1/B/R3	CTCATGCCAAAAATTGAAGATGA	*VRN-B1*	Intron 1	
Intr1/B/F	CAAGTGGAACGGTTAGGACA	*VRN-B1*	Intron 1	
Intr1/B/R4	CAAATGAAAAGGAATGAGAGCA	*VRN-B1*	Intron 1	
VRNBPF1	CCCCTGCTACCAGTGCCTACTA	*VRN-B1*	960 bp upstream from start codon	Newly developed
VRNBPF2	GGCTTGGGGTGTAGGGTTGG	*VRN-B1*	18 bp upstream from start codon	
VRNBPF3	CCCTCTCTTCCGCCTCACCCAAC	*VRN-B1*	116 bp upstream from start codon	
VRNBPR1	GCCCCATCTCCGCTGGAGAACG	*VRN-B1*	Contained start codon	
VRNBPR2	CAGGTGGTTGGGTGAGGCGGAAG	*VRN-B1*	110 bp upstream from start codon	
VRNBPR3	CGTAAATATCCCCAGCCAGA	*VRN-B1*	586 bp downstream from start codon	
VBINSR1	CCCGCTTGTTGGCTGGTGAG	*VRN-B1*	42 bp downstream from VRNBPF3 in PI 94749	

Total genomic DNA was isolated from leaves (Faris *et al.* 2000), and the DNA concentration was adjusted to 50–100 ng/µl for PCR reactions. For DNA fragments of less than 2 kb, a total volume of 15 µl per PCR reaction was conducted, which contained 200 nM of each primer, 0.2 mM of each dNTP, 1.5 mM MgCl_2_, 1 unit *Taq* polymerase (Qiagen Sciences Inc., Germantown, MD), and 100–200 ng of template DNA. The amplification was performed at 94° for 4 min, followed by 40 cycles, each consisting of 30 sec at 94°, 45 sec at 50–60° (depending on the annealing temperatures of primers), 45–90 sec at 72° (depending on the size of the products with a rate of 1 min per kb), and a final extension step of 10 min at 72°. For PCR products greater than 2 kb, a total volume of 25 µl per reaction was performed using the LongAmp *Taq* polymerase kit (New England Biolabs Inc., Ipswich, MA), and 200–400 ng of template DNA was applied in each reaction. PCR amplification was performed at 94° for 30 sec, followed by 30 cycles, each consisting of 20 sec at 94°, 20 sec at 55–58°, 5–10 min at 65° (time estimated based on the rate of 50 sec/kb), and a final extension step of 10 min at 65°. PCR products were mixed with 3 µl loading buffer (40% sucrose, 0.2% bromophenol blue) for electrophoresis, which was carried out on 0.8–1% agarose gels in 1× TAE buffer at 80 W for 1–1.5 h. Gels were stained using 0.001% GelRed (Biotium Inc., Hayward, CA) for 10 min and visualized using a Gel Logic 200 imaging system (Kodak Inc., New Haven, CT). PCR products of the *VRN-B1* gene were purified with the Qiagen spin miniprep kit (Qiagen Sciences Inc.), cloned into the pCR2.1 TOPO vector (Invitrogen Inc., Carlsbad, CA), and then sequenced by the DNA Facility at Iowa State University (Ames, Iowa).

PCR markers for *VRN-A1* and *VRN-B1* were developed to analyze the LP749 population. The new markers were evaluated for linkage with the previously mapped SSR markers (Chu *et al.* 2010) using the computer program MAPMAKER (V2.0) for Macintosh (Lander *et al.* 1987) with a minimum LOD threshold of 3.0 and the Kosambi mapping function (Kosambi 1944).

### Frequency of the *Vrn-B1* allele carrying the retrotransposon insertion in tetraploid wheat

The newly developed *VRN-B1* markers were used to investigate the frequency of the dominant and recessive alleles in 154 spring accessions/lines of six domesticated tetraploid wheat subspecies, including durum wheat, *T. turgidum* subsp. *carthlicum*, *T. turgidum* subsp. *dicoccum* (Schrank ex Schübler) Thell., *T. turgidum* subsp. *polonicum* (L.) Thell., *T. turgidum* subsp. *turanicum* (Jakubz.) Á. Löve & D. Löve, and *T. turgidum* subsp. *turgidum* (Table S2). For accessions that carried the retrotransposon insertion as indicated by the *Vrn-B1* marker, the 5′ and 3′ ends of the insertion were sequenced. The 5′ end of the insertion was amplified using primers VBINS2F (5′-CTCACCCAACCACCTGACAGC-3′) and VBINS2R (5′-ATCCGCCCCACTTGGGATT-3′), and the 3′ end of the insertion was amplified using primers VBINS5F (5′-CAACCAGAGGCAATTCTGGACAC-3′) and VRNBPR1 (5′-GCCCCATCTCCGCTGGAGAACG-3′).

### Sequence analysis of the retrotransposon insertion in *Vrn-B1*

The web-based computer program GenScan at GeniusNet (http://genome.dkfz-heidelberg.de/cgi-bin/GENSCAN/genscan.cgi) was used to predict the open reading frame and deduce the encoded amino acid sequences. The primer binding site (PBS) was identified by aligning the sequence downstream of the 5′ long terminal repeat (LTR) to that of 3′ regions of tRNAs. The polypurine tract (PPT) was identified by examining the purine-rich region upstream of the 3′ LTR. The web-based computer program RPS-BLAST (reverse position-specific BLAST) (http://www.ncbi.nlm.nih.gov/Structure/cdd/docs/cdd_search.html) was used to identify conserved domains in the deduced amino acid sequences.

### Cloning and sequencing of the *Vrn-B1* and *vrn-B1* alleles

To determine whether the insertion in the PI 94749 *Vrn-B1* was the only difference compared with the Lebsock *vrn-B1* allele, the complete gene, including the 27-bp UTL upstream from the start codon, the 44-bp UTL downstream from the stop codon, and 12,343 bp between the start codon and the stop codon, was cloned for sequence analysis. For convenience of cloning, this large gene was divided into three fragments. The first fragment accounted for the 5′-UTR region of 27 bp, exon 1, and 4778 bp of intron 1, and was amplified by primers VRN-A1F1 (5′-TAGGGTTGGCCCGGTTCTC-3′) and VRNB1-C1-R7 (5′-AGGCCGACGCCTTGTGAC-3′). The second fragment, which spanned most of intron 1, consisted of 4927 bp that overlapped 917 bp with the first fragment and was amplified by primers VRNB1-C1-F6 (5′-ACCGCACTCCTAACCCACTA-3′) and VRNB1-C2-R2 (5′-ATGCCTAGATAAGTAGGACGATACGA-3′). The third fragment spanned 3069 bp of intron 1, which overlapped 2192 bp with the second fragment, and the remaining region, including 7 exons and 6 introns. The third fragment was amplified by primers VRNB1-INT1-F4 (5′-TCCCTAAAGAAAGATCGGAAGAC-3′) and VRNB1-3end-R3 (5′-GTTACTCTCTACTGAATAGTACGCTTA-3′). These primers specific to *VRN-B1* were designed by comparison of *VRN-A1* (AY616452) and *VRN-B1* (AY616453) of Langdon (Yan *et al.* 2004a), and the sizes of PCR products were estimated based on the sequence of the *VRN-B1* gene.

### Gene expression analysis of *VRN-A1* and *VRN-B1*

For gene expression experiments, the two parents along with 16 spring DH lines carrying either *Vrn-A1* or *Vrn-B1* or both and 8 winter DH lines carrying recessive *vrn-A1* and *vrn-B1* alleles were grown in the greenhouse under constant temperature (20–25°) and long day length (16 h light). For each of the winter DH lines, five plants were vernalized by treating them for 6 weeks at 4–6° and 16 h day length, and another five plants were not vernalized and kept in the greenhouse as controls.

For detecting transcripts of *VRN-A1* and *VRN-B1*, leaf samples from all tested lines were collected at 9:00 am to avoid potential effect of the *VRN-1* diurnal expression pattern reported by Shimada *et al.* (2009). Total RNA was extracted using the TRIZOL method (Invitrogen Inc.) (Yan *et al.* 2003) from leaves at the third leaf stage, and the two conserved primers, Ex4-5_F1 and Ex8_R1 specific for *VRN-A1* and *VRN-B1*, respectively, were used to simultaneously amplify their transcripts using the protocol described previously (Loukoianov *et al.* 2005). The mixed *VRN-A1* and *VRN-B1* transcripts were separated and distinguished by digesting the PCR products with restriction enzyme *Hin*fI, which resulted in undigested PCR products for *VRN-A1* (313 bp) and digested PCR products for *VRN-B1* (173 bp + 110 bp + 30 bp) (Loukoianov *et al.* 2005). Because no transcripts were observed in the unvernalized winter DH lines at the third leaf stage, RNA samples were also collected at the seventh and ninth leaf stages for these lines. The *VRN-A1* and *VRN-B1* transcripts were further investigated in the winter DH lines that were vernalized for six weeks. In addition, the primer pair specific for actin (ACTIN, forward: TGTGGATATCAGGAAGGA and reverse: CTCATACGGTCAGCAATAC) (Yan *et al.* 2003), which produced a cDNA fragment of 85 bp, was used as an endogenous control for detecting transcripts.

## Results

### Restoration of winter growth habit in the DH population derived from two spring parents

**Figure 1:**
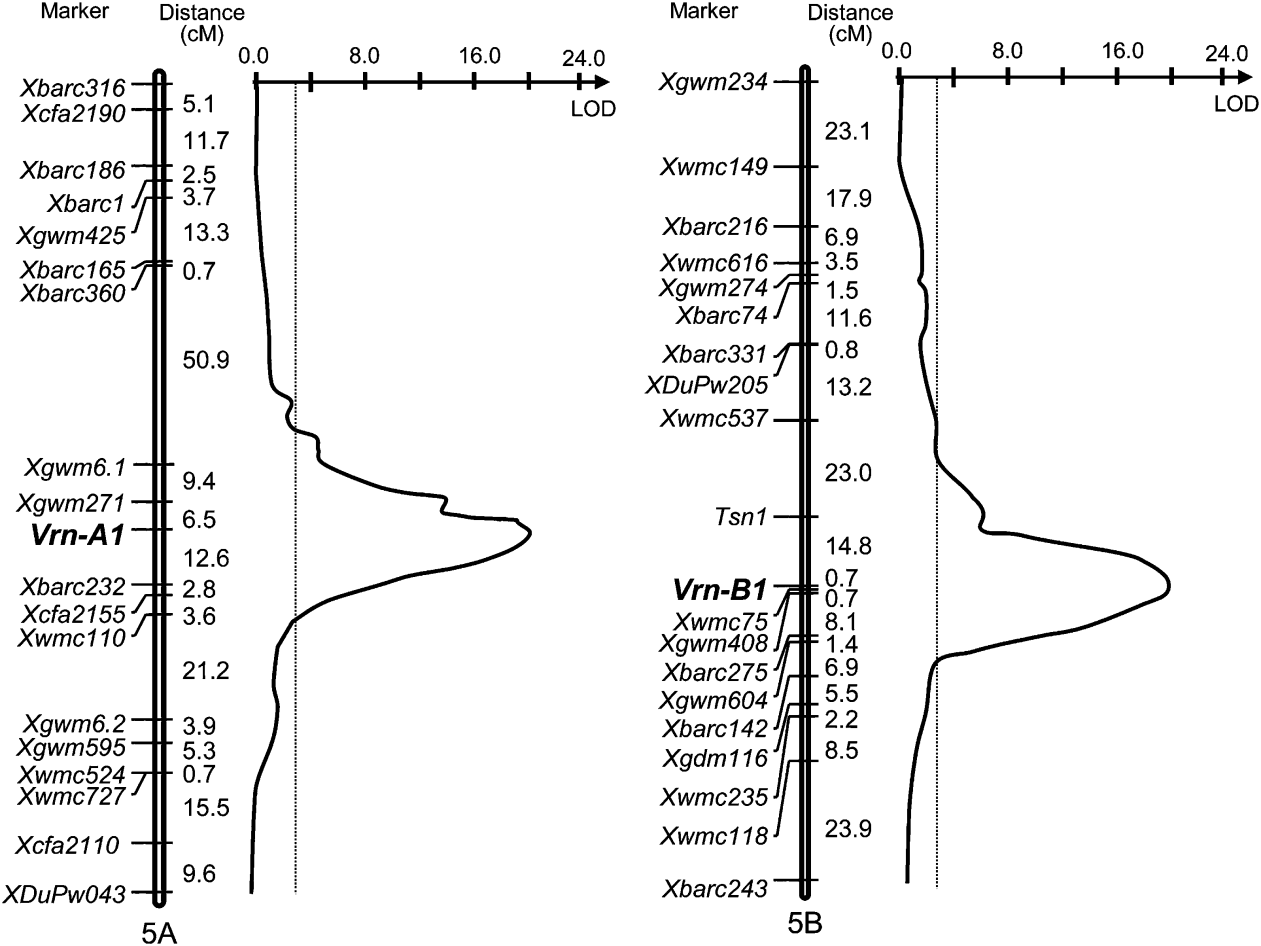
QTL mapping for growth habit segregation in the Lebsock × PI 94749–derived DH population. QTL analysis was performed with composite interval mapping using the computer program Map Manager QTX (Manly *et al.* 2001). The positions of marker loci are shown to the left of each linkage group and centiMorgan (cM) distances between loci are shown along the right. The vertical dotted line indicates the logarithm of the odds (LOD) significance threshold of 2.91. *R^2^* and LOD values are 0.30 and 19.67 for the QTL on chromosome 5AL (left), and 0.30 and 19.45 for the QTL on chromosome 5BL (right), respectively.

Of 146 DH lines generated from two spring parents, 101 were spring type and 45 were winter type (Table 2 and Table S1), indicating winter growth habit was restored due to homoeoallelic recombination of genes controlling growth habit. The observed segregation ratio was not significantly different from a 3:1 ratio (χ^2^_df = 1_ = 2.64, *P* = 0.10) and fit a two-gene model. CIM revealed that two QTL were significantly associated with growth habit and centered at the *VRN-A1* and *VRN-B1* loci on chromosome arms 5AL and 5BL, respectively (Figure 1). The *VRN-A1* and *VRN-B1* QTL had LOD values of 19.67 and 19.45, respectively, and they each explained 30% of the total phenotypic variation. No other major loci for growth habit were detected in this population.

**Table 2:** Characterization of winter/spring growth habit

Lines/Plants	Number of Plants/Lines		
	Total	Winter Type	Spring Type
Parent			
Lebsock	> 20	0	> 20
PI 94749	> 20	0	> 20
F_1_	> 20	0	> 20
F_2_	200	13	187
DH population	146	45	101
Backcross population			
Lebsock/PI 94749//Lebsock	100	0	100
Lebsock/2^*^PI 94749	100	0	100

Winter/spring growth habit of parental lines Lebsock and PI 94749 and their F_1_ plants, F_2_ plants, doubled haploid (DH), and two BC_1_F_2_ populations.

### Analysis of genetic effects of *VRN-A1* and *VRN-B1* on winter/spring growth habit in F_1_ plants and in F_2_ and BC_1_F_2_ populations

The parental lines and their F_1_ hybrids were all spring type (Table 2), indicating that spring type was dominant and that the two parents each carried only one, but different, dominant allele of either *Vrn-A1* or *Vrn-B1*. Among 200 F_2_ plants, 187 were spring type and 13 were winter type (Table 2), and the segregation fit a 15:1 ratio (χ^2^_df = 1_ = 0.02, *P* = 0.88). In the two BC_1_F_2_ populations, each consisting of 100 plants, no winter-type plants were observed (Table 2). Therefore, the results of the QTL analysis in the LP749 population combined with the results from the F_1_ plants and the F_2_ and BC_1_F_2_ populations demonstrated that the genotypes of Lebsock and PI 94749 were *Vrn-A1Vrn-A1vrn-B1vrn-B1* and *vrn-A1vrn-A1Vrn-B1Vrn-B1*, respectively.

### Allelic variation in *VRN-A1* between Lebsock and PI 94749

**Figure 2:**
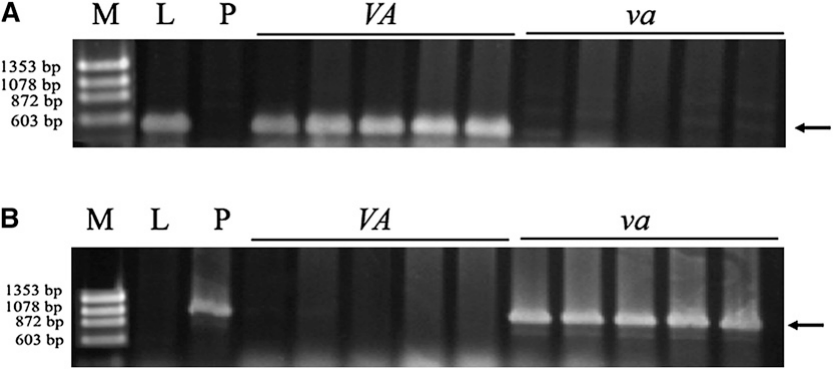
Markers for allelic variation in the first intron of *VRN-A1*. PCR products were amplified by primer pairs Ex1/C/F and Intr1/A/R3 (A) and Intr1/C/F and Intr1/AB/R (B) for detecting presence and absence of the large deletion in the first intron of *VRN-A1*, respectively. Lanes: M = size standard; L = Lebsock; *P* = PI 94749; *VA* and *va* are DH lines carrying dominant and recessive *VRN-A1*, respectively. Arrows indicate the expected size of the products.

The paired primers VRN1AF and VRN1AR (Table 1) were used to detect allelic variation in the promoter region of *VRN-A1* between Lebsock and PI 94749. A 484-bp fragment was amplified in both parental lines. Sequencing results confirmed that they had the same DNA sequences as the recessive *vrn-A1* allele (Figure S1). However, the paired primers Ex1/C/F and Intrl/A/R3 (Table 1) produced an expected 522-bp fragment in Lebsock (Figure 2A), which demonstrated the presence of the large deletion within the first intron of the *Vrn-A1* allele, whereas primer pair Intrl/C/F and Intrl/AB/R (Table 1) generated the expected 1068-bp fragment in PI 94749 (Figure 2B), which demonstrated the absence of this large deletion in the *vrn-A1* allele. Both primer pairs were then used to genotype all DH lines in LP749, and *VRN-A1* mapped to a location that defined the peak of the QTL on chromosome 5AL as expected (Figure 1).

### Allelic variation in *VRN-B1* between Lebsock and PI 94749

The two primer pairs Intr1/B/F and Intr1/B/R3 and Intr1/B/F and Intr1/B/R4 (Table 1), which had been used to detect the presence/absence of a large deletion in intron 1 of the *VRN-B1* allele (Fu *et al.* 2005), were used to analyze Lebsock and PI 94749, but no polymorphic products were observed. Sequencing results confirmed that both Lebsock and PI 94749 had the same 1149-bp fragment (Figure S2) of the *vrn-B1* allele as in Langdon (GenBank ID AY747602) (Fu *et al.* 2005). Therefore, no allelic variation within intron 1 was found between the *Vrn-B1* allele in PI 94749 and the *vrn-B1* allele in Lebsock.

**Figure 3:**
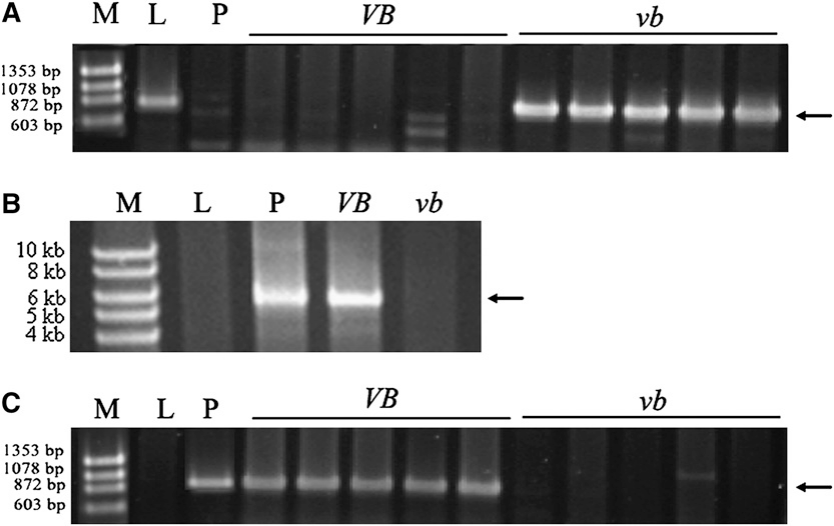
Markers for allelic variation in *VRN-B1*. PCR products were amplified by primer pairs VRNBPF1 and VRNBPR1 (A), VRNBPF3 and VRNBPR1 (B), and VRNBPF1 and VBINSR1 (C). Lanes: M = size standard; L = Lebsock; *P* = PI 94749; *VB* and *vb* are DH lines carrying dominant and recessive *VRN-B1*, respectively. Arrows indicate the expected polymorphic PCR products.

To determine whether allelic variation existed between the promoter regions of *Vrn-B1* and *vrn-B1*, PCR analysis was conducted using the primers pair VRNBPF1 and VRNBPR1 (Table 1). However, only Lebsock yielded an amplified product, which was 989 bp in size (Figure 3A). Sequencing results indicated that the fragment from Lebsock had the same sequence as the reported *VRN-B1* in Langdon (GenBank ID AY616453) (Yan *et al.* 2004a) (Figure S3). This primer pair was then used to genotype all DH lines, and the 989-bp fragment was amplified only in the lines carrying the *vrn-B1* allele (Figure 3A), which facilitated mapping of the *VRN-B1* gene in the DH population (Figure 1).

To understand why PI 94749 did not yield a product when amplified with VRNBPF1/VRNBPR1, a new primer (VRNBPR2) (Table 1) located 95 bp upstream of VRNBPR1, was combined with the primer VRNBPF1 for PCR. This primer pair produced the same expected 872-bp fragment in both Lebsock and PI 94749 (Figure S4), indicating that no differences existed in the region between VRNBPF1 and VRNBPR2 of the *VRN-B1* gene. PCR results using the primer pair VRNBPF2 and VRNBPR3 (Table 1), which are located 3 bp upstream and 582 bp downstream of VRNBPR1, respectively, also indicated that Lebsock and PI 94749 had the same expected 647-bp amplicon. Primers VRNBPR2 and VRNBPF2 were 72 bp apart in *vrn-B1* in Lebsock, but they failed to amplify the expected PCR product in PI 94749. We hypothesized that a large insertion might be located between VRNBPF1 and VRNBPR2 in the *Vrn-B1* allele in PI 94749. To test this hypothesis, a long-range PCR reaction, including primers VRNBPF3 (with sequence overlapped with VRNBPR2) and VRNBPR1, was conducted. A 5.5-kb fragment was amplified in PI 94749 and a DH line carrying the dominant *Vrn-B1* allele but not in Lebsock or a DH line carrying the recessive *vrn-B1* allele (Figure 3B).

### Sequence analysis of the 5.5-kb insertion

**Figure 4:**
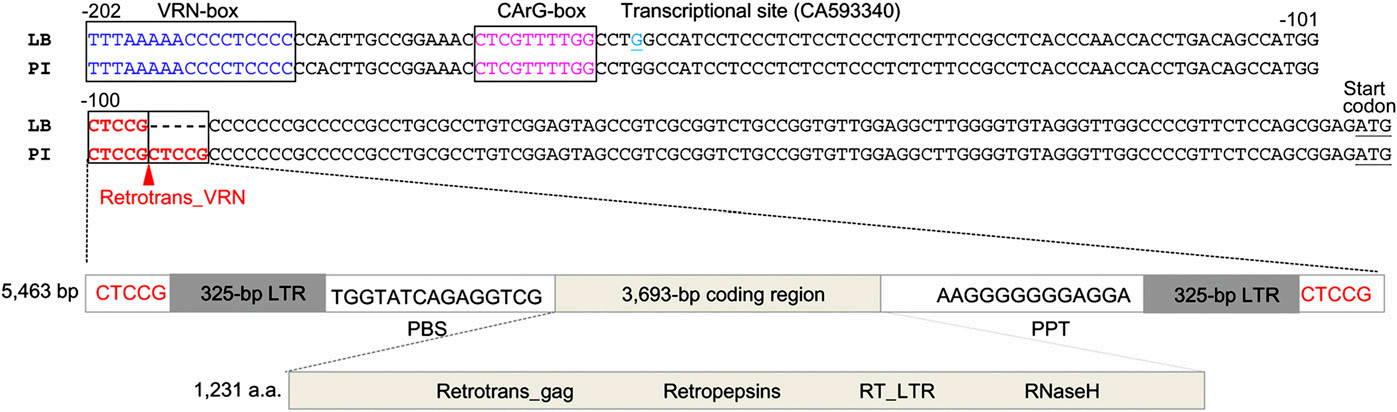
Position and structure of retrotrans_VRN identified in the dominant *Vrn-B1* allele of PI 94749. The aligned sequences were from positions at VRN-box to ATG start codon in PI 94749 (PI) and Lebsock (LB). The VRN-box and CArG-box are framed and shown in blue and pink, respectively. The ATG start codon is underlined. The duplicated target site CTCCG is shown in red with the position of retrotrans_VRN indicated by a red triangle. Positions of the primer binding site (PBS) and polypurine tract (PPT), long terminal repeat (LTR), and the coding region are all indicated. The numbers “−202” and “−101” indicate the start base positions in each line relative to start codon, and “−100” indicates the end base position in each line relative to the start codon. The DNA sequence of the 5.5-kb insertion in the dominant *Vrn-B1* allele in PI 94749 is deposited in the EMBL/GenBank Data Libraries under accession no. HQ186251.

Sequencing of the 5.5-kb fragment from PI 94749 verified that the dominant *Vrn-B1* allele had an insertion of 5463 bp in the 5′-UTR region (Figure 4). PI 94749 had duplicated CTCCG motifs flanking the insertion, whereas Lebsock had only one copy of this motif (Figure 4 and Figure S5). The 5-bp sequence CTCCG is a typical target site duplication (TSD) sequence for a retrotransposable element (retrotransposon) to insert in a new site of the genome (Han *et al.* 2010). A 325-bp LTR was found within the insertion, each having a short inverted repeat 5′-TG...CA-3′ at both ends (Figure S5). Another striking feature of this retrotransposon is the presence of PBS and PPT motifs (Figure 4 and Figure S5).

An open reading frame in the 5463-bp insertion that encoded a total of 1231 amino acids was predicted (Figure S5 and Figure S6). Searches of the deduced amino acid sequence against the NCBI nonredundant protein database (nr) (ftp://ftp.ncbi.nlm.nih.gov/blast/db) showed that it had 63% similarity to a rice retrotransposon (CAD40278) along the whole predicted protein sequence, which included multiple conserved domains, such as retropepsin, retrotrans_gag, and a Gypsy family of RNase H (Figure 4). No other wheat or grass sequences had significant homology to the nucleic acid or deduced amino acid sequences associated with this 5.5-kb insertion. The new retrotransposon in the *Vrn-B1* allele is designated retrotrans_VRN (GenBank ID HQ186251).

To determine whether the retrotrans_VRN insertion was the only difference between *Vrn-B1* in PI 94749 and *vrn-B1* in Lebsock, the complete gene from each parent was sequenced. A total of 13,347 bp, including 960 bp upstream from the start codon, 12,343 bp from the start codon to the stop codon, and 44 bp downstream from the stop codon, was determined for the recessive *vrn-B1* allele (GenBank ID JN817431). The dominant *Vrn-B1* allele (GenBank ID JN817430) was exactly the same as the recessive *vrn-B1* allele in the sequenced region. Therefore, the retrotrans_VRN insertion was the only difference between the PI 94749 *Vrn-B1* allele and the Lebsock *vrn-B1* allele.

### Frequency of the *Vrn-B1* allele containing retrotrans_VRN in tetraploid wheat

The primer VBINSR1 (Table 1), which was close to the 5′ end of the retrotrans_VRN insertion but 42 bp downstream from VRNBPF3, was paired with VRNBPF1 to genotype all DH lines. The expected 928-bp fragment was produced only in PI 94749 and those DH lines carrying the *Vrn-B1* allele (Figure 3C). Therefore, this primer pair was used to observe the presence of retrotrans_VRN in *Vrn-B1*, whereas the primer pair VRNBPF1 and VRNBPR1 was used to detect the absence of retrotrans_VRN in *vrn-B1*.

The above two primer pairs were used to determine the frequency of the *Vrn-B1* allele containing retrotrans_VRN in 154 spring-type accessions/lines from six *T. turgidum* subspecies (Table 2). No accessions/lines from subspecies *durum*, *polonicum*, *turanicum*, or *turgidum* were found to carry retrotrans_VRN. However, 19 of 22 *T*. *turgidum* subsp. *carthlicum* accessions and 3 of 30 *T*. *turgidum* subsp. *dicoccum* accessions had retrotrans_VRN (Table 3 and Table S2). These results indicated that the dominant *Vrn-B1* allele containing retrotrans_VRN is frequent among *T*. *turgidum* subsp. *carthlicum* accessions, is much less frequent among *T*. *turgidum* subsp. *dicoccum* accessions, and is rare among other tetraploid wheat subspecies. The accessions carrying retrotrans_VRN had no specific geographical distribution, suggesting that the insertion of retrotrans_VRN in *Vrn-B1* occurred before the accessions spread worldwide. Sequencing results showed that these 22 accessions carrying retrotrans_VRN had the same sequences as PI 94749 at the 5′ and 3′ LTRs of retrotrans_VRN (Figure S7).

**Table 3:** Presence and absence of the 5.5-kb retrotrans_VRN in 154 spring accessions/lines from six *T. turgidum* subspecies

Subspecies	Number of Accessions/Lines		
	Total	Presence of retrotrans_VRN	Absence of retrotrans_VRN
*T. turgidum* subsp. *durum*	33	0	33
*T. turgidum* subsp. *carthlicum*	22	19	3
*T. turgidum* subsp. *dicoccum*	30	3	27
*T. turgidum* subsp. *polonicum*	20	0	20
*T. turgidum* subsp. *turanicum*	30	0	30
*T. turgidum* subsp. *turgidum*	19	0	19

### Differential expression of *VRN1* genes between the dominant and recessive alleles

Under the same greenhouse conditions without vernalization, 75 plants from 16 spring DH lines carrying the dominant allele(s) of *Vrn-A1*, or *Vrn-B1*, or both, flowered at 43–64 days with an average of 54 days after planting, whereas 32 plants from 8 winter DH lines carrying both *vrn-A1* and *vrn-B1* alleles flowered at 89–145 days with an average of 123 days after planting. Several unvernalized plants did not flower even 145 days after planting when the experiment was terminated (Table S3). On average, vernalization decreased flowering time by 35 days compared with plants of the same lines that did not undergo vernalization (Table S3).

**Figure 5:**
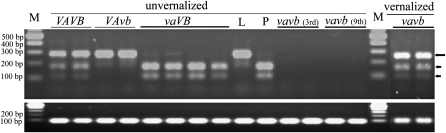
Expression of *VRN-1* in DH lines derived from the cross between Lebsock and PI 94749. (Upper gel) Image of *Hin*fI-digested cDNA fragments amplified by primers Ex4-5_F1 and Ex8_R1 for separating transcripts of *VRN-A1* (313 bp) (indicated by long arrow) and the *VRN-B1* (173 bp + 110 bp + 30 bp) (indicated by short arrows). (Lower gel) Actin was expressed at the same level in each of the lines carrying different *VRN-1* alleles. Lanes: M = size standard; L = Lebsock; *P* = PI 94749; vernalized and unvernalized indicates plants with or without cold treatment at 4–6° for six weeks, respectively. *VAVB*, *VAvb*, *vaVB*, and *vavb* indicate DH lines having genotype of *Vrn-A1Vrn-A1Vrn-B1Vrn-B1*, *Vrn-A1Vrn-A1vrn-B1vrn-B1*, *vrn-A1vrn-A1Vrn-B1Vrn-B1*, and *vrn-A1vrn-A1vrn-B1vrn-B1*, accordingly. The third and ninth mean leaf samples were taken at the third and ninth leaf stages, respectively.

In spring DH lines carrying both dominant *Vrn-A1* and *Vrn-B1* (retrotrans_VRN) alleles, transcripts from both *Vrn-A1* and *Vrn-B1* were observed in leaves at the third leaf stage (Figure 5), whereas in Lebsock and the spring DH lines carrying the dominant *Vrn-A1* and the recessive *vrn-B1* alleles, transcripts were only observed from *Vrn-A1* but not from *vrn-B1* (Figure 5). Similarly, in PI 94749 and the spring DH lines carrying the recessive *vrn-A1* allele and the dominant *Vrn-B1* alleles, transcripts were observed from *Vrn-B1* but not from *vrn-A1* (Figure 5). No transcripts were detected from the recessive allele of either *vrn-A1* or *vrn-B1* in the winter DH lines in leaves at the third, seventh, or ninth leaf stages (Figure 5). However, after the winter lines were vernalized for six weeks, transcripts from both *vrn-A1* and *vrn-B1* were detectable (Figure 5).

## Discussion

In the present study, we analyzed a DH population derived from two tetraploid wheat lines with spring growth habit and subsequent F_2_ and BC_1_F_2_ populations, and we found that the *VRN-A1* and *VRN-B1* genes controlled the segregation of growth habit. The durum cultivar Lebsock carried the dominant *Vrn-A1* and the recessive *vrn-B1* alleles, whereas PI 94749 had the recessive *vrn-A1* and the dominant *Vrn-B1* alleles. Because one dominant gene, either *Vrn-A1* or *Vrn-B1*, was sufficient to confer spring growth habit in the parental lines, the appearance of winter lines in the LP749 population was due to the homoeoallelic recombination. All of the DH lines with the double-recessive genotype (*vrn-A1vrn-B1*) had winter growth habit, demonstrating the decisive role of the *VRN1* gene in determining winter/spring growth habit in tetraploid wheat.

Alleles with variation in the first intron of *VRN-A1* are the same for tetraploid and hexaploid wheat (Fu *et al.* 2005). However, the allelic variant of *VRN-B1* identified in tetraploid wheat in this study is different from that previously reported in hexaploid wheat. Allelic variation in *VRN-B1* was reported to occur in intron 1 due to a large deletion in the dominant allele in extensive spring cultivars (Fu *et al.* 2005; Zhang *et al.* 2008), and no allelic variation in this gene was reported in its promoter or any other region. In this study, no variation was observed within the first intron of dominant and recessive *VRN-B1* alleles. Instead, the variation in *VRN-B1* was due to the insertion of the retrotrans_VRN element within the 5′ UTR of *Vrn-B1* in a position near the corresponding CArG-box and VRN-box regulatory sites of *VRN-A1* in diploid and hexaploid wheat (Yan *et al.* 2003, 2004a; Dubcovsky *et al.* 2006; Pidal *et al.* 2009).

Kane *et al.* (2007) reported that the MADS-box transcription factor TaVRT2 binds the CArG-box in the promoter region of the recessive *vrn1* allele. Li and Dubcovsky (2008) showed that TaFDL2, a functional homolog of *Arabidopsis* FD, can also interact with the promoter of the recessive *vrn1* allele. In both cases *vrn1* cannot be transcribed due to the binding of these transcriptional repressor(s) in the promoter region. Therefore, homozygous recessive *vrn1* cannot flower without vernalization. Deletions in the promoter region disrupt the ability of repressors to bind, allowing *Vrn1* to be transcribed and function in a dominant fashion, which results in spring growth habit (Yan *et al.* 2003; Dubcovsky *et al.* 2006; Pidal *et al.* 2009). In the same way, the insertion of retrotrans_VRN in the critical promoter region of the recessive *vrn-B1* allele may disrupt repressor binding, allowing the gene to be transcribed. The gene expression experiment proved that the *Vrn-B1* allele containing retrotrans_VRN was transcribed and the spring lines carrying the *Vrn-B1* allele flowered without vernalization. In the winter DH lines carrying the recessive *vrn-B1* allele, *vrn-B1* transcripts were detected only after plants were vernalized, and the vernalized plants flowered earlier than those without vernalization treatment did. Most insertions have deleterious effects on the expression of nearby genes in plants (Hollister *et al.* 2011). On the contrary, this study provides an example of an insertion that resulted in the liberation of gene expression. Mutation of the *VRN-B1* gene is the mechanism by which tetraploid wheat evolved from the winter wild type to spring type.

A survey of 154 spring-type accessions/lines of six *T*. *turgidum* subspecies indicated that none of the accessions from the subspecies *durum*, *polonicum*, *turanicum*, or *turgidum* had retrotrans_VRN in *VRN-B1*, but the insertion was present in some accessions of *T*. *turgidum* subsp. *dicoccum* and in most accessions of *T*. *turgidum* subsp. *carthlicum*. This suggested that the insertion event in the dominant *Vrn-B1* allele might have occurred during the evolution of tetraploid wheat and may have contributed to adaptation to certain environments. Identification of the *VRN-B1* allele in a global collection of tetraploid wheat could provide important molecular information for wheat breeding. Both *T. turgidum* subsp. *carthlicum* and *T. turgidum* subsp. *dicoccum* are potentially important sources for improving resistance to tan spot and Stagonospora nodorum blotch (Chu *et al.* 2008a), *Fusarium* head blight (Oliver *et al.* 2008), and stem rust (Olivera Firpo *et al.* 2010) for durum and bread wheat grown in North America. Molecular identification of the dominant gene for spring growth habit will facilitate selection of the proper wheat parents to breed cultivars for specific environments or to develop spring populations that do not have a vernalization requirement.

Effects of different *VRN1* genes on heading date have been studied extensively in spring wheat (Law *et al.* 1976; Stelmakh 1993; Kato *et al.* 1999; Tóth *et al.* 2003; Hanocq *et al.* 2004; Chu *et al.* 2008b). The dominant *Vrn-A1* allele normally provides complete insensitivity to vernalization; thus, it is considered the most potent for spring growth habit, whereas a dominant *Vrn-B1* or *Vrn-D1* allele may result in the partial elimination of the vernalization requirement (Pugsley 1971, 1972; Stelmakh 1993). *Vrn-A1* is indeed expressed at earlier and higher levels than *Vrn-B1* and *Vrn-D1* in Triple Dirk isogenic lines (Loukoianov *et al.* 2005). The Triple Dirk A (TDA) line carrying the dominant *Vrn-A1* allele flowered several days earlier than the Triple Dirk B (TDB) line carrying the dominant *Vrn-B1* allele and the Triple Dirk E (TDE) line carrying the dominant *Vrn-D1* allele (Trevaskis *et al.* 2003; Loukoianov *et al.* 2005). In this study, PI 94749, which harbors only the dominant *Vrn-B1* allele, headed up to two weeks later than Lebsock, which had only the dominant *Vrn-A1* allele (Table S3). However, in the DH population, the lines that carried only the dominant *Vrn-B1* allele had similar heading dates as the lines that only carried the dominant *Vrn-A1* (Table S3). It seems that the effect of the retrotrans_VRN–containing *Vrn-B1* allele on growth habit might be similar to that of *Vrn-A1* in tetraploid wheat. No further experiments were conducted to explain variation in flowering within the spring or winter groups, as this research was focused on the identification of allelic variation between winter lines that had a vernalization requirement and spring lines that lacked this requirement for flowering.

## Supplementary Material

Supporting Information
