# Supplementary material for: A Novel Retrotransposon Inserted in the Dominant Vrn-B1 Allele Confers Spring Growth Habit in Tetraploid Wheat (Triticum turgidum L.)
Source: G3 (Bethesda). 2011 Dec 1;1(7):637–45. doi: 10.1534/g3.111.001131 (PMC3276170; doi:10.1534/g3.111.001131)
Supplement: Supporting Information [file supp_1_7_637__index.html]

Supporting Information 

# A Novel Retrotransposon Inserted in the Dominant *Vrn-B1* Allele Confers Spring Growth Habit in Tetraploid Wheat (*Triticum turgidum* L.)

## Supporting Information for Chu *et al.*, 2011

**Files in this Data Supplement:**

- Supporting Information - Figures S1-S7 and Table S1-S3 (PDF, 8.9 MB)
- Figure S1 - DNA sequence comparison indicates the same 484-bp fragment amplified by primer pair *VRN1AF/VRN1AR* in both Lebsock (LB) and PI 94749 (PI), which indicated no sequence variation in the promoter region of the *VRN-A1* gene of the two parental lines (PDF, 624 KB)
- Figure S2 - Sequence comparison of the 1149-bp fragment amplified by the primer pair Intr1/B/F with Intr1/B/R4 indicates no sequence variation within the first intron of the *VRN-B1* gene in Lebsock (LB) and PI 94749 (PI) (PDF, 1.5 MB)
- Figure S3 - DNA sequence comparison indicates that the 989-bp fragment produced by the primer pair VRNBPF1/VRNBPR1 in Lebsock is the same as the reported *VRN-B1* gene in durum wheat �Langdon� (BAC clone 1225D16, GeneBank accession number AY616453) (YAN et al. 2004a) (PDF, 1.3 MB)
- Figure S4 - Comparison of DNA sequence in the 872-bp fragment produced by primer pair VRNBPF1/VRNBP R2 in Lebsock (LB) and PI 94749 (PI) suggests no allelic variation in the corresponding region close to *VRN-B1* promoter (PDF, 1.2 MB)
- Figure S5 - DNA sequence of the 5,463-bp insertion in the dominant *Vrn-B1* allele carried by PI 94749 (PDF, 2.9 MB)
- Figure S6 - The 1,231 amino acids in the deduced protein predicted by the web-based computer program GenScan at GeniusNet (http://genome.dkfz-heidelberg.de/cgi-bin/GENSCAN/genscan.cgi) (PDF, 684 KB)
- Figure S7 - Alignment of the sequences contains 5�- and 3�- ends of retrotrans\_VRN in PI 94749 and other 22 tetraploid wheat accessions (PDF, 164 KB)
- Table S1 - Winter/spring growth habit in the 146 double haploid (DH) lines derived from the cross between durum wheat 'Lebsock' and *T. turgidum* subsp. *carthlicum* accession PI 94749 (PDF, 68 KB)
- Table S2 - Frequency of *Vrn-B1* allele containing retrotrans\_VRN in 154 spring type accessions or lines from six tetraploid wheat (*Triticum turgidum* L.) sub-species (PDF, 180 KB)
- Table S3 - The growth habit and days to heading of the DH lines that were used in the expression experiment for *VRN-1* genes (PDF, 68 KB)
