# Supplementary material for: A Novel Retrotransposon Inserted in the Dominant Vrn-B1 Allele Confers Spring Growth Habit in Tetraploid Wheat (Triticum turgidum L.)
Source: G3 (Bethesda). 2011 Dec 1;1(7):637–45. doi: 10.1534/g3.111.001131 (PMC3276170; doi:10.1534/g3.111.001131)
Supplement: Supporting Information [file supp_1.7.637_001131SI.pdf]

```
PI   GAAAGGAAAAATTCTGCTCGTTTTTTTTCTCTGTGGTGTGTGTTTGTGGCGAGAGAAAAT
LB   GAAAGGAAAAATTCTGCTCGTTTTTTTTCTCTGTGGTGTGTGTTTGTGGCGAGAGAAAAT
     ************************************************************

PI   GATTTGGGGAAAGCAAAATCCGGAGATTCGCACGTACGATCGTTCGACACGTCGACGCCC
LB   GATTTGGGGAAAGCAAAATCCGGAGATTCGCACGTACGATCGTTCGACACGTCGACGCCC
     ************************************************************

PI   GGCGGGCCCGGGGTGGGGCATCGTGTGGCTGCAGGACCGCGGGGCCCCGCAAAGCGGGCC
LB   GGCGGGCCCGGGGTGGGGCATCGTGTGGCTGCAGGACCGCGGGGCCCCGCAAAGCGGGCC
     ************************************************************

PI   GGGCCAATGGGTGCTCGACAGCGGCTATGCTCCAGACCAGCCCGGTATTGCATACCGCGC
LB   GGGCCAATGGGTGCTCGACAGCGGCTATGCTCCAGACCAGCCCGGTATTGCATACCGCGC
     ************************************************************

PI   TCGGGGCCAGATCCCTTTAAAAACCCCTCCCCCCCTGCCGGAATCCTCGTTTTGGCCTGG
LB   TCGGGGCCAGATCCCTTTAAAAACCCCTCCCCCCCTGCCGGAATCCTCGTTTTGGCCTGG
     ************************************************************

PI   CCATCCTCCCTCTCCTCCCCTCTCTTCCACCTCACGTCCTCACCCAACCACCTGATAGCC
LB   CCATCCTCCCTCTCCTCCCCTCTCTTCCACCTCACGTCCTCACCCAACCACCTGATAGCC
     ************************************************************

PI   ATGGCTCCGCCGCCTCGCCTCCGCCTGCGCCAGTCGGAGTAGCCGTCGCGGTCTGCCGGT
LB   ATGGCTCCGCCGCCTCGCCTCCGCCTGCGCCAGTCGGAGTAGCCGTCGCGGTCTGCCGGT
     ************************************************************

PI   GTTGGAGGGTAGGGGCGTAGGGTTGGCCCGGTTCTCGAGCGGAGATGGGGCGGGGGAAGG
LB   GTTGGAGGGTAGGGGCGTAGGGTTGGCCCGGTTCTCGAGCGGAGATGGGGCGGGGGAAGG
     ************************************************************

PI   TGCA
LB   TGCA
     ****
```

Figure S1 DNA sequence comparison indicates the same 484-bp fragment amplified by primer pair VRN1AF/VRN1AR in both Lebsock (LB) and PI 94749 (PI), which indicated no sequence variation in the promoter region of the *VRN-A1* gene of the two parental lines.

```
PI   CAAGTGGAACGGTTAGGACAGTAATCTCTTGATATTTTTATCTGGCTGGGGATATTTACGTAAAAAAATTAT
LB   CAAGTGGAACGGTTAGGACAGTAATCTCTTGATATTTTTATCTGGCTGGGGATATTTACGTAAAAAAATTAT
     ************************************************************************

PI   ATGGGGTTAAAGTGACATCGCAATTTAGCATGCTACCTCATCTTCTCATTTAGAATCTTACTAGACGCTACA
LB   ATGGGGTTAAAGTGACATCGCAATTTAGCATGCTACCTCATCTTCTCATTTAGAATCTTACTAGACGCTACA
     ************************************************************************

PI   ATACCTTGTTGTCTGGCTCATCAAATCTGTGCTTGCTGCTTGAACAAATGAACCTCGTCATCTCGGTTATTT
LB   ATACCTTGTTGTCTGGCTCATCAAATCTGTGCTTGCTGCTTGAACAAATGAACCTCGTCATCTCGGTTATTT
     ************************************************************************

PI   CCAGAATTTTGTTCCACAGGCTTCCCTATCATTCGTATTGCTAGCTCCGGCCATGCGGCCATTTTGTTGCTT
LB   CCAGAATTTTGTTCCACAGGCTTCCCTATCATTCGTATTGCTAGCTCCGGCCATGCGGCCATTTTGTTGCTT
     ************************************************************************

PI   GCCTGGAGATACTGTCTACGGCACGCACGGAGAAAAGAGTCACTTGACTAGCTAATGCATGGAATAATTGTC
LB   GCCTGGAGATACTGTCTACGGCACGCACGGAGAAAAGAGTCACTTGACTAGCTAATGCATGGAATAATTGTC
     ************************************************************************

PI   TGCAGCTGATGAAACTCCGGCATGAAGAGTCAAACCAAAAAGTAGAGAGTTCCTTCCAAATATAAAATGAGA
LB   TGCAGCTGATGAAACTCCGGCATGAAGAGTCAAACCAAAAAGTAGAGAGTTCCTTCCAAATATAAAATGAGA
     ************************************************************************

PI   GTTTCTGCAGACTTTTTCCCTTTCAACCATCATAATTTGCCTGTGATATTTGTTGGTGCTGGCGATGGTTCT
LB   GTTTCTGCAGACTTTTTCCCTTTCAACCATCATAATTTGCCTGTGATATTTGTTGGTGCTGGCGATGGTTCT
     ************************************************************************

PI   TGACAAAGTAAAGGAGTCAATAAAATCACGGAGACTGATCCATTCTTTCCCCCACACGCTGACATTAGTCCA
LB   TGACAAAGTAAAGGAGTCAATAAAATCACGGAGACTGATCCATTCTTTCCCCCACACGCTGACATTAGTCCA
     ************************************************************************

PI   TGTTAGTTTCCCGTTTCTGCCTGCTTCCATAATTCCCGGCCGGCGAAGTACTAGATCAACCTCCACGGTTTC
LB   TGTTAGTTTCCCGTTTCTGCCTGCTTCCATAATTCCCGGCCGGCGAAGTACTAGATCAACCTCCACGGTTTC
     ************************************************************************

PI   AAAAAGTAGGAAATATCATACCATCGGAATGACCGCTGCTTAGTAAATATCCATTGTTGTTTGTAATCTTGC
LB   AAAAAGTAGGAAATATCATACCATCGGAATGACCGCTGCTTAGTAAATATCCATTGTTGTTTGTAATCTTGC
     ************************************************************************

PI   TGAGAAAGCAACGTTACCATTTGCCTCATGGCAAAGACCTGTATGTTGAGGTGCTAAATCTTTTCTAGTTTT
LB   TGAGAAAGCAACGTTACCATTTGCCTCATGGCAAAGACCTGTATGTTGAGGTGCTAAATCTTTTCTAGTTTT
     ************************************************************************

PI   GTACCACTGAGGGTATGAGTGGCGCTAACGGAAAAGGGTAAGCAAGTTTGATTGGCTTACCTTCAGCCTCCT
LB   GTACCACTGAGGGTATGAGTGGCGCTAACGGAAAAGGGTAAGCAAGTTTGATTGGCTTACCTTCAGCCTCCT
     ************************************************************************

PI   TGGTTGTTTGAAGCATAGGTGCTTGCATGCATGTATCAAGCTGGTCACGTGATGAAAACGCGTAAGAATCAA
LB   TGGTTGTTTGAAGCATAGGTGCTTGCATGCATGTATCAAGCTGGTCACGTGATGAAAACGCGTAAGAATCAA
     ************************************************************************

PI   AGTCAGTTAAATTAAGATATAAACAGATGCAGTCATATTTTAAGCTAGTGCTGCACTGTGAACTTCAGTATC
LB   AGTCAGTTAAATTAAGATATAAACAGATGCAGTCATATTTTAAGCTAGTGCTGCACTGTGAACTTCAGTATC
     ************************************************************************

PI   TCAGATCAAAGAATTGAATAATGCTACCCCCTGTTTCTGCGCTGTTCATTTGGAAAAGACTGTCATGAACAT
LB   TCAGATCAAAGAATTGAATAATGCTACCCCCTGTTTCTGCGCTGTTCATTTGGAAAAGACTGTCATGAACAT
     ************************************************************************

PI   CCTAATTGGTAGCCATGCATTTATCAGCTTGCCGGCTTTATTTTCTTTGCTCTCATTCCTTTTCATTTG
LB   CCTAATTGGTAGCCATGCATTTATCAGCTTGCCGGCTTTATTTTCTTTGCTCTCATTCCTTTTCATTTG
     *********************************************************************
```

Figure S2 Sequence comparison of the 1149-bp fragment amplified by the primer pair Intr1/B/F with Intr1/B/R4 indicates no sequence variation within the first intron of the *VRN-B1* gene in Lebsock (LB) and PI 94749 (PI).

```
AY   CCCCTGCTACCAGTGCCTACTACTAGGACGGGCGAGTATCTTCATTCATTCCCGAAATACGCGGGTCGGCCA
LB   CCCCTGCTACCAGTGCCTACTACTAGGACGGGCGAGTATCTTCATTCATTCCCGAAATACGCGGGTCGGCCA
     ************************************************************************

AY   AAAGTAGAAAAATGCACTGCGCCCACCCAACCCACGCAGCGCACTGCACAGTAACGCTTCCTGTCAAAAGTC
LB   AAAGTAGAAAAATGCACTGCGCCCACCCAACCCACGCAGCGCACTGCACAGTAACGCTTCCTGTCAAAAGTC
     ************************************************************************

AY   CAGCTCAATCATGCACGCACACACGGTAGACGCGGTGCGAACGACCCGTCGTGGCACGAGCACGCGGTGTCT
LB   CAGCTCAATCATGCACGCACACACGGTAGACGCGGTGCGAACGACCCGTCGTGGCACGAGCACGCGGTGTCT
     ************************************************************************

AY   GCCCGCCCGTCCGCCCCGCAGCCGCCCTCCCAAACGGGACAAGCTAGACGGCCCAAAACAAGAAAGGAAAGC
LB   GCCCGCCCGTCCGCCCCGCAGCCGCCCTCCCAAACGGGACAAGCTAGACGGCCCAAAACAAGAAAGGAAAGC
     ************************************************************************

AY   AGCCTCCTACTGTGGCAGCCCGCCCCCACGACCGTCATCTCGCCTTCCATGCCATTTTCCCTGGACGGACAG
LB   AGCCTCCTACTGTGGCAGCCCGCCCCCACGACCGTCATCTCGCCTTCCATGCCATTTTCCCTGGACGGACAG
     ************************************************************************

AY   ACCCGTCCGAGCCGCCCTGACCTAGCCAGCCAGCCAGCCAGCATTTCCTGTTTCGTCCCGCGCCGCCGTGAC
LB   ACCCGTCCGAGCCGCCCTGACCTAGCCAGCCAGCCAGCCAGCATTTCCTGTTTCGTCCCGCGCCGCCGTGAC
     ************************************************************************

AY   CAAAAAAGCAAAAAATTAAAAAGGAAAATGCTAAAGGAAAAACTCTGCTCTTTCCCTTCTACTAGGCCTAGG
LB   CAAAAAAGCAAAAAATTAAAAAGGAAAATGCTAAAGGAAAAACTCTGCTCTTTCCCTTCTACTAGGCCTAGG
     ************************************************************************

AY   GTACAGTAGAATAGTAGTATAAAAAGGACAATTGTGCTCTTTTTTTTTGCTCTGTGGTGTGTGTTTGTGGCG
LB   GTACAGTAGAATAGTAGTATAAAAAGGACAATTGTGCTCTTTTTTTTTGCTCTGTGGTGTGTGTTTGTGGCG
     ************************************************************************

AY   AGAGAAAATGATTTGGGGAAAGCAATATCGGGAGATTCGCACGTAAGATCGTTCGACACGTCGACACCGGGC
LB   AGAGAAAATGATTTGGGGAAAGCAATATCGGGAGATTCGCACGTAAGATCGTTCGACACGTCGACACCGGGC
     ************************************************************************

AY   GGGCCCGTGGTGGGGCATCGTGTGGCTGCAGTACCGCGGGGCCCCGCGGGTCGGGCTGGGCCAATGGTTGCT
LB   GGGCCCGTGGTGGGGCATCGTGTGGCTGCAGTACCGCGGGGCCCCGCGGGTCGGGCTGGGCCAATGGTTGCT
     ************************************************************************

AY   CGACAGCGGCTATGCTGCAGACCAGCCCGGTATTGCATACCGCGCTCGGGGCCAGATCCCTTTAAAAACCCC
LB   CGACAGCGGCTATGCTGCAGACCAGCCCGGTATTGCATACCGCGCTCGGGGCCAGATCCCTTTAAAAACCCC
     ************************************************************************

AY   TCCCCCCACTTGCCGGAAACCTCGTTTTGGCCTGGCCATCCTCCCTCTCCTCCCTCTCTTCCGCCTCACCCA
LB   TCCCCCCACTTGCCGGAAACCTCGTTTTGGCCTGGCCATCCTCCCTCTCCTCCCTCTCTTCCGCCTCACCCA
     ************************************************************************

AY   ACCACCTGACAGCCATGGCTCCGCCCCCCCGCCCCCGCCTGCGCCTGTCGGAGTAGCCGTCGCGGTCTGCCG
LB   ACCACCTGACAGCCATGGCTCCGCCCCCCCGCCCCCGCCTGCGCCTGTCGGAGTAGCCGTCGCGGTCTGCCG
     ************************************************************************

AY   GTGTTGGAGGCTTGGGGTGTAGGGTTGGCCCCGTTCTCCAGCGGAGATGGGGC
LB   GTGTTGGAGGCTTGGGGTGTAGGGTTGGCCCCGTTCTCCAGCGGAGATGGGGC
     *****************************************************
```

Figure S3 DNA sequence comparison indicates that the 989-bp fragment produced by the primer pair VRNBPF1/VRNBPR1 in Lebsock is the same as the reported *VRN-B1* gene in durum wheat 'Langdon' (BAC clone 1225D16, GeneBank accession number AY616453) (Yan et al. 2004a). LB and AY represents Lebsock and AY616453, respectively.

```
PI   CCCCTGCTACCAGTGCCTACTACTAGGACGGGCGAGTATCTTCATTCATTCCCGAAATACGCGGGTCGGCC
LB   CCCCTGCTACCAGTGCCTACTACTAGGACGGGCGAGTATCTTCATTCATTCCCGAAATACGCGGGTCGGCC
     ***********************************************************************

PI   AAAAGTAGAAAAATGCACTGCGCCCACCCAACCCACGCAGCGCACTGCACAGTAACGCTTCCTGTCAAAAG
LB   AAAAGTAGAAAAATGCACTGCGCCCACCCAACCCACGCAGCGCACTGCACAGTAACGCTTCCTGTCAAAAG
     ***********************************************************************

PI   TCCAGCTCAATCATGCACGCACACACGGTAGACGCGGTGCGAACGACCCGTCGTGGCACGAGCACGCGGTG
LB   TCCAGCTCAATCATGCACGCACACACGGTAGACGCGGTGCGAACGACCCGTCGTGGCACGAGCACGCGGTG
     ***********************************************************************

PI   TCTGCCCGCCCGTCCGCCCCGCAGCCGCCCTCCCAAACGGGACAAGCTAGACGGCCCAAAACAAGAAAGGA
LB   TCTGCCCGCCCGTCCGCCCCGCAGCCGCCCTCCCAAACGGGACAAGCTAGACGGCCCAAAACAAGAAAGGA
     ***********************************************************************

PI   AAGCAGCCTCCTACTGTGGCAGCCCGCCCCCACGACCGTCATCTCGCCTTCCATGCCATTTTCCCTGGACG
LB   AAGCAGCCTCCTACTGTGGCAGCCCGCCCCCACGACCGTCATCTCGCCTTCCATGCCATTTTCCCTGGACG
     ***********************************************************************

PI   GACAGACCCGTCCGAGCCGCCCTGACCTAGCCAGCCAGCCAGCCAGCATTTCCTGTTTCGTCCCGCGCCGC
LB   GACAGACCCGTCCGAGCCGCCCTGACCTAGCCAGCCAGCCAGCCAGCATTTCCTGTTTCGTCCCGCGCCGC
     ***********************************************************************

PI   CGTGACCAAAAAAGCAAAAAATTAAAAAGGAAAATGCTAAAGGAAAAACTCTGCTCTTTCCCTTCTACTAG
LB   CGTGACCAAAAAAGCAAAAAATTAAAAAGGAAAATGCTAAAGGAAAAACTCTGCTCTTTCCCTTCTACTAG
     ***********************************************************************

PI   GCCTAGGGTACAGTAGAATAGTAGTATAAAAAGGACAATTGTGCTCTTTTTTTTTGCTCTGTGGTGTGTGT
LB   GCCTAGGGTACAGTAGAATAGTAGTATAAAAAGGACAATTGTGCTCTTTTTTTTTGCTCTGTGGTGTGTGT
     ***********************************************************************

PI   TTGTGGCGAGAGAAAATGATTTGGGGAAAGCAATATCGGGAGATTCGCACGTAAGATCGTTCGACACGTCG
LB   TTGTGGCGAGAGAAAATGATTTGGGGAAAGCAATATCGGGAGATTCGCACGTAAGATCGTTCGACACGTCG
     ***********************************************************************

PI   ACACCGGGCGGGCCCGTGGTGGGGCATCGTGTGGCTGCAGTACCGCGGGGCCCCGCGGGTCGGGCTGGGCC
LB   ACACCGGGCGGGCCCGTGGTGGGGCATCGTGTGGCTGCAGTACCGCGGGGCCCCGCGGGTCGGGCTGGGCC
     ***********************************************************************

PI   AATGGTTGCTCGACAGCGGCTATGCTGCAGACCAGCCCGGTATTGCATACCGCGCTCGGGGCCAGATCCCT
LB   AATGGTTGCTCGACAGCGGCTATGCTGCAGACCAGCCCGGTATTGCATACCGCGCTCGGGGCCAGATCCCT
     ***********************************************************************

PI   TTAAAAACCCCTCCCCCCACTTGCCGGAAACCTCGTTTTGGCCTGGCCATCCTCCCTCTCCTCCCTCTCTT
LB   TTAAAAACCCCTCCCCCCACTTGCCGGAAACCTCGTTTTGGCCTGGCCATCCTCCCTCTCCTCCCTCTCTT
     ***********************************************************************

PI   CCGCCTCACCCAACCACCTG
LB   CCGCCTCACCCAACCACCTG
     ********************
```

Figure S4 Comparison of DNA sequence in the 872-bp fragment produced by primer pair VRNBPF1/VRNBP R2 in Lebsock (LB) and PI 94749 (PI) suggests no allelic variation in the corresponding region close to *VRN-B1* promoter.

CTCCGTGTCACGACTGTCAAGGTGACCTCACCAGCCAACAAGCGGGAAGAGGAACTGGTCAAGGCGCTTGCTGATTCGACAGAAGGGCCGAC

CCGTTAGCAGGCCGCGGTGTCTTGGGTGCGTGGCCTTGTGGGCCGTATCGAGCCTGCGAGCTCAGGGTTAAGTAAGTGGCTTGGCACCGTCA

AGGGCATCAAATTGTATCTCACTGAATAGACAGAGAGAAGCTGCTCTTCTCCTGGTTATCCCCTTCCTTCTCCACCTACCTCAATCTCACCT

GCCCCCTTCCTCCTCCTCTCAGATCTGATCGATTAGGTAGATCGGGCCGTTACAAGTGGTATCAGAGGTCGGGCGATCTGGATAACCCGTAC

PBS

CGCAAGCCGCGCTACGCCACCAAGCAAGTTACGCAAGCTGCAGCGATGGAGGAGCAATTGGCGGCGCTGGCCAAGGCGGTCAACGACGGCCG

CACCGCCGACGAGGCCCGGCTCGAGGCCATCCAGACCTCGCTCGAGTTGTGGCGTCCAGCGGTCACCAACCTGCAACAACAACTCAACGAGC

TCCAATCCCAAGTGGGGCGGATCGCACTCCACCCCGCGCTGGCAGATCCGCAACAGCCACCGGTGGAACAAGTGGTGCACGGCGCGCCGACC

GAGTCGGCGGGCGACTTCGAGCACCACGGGCCATCTGGCCACGGCGAGATCGACAAAACCGGGGGTCGGGCACACGGGGTAGTCACTACCCT

CGCGCCACCTCCGGTCAAGGGTGCGTACTCATCCCAATCTATCATACCTGCTTCTCCTCGCGGTGATAGTGGTCCAGAGGCGGAAAGGAGGG

GCACTCAGGACAACCCGTTCGTTCCATTCGCGCATCACGCTCACTGGGCTCTGCCCAAGATGGATTTTCCATCGTTTGATGGAGAAAATCCG

CAGTTCTGGAAGACCAAATGTGAAAAGTATTTTGATGTGTATGGAGTGGCTCCGGATCTCTGGGTTCGATTAGCCACGCTGAATTTCACAGG

CACAGCGGCCAGATGGCTGCAACTTCATGAGACACAGAGCACTTCATTCACTTGGGCGTCACTCTGTGAGGCCTTGTGCCATAAATTTGGTA

GAGAACAGTACCAGTCCCATCTCCGTCAGTTTAACACCCTTCGTCAGTCAGGGACTGTAGCGGATTACATGACACGATTTGAAGAATTGATG

CATCACATATTAGCTCACAACCCAGCGTTCGACTCCGTGTATTTTACTACCCAGTTTCTAGATGGTCTTAAGGGAGAGATTCGTGCTGTTGT

TATGCTTCATCAACCAAAAGACTTGGACTCCGCCTTTTCCTTGGCTACATTGCAGGAGGAGCTGATGGAGGCGTTACCGCGAAGGGAGTACA

AGCGACAGGATGCAGCTAACCAGCGGTCACCTGCACAACGGCCTTTGCTGGCGATTGGTGCTCCGCCCGTCCGGCAGGTGTTGCCAGGACCG

CCACCTGCAGCTGAAGATCGACGGGCGATCGATGCGGCAAACCCTCCGGATCGCCGCGATCAAGGCAGAGGTGATGATCGGGTAGCAGCCCT

CCGTAACTACAGGCGTGCTCGGGGGCTCTGTTTTAAATGTGGTGAGCGATGGGGCCAAGGGCATCAATGCGGGCCTACAGTTCAATTACATG

TGGTCGAAGAACTGCTAGAACTGTTGCAAGCTGATCAGGGCGTTCCAGTAGTGCCTGACCCTGATTCTGATGAAGATGTTCTGATGTGCATC

TCCAAGGGAGCCACCACGGGGCAAACTACTCCCCGTACAGTCAGGTTGCTGGGGCAGATAGGTGGACAAGAGATGTTGATCCTCGTGGATTC

GGGCAGCTCTCATAGCTTTCTCAGTGATACAGTGGTGGCGCGACTTCAACTACCAATTCAAGCCATGTCCACGGTTGCAGTCAAAATAGCAG

ACGGGGGAACTCTATCATGTTCAGGTGTGGTGCCAGAGTGCAGATGGAAGACACAAGGACATGAATTTGTCACTGACCTCAGAGTTTTAGCG

CTTGGGTGCTATGATATGATAGTTGGCATGGACTGGCTGGAGTCATGTGGGCCTATGTGGATCGATTGGTCCGCAAAGCAACTGATATTCAA

CCACGGCGGGCAGCAAATTCAGTTGGCAGGGGTGCAAACGCAATTGCGACAAGTCCAACCAATCTCCTCGGCTCAGTTGTGTGCACTGGAAG

AGGCTAATGCGGTGGCTCACATCATCTGTTTGCATGCGGTGGGGGATGATGTAGTGGTGGAACACATTCCAGTGGAAGTACAAGCTGTGCTG

CAAGAATACAGTGTGGTGTTTGAAAAGCCTACTGATCTACCTCCACACCGCGCTTGGGACCATGCAATTCCAATCATCCCTGGAGCCAAGCC

GGTCAACATTCGACCGTATCGGTATACCCCGGAACAAAAGACAGAGATTGAGCTTCAGGTGAAGGAAATGCTTAAAGCTGGACTAATCGTGC

CTAGTACCAGTCCGTTCTCCTCGCCTGTTCTGTTAGTGAAAAAGAAAGATATGACATGGCGCCTCTGCGTGGACTATCGTCACCTGAATGCA

ATTACACTAAAAAGCACCTATCCATTGCCAGTCATTGACGAGCTGCTCGATGAGCTTGCAGGATCGTGTTGGTTCTCAAAGATGGATCTACG

AGCGGGCTACCACCAAATCAGGTTGAGAGAAGAAGATGAACCAAAAACAGCCTTTACAACCCATCAAGGGCACTTTCAGTTTCGGGTGTTGC

CGTATGGTGTGACCGGAGGGCCAGCAACATTCCAAGGAGGGATGAACACAGTGCTGGGTCCTTTGCTCAGGCACGGTGTGTGTGTTTTCATG

GACGACATCCTCACCCACTCAGCAACCTTAGAGGGACACGTGGAACTGCTGCGGCAAGTGTTGAGTATTTTGGCACAACATGGTTTGAAGGT

CAAAATGTCCAAATGCTCCTTTGCACAACGCAAGATCGATTTCTTGGGGCACACCATAAGCAAGGAAGGTGTAACCACTGATGAGAGCAAGA

TTGCAACAGTCCGGGACTGGCCGCGCCCAGGGTCAGTTCGCGAGCTACGAGGCTTCTTGGGGCTTGCGGGATACTACCGTAAGTTCGTCAGA

AACTTCGGCGTCATATCCCGTCCTCTGACAGACATGTTGAAGAAGGGCACCCTCTTTATTTGGACACCGCTAGCAGAAACAGCATTTGCAGA

GTTGAAGCAGGCACTCATCCAGGCCCCCGTGCTCGCATTACCTGATTTCAACAAGAAGTTCGTCGTAGAGACTGACGCAAGTGCCAAGGGCG

TCGGTGCAGTCCTGATGCAGGACTTCCACCCGTTAGCTTACTTGAGCAAGGCGTTAGCGCCACGCAACCTTGGTCTATCAGCATATGAAAAA

GAGTGTCTTGCCTTGATTTTAGCAGTTGATCACTGGAGGCCGTATTTACAGCATGCCGAGTTTCTGGTGCGCACCGATCAAAAGAGCTTGCT

GAATCTGACCGACCAGAGGCTCAACACACCAATCCAGCAGCGGGCTTTCACCAAGTTAGTGGGGCTGCAGTTCCAGATCCAGTACAAGGCAG

GCATCACTAACAAAGCAGCAGATGCGCTCTCACGGCGCGAACATGACACTGAAGCAGCAGTGGCAGCGATTTCAATTTGCAAGCCCGCCTGG

TTGGAAGCAGTGGCCGTCAGTTATCGCGAAGACAAAGAAATTCAAGACAAGATGGCGCAAATCGCACTAGATCCTGGAAGTGACTCGGATTA

CTCTCTGAAGGATGGAGTCATGCGTTACAAGGGGCGTATCTGGATTGGCTCGGATAGTATGATACAACAATCGCTGGTCAAGGCGCTCCACG

ACAGTGCAGTGGGTGGACATTCAGGGTTCTACGCCACGTATCACAGAATCAAAAACCTCTTCTTCTGGAAAGGCATGAAGGCTCAGATTAAG

CAATATGTCAAGGAGTGTGTCACTTGTCAGCGTGCGAAAACAGAACGAATCGCTCCAGCGGGGCTGTTACAACCCCTCCCCATTCCAAAGCG

GCCATGGGCAGTGATCTCCCTGGATTTTATTGAAGGCCTTCCCAAAATCCGGAGGCCATGATGTGATTTTGGTCGTGGTGGATAAGTTCTCC

AAATATGCTCACTTCGTTCCACTGACTCATCCATTCACGGCTCTGACGGTGGCTACGGCCTTCATGAAAAACATCTTTCGGCTTCACGGGCT

GCCTCTAGCTATTATATCTGACCGAGACCGGATCTTCACAAGCACAATTTGGCAAGAGTTGTTCAAACTGTCTCAGACCCAGCTGCGTTTGA

GTTCGTCATACCATCCCCAAACTGATGGGCAGACAGAGCGCGTAAACCAGTGCTTAGAAGGATACCTGCGCTGTGCAGTGCACTCGTGTCCT

GGTAATTGGATCAAGTGGCTATTCTTAGCCGAGTATTGGTATAACACGACATTTCACTCCTCTCTGGGGCGCACTCCATTTGAAGTAATCTA

TGGCCATCTGCCAAGGGAGTTCGCTGTTACTCAAGTGGAAGAAAGTTCAGTGCCGGACCTGGCAGCTTGGCTACAGGAACGAGAAGTGATGG

CTCAGCACTTACAGCAACAACTGAAACATGCTCAGGATCGCATGAAAGCTCAGGCCGACAAACACCGCACTGACCGTTCATTCGAAGTGGGT

GACATGGTTTTCCTCAAGCTCCAGCCACCATATACAAACATCCGTAGCCCAACGCCCTTACCAGAAAGCTGGCATTCCGCTACTACGGGCCC

ATACCCAAGTGCTCGCTCGGATCGGAAAAAGTGGCATACAAACTTCAGTTACCAGCTGACAGTAAGATACATTCCGTGGTGCACGTCTCACA

ACTAAAAAAGGCTGTTGGTCCTAGCACCCCAGGTGAGCTGCGATCTTCCTCCTGTTAACTCTATTCTGCAGGCTGAACATCAACCAGAGGCA

ATTCTGGACACCAAGTTCATCCGCGTCCGGGGAGAGATGCAACCTCGTCTTCTGGTACAATGGGGTGCGCTGCCGGCGTCGCTCGCCACCTG

GGAGGAGCCCGCTGAACTGCGTCGTCGCTTTCCAGCAGCATCGGCTTGGGGACAAGCCTCACCTCAAGGGGGGGAGGATGTCACGACTGTCA

PPT

AGGTGACCTCACCAGCCAACAAGCGGGAAGAGGAACTGGTCAAGGCGCTTGCTGATTCGACAGAAGGGCCGACCCGTTAGCAGGCCGCGGTG

TCTTGGGTGCGTGGCCTTGTGGGCCGTATCGAGCCTGCGAGCTCAGGGTTAAGTAAGTGGCTTGGCACCGTCAAGGGCATCAAATTGTATCT

CACTGAATAGACAGAGAGAAGCTGCTCTTCTCCTGGTTATCCCCTTCCTTCTCCACCTACCTCAATCTCACCTGCCCCCTTCCTCCTCCTCT

CAGATCTGATCGATTAGGTAGATCGGGCCGTTACA**CTCCG**

Figure S5 DNA sequence of the 5,463-bp insertion in the dominant *Vrn-B1* allele carried by PI 94749. The two CTCCG motifs on both ends of the insertion are shown in bold and highlighted in gray. The short inverted repeat 5′-TG...CA-3′ on the ends of the long terminal repeat (LTR) is highlighted in green, and LTR is shown in red. The motifs primer binding site (PBS) and polypurine tract (PPT) are underlined but highlighted in light blue and purple, respectively. For sequence annotation of the insertion that predicted by computer program GenScan at GeniusNet, the start codon (ATG) and stop codon (TGA) are shown in red with yellow highlight, and an open reading frame is highlighted in gray. The 1,231 amino acids encoded by the deduced coding

region are shown in Figure S6. The sequence data of the insertion has been deposited with the GenBank Data Libraries under accession number HQ186251.

```
MEEQLAALAKAVNDGRTADEARLEAIQTSLELWRPAVTNLQQQLNELQSQVGRIALHPALADPQQPPVEQV
VHGAPTESAGDFEHHGPSGHGEIDKTGGRAHGVVTTLAPPPVKGAYSSQSIIPASPRGDSGPEAERRGTQD
NPFVPFAHHAHWALPKMDFPSFDGENPQFWKTKCEKYFDVYGVAPDLWVRLATLNFTGTAARWLQLHETQS
TSFTWASLCEALCHKFGREQYQSHLRQFNTLRQSGTVADYMTRFEELMHHILAHNPAFDSVYFTTQFLDGL
KGEIRAVVMLHQPKDLDSAFSLATLQEELMEALPRREYKRQDAANQRSPAQRPLLAIGAPPVRQVLPGPPP
AAEDRRAIDAANPPDRRDQGRGDDRVAALRNYRRARGLCFKCGERWGQGHQCGPTVQLHVVEELLELLQAD
QGVPVVPDPDSDEDVLMCISKGATTGQTTPRTVRLLGQIGGQEMLILVDSGSSHSFLSDTVVARLQLPIQA
MSTVAVKIADGGTLSCSGVVPECRWKTQGHEFVTDLRVLALGCYDMIVGMDWLESCGPMWIDWSAKQLIFN
HGGQQIQLAGVQTQLRQVQPISSAQLCALEEANAVAHIICLHAVGDDVVVEHIPVEVQAVLQEYSVVFEKP
TDLPPHRAWDHAIPIIPGAKPVNIRPYRYTPEQKTEIELQVKEMLKAGLIVPSTSPFSSPVLLVKKKDMTW
RLCVDYRHLNAITLKSTYPLPVIDELLDELAGSCWFSKMDLRAGYHQIRLREEDEPKTAFTTHQGHFQFRV
LPYGVTGGPATFQGGMNTVLGPLLRHGVCVFMDDILTHSATLEGHVELLRQVLSILAQHGLKVKMSKCSFA
QRKIDFLGHTISKEGVTTDESKIATVRDWPRPGSVRELRGFLGLAGYYRKFVRNFGVISRPLTDMLKKGTL
FIWTPLAETAFAELKQALIQAPVLALPDFNKKFVVETDASAKGVGAVLMQDFHPLAYLSKALAPRNLGLSA
YEKECLALILAVDHWRPYLQHAEFLVRTDQKSLLNLTDQRLNTPIQQRAFTKLVGLQFQIQYKAGITNKAA
DALSRREHDTEAAVAAISICKPAWLEAVAVSYREDKEIQDKMAQIALDPGSDSDYSLKDGVMRYKGRIWIG
SDSMIQQSLVKALHDSAVGGHSGFYATYHRIKNLFFWKGMKAQIKQYVKECVTCQRAKTERIAPAGLLQPL
PIPKRPWAVISLDFIEGLPKIRRP*
```

Figure S6 The 1,231 amino acids in the deduced protein predicted by the web-based computer program GenScan at GeniusNet (http://genome.dkfz-heidelberg.de/cgi-bin/GENSCAN/genscan.cgi).

Comparison of 600-bp sequence amplified by primers VBINS2F (5’-CTCACCCAACCACCTGACAGC-3’) and VBINS2R (5’-ATCCGCCCCACTTGGGATT-3’):

```
PI 499972       CTCACCCAACCACCTGACAGCCATGGCTCCGTGTCACGACTGTCAAGGTGACCTCACCAG
PI 532501       CTCACCCAACCACCTGACAGCCATGGCTCCGTGTCACGACTGTCAAGGTGACCTCACCAG
PI 352278       CTCACCCAACCACCTGACAGCCATGGCTCCGTGTCACGACTGTCAAGGTGACCTCACCAG
PI 115816       CTCACCCAACCACCTGACAGCCATGGCTCCGTGTCACGACTGTCAAGGTGACCTCACCAG
PI 283887       CTCACCCAACCACCTGACAGCCATGGCTCCGTGTCACGACTGTCAAGGTGACCTCACCAG
PS5             CTCACCCAACCACCTGACAGCCATGGCTCCGTGTCACGACTGTCAAGGTGACCTCACCAG
CItr 7779       CTCACCCAACCACCTGACAGCCATGGCTCCGTGTCACGACTGTCAAGGTGACCTCACCAG
CItr 3686       CTCACCCAACCACCTGACAGCCATGGCTCCGTGTCACGACTGTCAAGGTGACCTCACCAG
PI 113961       CTCACCCAACCACCTGACAGCCATGGCTCCGTGTCACGACTGTCAAGGTGACCTCACCAG
PI 585017       CTCACCCAACCACCTGACAGCCATGGCTCCGTGTCACGACTGTCAAGGTGACCTCACCAG
PI 532486       CTCACCCAACCACCTGACAGCCATGGCTCCGTGTCACGACTGTCAAGGTGACCTCACCAG
PI 532475       CTCACCCAACCACCTGACAGCCATGGCTCCGTGTCACGACTGTCAAGGTGACCTCACCAG
PI 470732       CTCACCCAACCACCTGACAGCCATGGCTCCGTGTCACGACTGTCAAGGTGACCTCACCAG
PI 352281       CTCACCCAACCACCTGACAGCCATGGCTCCGTGTCACGACTGTCAAGGTGACCTCACCAG
PI 349040       CTCACCCAACCACCTGACAGCCATGGCTCCGTGTCACGACTGTCAAGGTGACCTCACCAG
PI 283889       CTCACCCAACCACCTGACAGCCATGGCTCCGTGTCACGACTGTCAAGGTGACCTCACCAG
PI 251914       CTCACCCAACCACCTGACAGCCATGGCTCCGTGTCACGACTGTCAAGGTGACCTCACCAG
PI 61102        CTCACCCAACCACCTGACAGCCATGGCTCCGTGTCACGACTGTCAAGGTGACCTCACCAG
PI 352282       CTCACCCAACCACCTGACAGCCATGGCTCCGTGTCACGACTGTCAAGGTGACCTCACCAG
PI 286070       CTCACCCAACCACCTGACAGCCATGGCTCCGTGTCACGACTGTCAAGGTGACCTCACCAG
PI 115817       CTCACCCAACCACCTGACAGCCATGGCTCCGTGTCACGACTGTCAAGGTGACCTCACCAG
PI 94749        CTCACCCAACCACCTGACAGCCATGGCTCCGTGTCACGACTGTCAAGGTGACCTCACCAG
PI 94748        CTCACCCAACCACCTGACAGCCATGGCTCCGTGTCACGACTGTCAAGGTGACCTCACCAG
                ************************************************************

PI 499972       CCAACAAGCGGGAAGAGGAACTGGTCAAGGCGCTTGCTGATTCGACAGAAGGGCCGACCC
PI 532501       CCAACAAGCGGGAAGAGGAACTGGTCAAGGCGCTTGCTGATTCGACAGAAGGGCCGACCC
PI 352278       CCAACAAGCGGGAAGAGGAACTGGTCAAGGCGCTTGCTGATTCGACAGAAGGGCCGACCC
PI 115816       CCAACAAGCGGGAAGAGGAACTGGTCAAGGCGCTTGCTGATTCGACAGAAGGGCCGACCC
PI 283887       CCAACAAGCGGGAAGAGGAACTGGTCAAGGCGCTTGCTGATTCGACAGAAGGGCCGACCC
PS5             CCAACAAGCGGGAAGAGGAACTGGTCAAGGCGCTTGCTGATTCGACAGAAGGGCCGACCC
CItr 7779       CCAACAAGCGGGAAGAGGAACTGGTCAAGGCGCTTGCTGATTCGACAGAAGGGCCGACCC
CItr 3686       CCAACAAGCGGGAAGAGGAACTGGTCAAGGCGCTTGCTGATTCGACAGAAGGGCCGACCC
PI 113961       CCAACAAGCGGGAAGAGGAACTGGTCAAGGCGCTTGCTGATTCGACAGAAGGGCCGACCC
PI 585017       CCAACAAGCGGGAAGAGGAACTGGTCAAGGCGCTTGCTGATTCGACAGAAGGGCCGACCC
PI 532486       CCAACAAGCGGGAAGAGGAACTGGTCAAGGCGCTTGCTGATTCGACAGAAGGGCCGACCC
PI 532475       CCAACAAGCGGGAAGAGGAACTGGTCAAGGCGCTTGCTGATTCGACAGAAGGGCCGACCC
PI 470732       CCAACAAGCGGGAAGAGGAACTGGTCAAGGCGCTTGCTGATTCGACAGAAGGGCCGACCC
PI 352281       CCAACAAGCGGGAAGAGGAACTGGTCAAGGCGCTTGCTGATTCGACAGAAGGGCCGACCC
PI 349040       CCAACAAGCGGGAAGAGGAACTGGTCAAGGCGCTTGCTGATTCGACAGAAGGGCCGACCC
PI 283889       CCAACAAGCGGGAAGAGGAACTGGTCAAGGCGCTTGCTGATTCGACAGAAGGGCCGACCC
PI 251914       CCAACAAGCGGGAAGAGGAACTGGTCAAGGCGCTTGCTGATTCGACAGAAGGGCCGACCC
PI 61102        CCAACAAGCGGGAAGAGGAACTGGTCAAGGCGCTTGCTGATTCGACAGAAGGGCCGACCC
PI 352282       CCAACAAGCGGGAAGAGGAACTGGTCAAGGCGCTTGCTGATTCGACAGAAGGGCCGACCC
PI 286070       CCAACAAGCGGGAAGAGGAACTGGTCAAGGCGCTTGCTGATTCGACAGAAGGGCCGACCC
PI 115817       CCAACAAGCGGGAAGAGGAACTGGTCAAGGCGCTTGCTGATTCGACAGAAGGGCCGACCC
PI 94749        CCAACAAGCGGGAAGAGGAACTGGTCAAGGCGCTTGCTGATTCGACAGAAGGGCCGACCC
PI 94748        CCAACAAGCGGGAAGAGGAACTGGTCAAGGCGCTTGCTGATTCGACAGAAGGGCCGACCC
                ************************************************************

PI 499972       GTTAGCAGGCCGCGGTGTCTTGGGTGCGTGGCCTTGTGGGCCGTATCGAGCCTGCGAGCT
PI 532501       GTTAGCAGGCCGCGGTGTCTTGGGTGCGTGGCCTTGTGGGCCGTATCGAGCCTGCGAGCT
PI 352278       GTTAGCAGGCCGCGGTGTCTTGGGTGCGTGGCCTTGTGGGCCGTATCGAGCCTGCGAGCT
PI 115816       GTTAGCAGGCCGCGGTGTCTTGGGTGCGTGGCCTTGTGGGCCGTATCGAGCCTGCGAGCT
```

```
PI 283887        GTTAGCAGGCCGCGGTGTCTTGGGTGCGTGGCCTTGTGGGCCGTATCGAGCCTGCGAGCT
PS5              GTTAGCAGGCCGCGGTGTCTTGGGTGCGTGGCCTTGTGGGCCGTATCGAGCCTGCGAGCT
CItr 7779        GTTAGCAGGCCGCGGTGTCTTGGGTGCGTGGCCTTGTGGGCCGTATCGAGCCTGCGAGCT
CItr 3686        GTTAGCAGGCCGCGGTGTCTTGGGTGCGTGGCCTTGTGGGCCGTATCGAGCCTGCGAGCT
PI 113961        GTTAGCAGGCCGCGGTGTCTTGGGTGCGTGGCCTTGTGGGCCGTATCGAGCCTGCGAGCT
PI 585017        GTTAGCAGGCCGCGGTGTCTTGGGTGCGTGGCCTTGTGGGCCGTATCGAGCCTGCGAGCT
PI 532486        GTTAGCAGGCCGCGGTGTCTTGGGTGCGTGGCCTTGTGGGCCGTATCGAGCCTGCGAGCT
PI 532475        GTTAGCAGGCCGCGGTGTCTTGGGTGCGTGGCCTTGTGGGCCGTATCGAGCCTGCGAGCT
PI 470732        GTTAGCAGGCCGCGGTGTCTTGGGTGCGTGGCCTTGTGGGCCGTATCGAGCCTGCGAGCT
PI 352281        GTTAGCAGGCCGCGGTGTCTTGGGTGCGTGGCCTTGTGGGCCGTATCGAGCCTGCGAGCT
PI 349040        GTTAGCAGGCCGCGGTGTCTTGGGTGCGTGGCCTTGTGGGCCGTATCGAGCCTGCGAGCT
PI 283889        GTTAGCAGGCCGCGGTGTCTTGGGTGCGTGGCCTTGTGGGCCGTATCGAGCCTGCGAGCT
PI 251914        GTTAGCAGGCCGCGGTGTCTTGGGTGCGTGGCCTTGTGGGCCGTATCGAGCCTGCGAGCT
PI 61102         GTTAGCAGGCCGCGGTGTCTTGGGTGCGTGGCCTTGTGGGCCGTATCGAGCCTGCGAGCT
PI 352282        GTTAGCAGGCCGCGGTGTCTTGGGTGCGTGGCCTTGTGGGCCGTATCGAGCCTGCGAGCT
PI 286070        GTTAGCAGGCCGCGGTGTCTTGGGTGCGTGGCCTTGTGGGCCGTATCGAGCCTGCGAGCT
PI 115817        GTTAGCAGGCCGCGGTGTCTTGGGTGCGTGGCCTTGTGGGCCGTATCGAGCCTGCGAGCT
PI 94749         GTTAGCAGGCCGCGGTGTCTTGGGTGCGTGGCCTTGTGGGCCGTATCGAGCCTGCGAGCT
PI 94748         GTTAGCAGGCCGCGGTGTCTTGGGTGCGTGGCCTTGTGGGCCGTATCGAGCCTGCGAGCT
                 ************************************************************

PI 499972        CAGGGTTAAGTAAGTGGCTTGGCACCGTCAAGGGCATCAAATTGTATCTCACTGAATAGA
PI 532501        CAGGGTTAAGTAAGTGGCTTGGCACCGTCAAGGGCATCAAATTGTATCTCACTGAATAGA
PI 352278        CAGGGTTAAGTAAGTGGCTTGGCACCGTCAAGGGCATCAAATTGTATCTCACTGAATAGA
PI 115816        CAGGGTTAAGTAAGTGGCTTGGCACCGTCAAGGGCATCAAATTGTATCTCACTGAATAGA
PI 283887        CAGGGTTAAGTAAGTGGCTTGGCACCGTCAAGGGCATCAAATTGTATCTCACTGAATAGA
PS5              CAGGGTTAAGTAAGTGGCTTGGCACCGTCAAGGGCATCAAATTGTATCTCACTGAATAGA
CItr 7779        CAGGGTTAAGTAAGTGGCTTGGCACCGTCAAGGGCATCAAATTGTATCTCACTGAATAGA
CItr 3686        CAGGGTTAAGTAAGTGGCTTGGCACCGTCAAGGGCATCAAATTGTATCTCACTGAATAGA
PI 113961        CAGGGTTAAGTAAGTGGCTTGGCACCGTCAAGGGCATCAAATTGTATCTCACTGAATAGA
PI 585017        CAGGGTTAAGTAAGTGGCTTGGCACCGTCAAGGGCATCAAATTGTATCTCACTGAATAGA
PI 532486        CAGGGTTAAGTAAGTGGCTTGGCACCGTCAAGGGCATCAAATTGTATCTCACTGAATAGA
PI 532475        CAGGGTTAAGTAAGTGGCTTGGCACCGTCAAGGGCATCAAATTGTATCTCACTGAATAGA
PI 470732        CAGGGTTAAGTAAGTGGCTTGGCACCGTCAAGGGCATCAAATTGTATCTCACTGAATAGA
PI 352281        CAGGGTTAAGTAAGTGGCTTGGCACCGTCAAGGGCATCAAATTGTATCTCACTGAATAGA
PI 349040        CAGGGTTAAGTAAGTGGCTTGGCACCGTCAAGGGCATCAAATTGTATCTCACTGAATAGA
PI 283889        CAGGGTTAAGTAAGTGGCTTGGCACCGTCAAGGGCATCAAATTGTATCTCACTGAATAGA
PI 251914        CAGGGTTAAGTAAGTGGCTTGGCACCGTCAAGGGCATCAAATTGTATCTCACTGAATAGA
PI 61102         CAGGGTTAAGTAAGTGGCTTGGCACCGTCAAGGGCATCAAATTGTATCTCACTGAATAGA
PI 352282        CAGGGTTAAGTAAGTGGCTTGGCACCGTCAAGGGCATCAAATTGTATCTCACTGAATAGA
PI 286070        CAGGGTTAAGTAAGTGGCTTGGCACCGTCAAGGGCATCAAATTGTATCTCACTGAATAGA
PI 115817        CAGGGTTAAGTAAGTGGCTTGGCACCGTCAAGGGCATCAAATTGTATCTCACTGAATAGA
PI 94749         CAGGGTTAAGTAAGTGGCTTGGCACCGTCAAGGGCATCAAATTGTATCTCACTGAATAGA
PI 94748         CAGGGTTAAGTAAGTGGCTTGGCACCGTCAAGGGCATCAAATTGTATCTCACTGAATAGA
                 ************************************************************


PI 499972        CAGAGAGAAGCTGCTCTTCTCCTGGTTATCCCCTTCCTTCTCCACCTACCTCAATCTCAC
PI 532501        CAGAGAGAAGCTGCTCTTCTCCTGGTTATCCCCTTCCTTCTCCACCTACCTCAATCTCAC
PI 352278        CAGAGAGAAGCTGCTCTTCTCCTGGTTATCCCCTTCCTTCTCCACCTACCTCAATCTCAC
PI 115816        CAGAGAGAAGCTGCTCTTCTCCTGGTTATCCCCTTCCTTCTCCACCTACCTCAATCTCAC
PI 283887        CAGAGAGAAGCTGCTCTTCTCCTGGTTATCCCCTTCCTTCTCCACCTACCTCAATCTCAC
PS5              CAGAGAGAAGCTGCTCTTCTCCTGGTTATCCCCTTCCTTCTCCACCTACCTCAATCTCAC
CItr 7779        CAGAGAGAAGCTGCTCTTCTCCTGGTTATCCCCTTCCTTCTCCACCTACCTCAATCTCAC
CItr 3686        CAGAGAGAAGCTGCTCTTCTCCTGGTTATCCCCTTCCTTCTCCACCTACCTCAATCTCAC
PI 113961        CAGAGAGAAGCTGCTCTTCTCCTGGTTATCCCCTTCCTTCTCCACCTACCTCAATCTCAC
```

```
PI 585017       CAGAGAGAAGCTGCTCTTCTCCTGGTTATCCCCTTCCTTCTCCACCTACCTCAATCTCAC
PI 532486       CAGAGAGAAGCTGCTCTTCTCCTGGTTATCCCCTTCCTTCTCCACCTACCTCAATCTCAC
PI 532475       CAGAGAGAAGCTGCTCTTCTCCTGGTTATCCCCTTCCTTCTCCACCTACCTCAATCTCAC
PI 470732       CAGAGAGAAGCTGCTCTTCTCCTGGTTATCCCCTTCCTTCTCCACCTACCTCAATCTCAC
PI 352281       CAGAGAGAAGCTGCTCTTCTCCTGGTTATCCCCTTCCTTCTCCACCTACCTCAATCTCAC
PI 349040       CAGAGAGAAGCTGCTCTTCTCCTGGTTATCCCCTTCCTTCTCCACCTACCTCAATCTCAC
PI 283889       CAGAGAGAAGCTGCTCTTCTCCTGGTTATCCCCTTCCTTCTCCACCTACCTCAATCTCAC
PI 251914       CAGAGAGAAGCTGCTCTTCTCCTGGTTATCCCCTTCCTTCTCCACCTACCTCAATCTCAC
PI 61102        CAGAGAGAAGCTGCTCTTCTCCTGGTTATCCCCTTCCTTCTCCACCTACCTCAATCTCAC
PI 352282       CAGAGAGAAGCTGCTCTTCTCCTGGTTATCCCCTTCCTTCTCCACCTACCTCAATCTCAC
PI 286070       CAGAGAGAAGCTGCTCTTCTCCTGGTTATCCCCTTCCTTCTCCACCTACCTCAATCTCAC
PI 115817       CAGAGAGAAGCTGCTCTTCTCCTGGTTATCCCCTTCCTTCTCCACCTACCTCAATCTCAC
PI 94749        CAGAGAGAAGCTGCTCTTCTCCTGGTTATCCCCTTCCTTCTCCACCTACCTCAATCTCAC
PI 94748        CAGAGAGAAGCTGCTCTTCTCCTGGTTATCCCCTTCCTTCTCCACCTACCTCAATCTCAC
                ************************************************************

PI 499972       CTGCCCCCTTCCTCCTCCTCTCAGATCTGATCGATTAGGTAGATCGGGCCGTTACAAGTG
PI 532501       CTGCCCCCTTCCTCCTCCTCTCAGATCTGATCGATTAGGTAGATCGGGCCGTTACAAGTG
PI 352278       CTGCCCCCTTCCTCCTCCTCTCAGATCTGATCGATTAGGTAGATCGGGCCGTTACAAGTG
PI 115816       CTGCCCCCTTCCTCCTCCTCTCAGATCTGATCGATTAGGTAGATCGGGCCGTTACAAGTG
PI 283887       CTGCCCCCTTCCTCCTCCTCTCAGATCTGATCGATTAGGTAGATCGGGCCGTTACAAGTG
PS5             CTGCCCCCTTCCTCCTCCTCTCAGATCTGATCGATTAGGTAGATCGGGCCGTTACAAGTG
CItr 7779       CTGCCCCCTTCCTCCTCCTCTCAGATCTGATCGATTAGGTAGATCGGGCCGTTACAAGTG
CItr 3686       CTGCCCCCTTCCTCCTCCTCTCAGATCTGATCGATTAGGTAGATCGGGCCGTTACAAGTG
PI 113961       CTGCCCCCTTCCTCCTCCTCTCAGATCTGATCGATTAGGTAGATCGGGCCGTTACAAGTG
PI 585017       CTGCCCCCTTCCTCCTCCTCTCAGATCTGATCGATTAGGTAGATCGGGCCGTTACAAGTG
PI 532486       CTGCCCCCTTCCTCCTCCTCTCAGATCTGATCGATTAGGTAGATCGGGCCGTTACAAGTG
PI 532475       CTGCCCCCTTCCTCCTCCTCTCAGATCTGATCGATTAGGTAGATCGGGCCGTTACAAGTG
PI 470732       CTGCCCCCTTCCTCCTCCTCTCAGATCTGATCGATTAGGTAGATCGGGCCGTTACAAGTG
PI 352281       CTGCCCCCTTCCTCCTCCTCTCAGATCTGATCGATTAGGTAGATCGGGCCGTTACAAGTG
PI 349040       CTGCCCCCTTCCTCCTCCTCTCAGATCTGATCGATTAGGTAGATCGGGCCGTTACAAGTG
PI 283889       CTGCCCCCTTCCTCCTCCTCTCAGATCTGATCGATTAGGTAGATCGGGCCGTTACAAGTG
PI 251914       CTGCCCCCTTCCTCCTCCTCTCAGATCTGATCGATTAGGTAGATCGGGCCGTTACAAGTG
PI 61102        CTGCCCCCTTCCTCCTCCTCTCAGATCTGATCGATTAGGTAGATCGGGCCGTTACAAGTG
PI 352282       CTGCCCCCTTCCTCCTCCTCTCAGATCTGATCGATTAGGTAGATCGGGCCGTTACAAGTG
PI 286070       CTGCCCCCTTCCTCCTCCTCTCAGATCTGATCGATTAGGTAGATCGGGCCGTTACAAGTG
PI 115817       CTGCCCCCTTCCTCCTCCTCTCAGATCTGATCGATTAGGTAGATCGGGCCGTTACAAGTG
PI 94749        CTGCCCCCTTCCTCCTCCTCTCAGATCTGATCGATTAGGTAGATCGGGCCGTTACAAGTG
PI 94748        CTGCCCCCTTCCTCCTCCTCTCAGATCTGATCGATTAGGTAGATCGGGCCGTTACAAGTG
                ************************************************************


PI 499972       GTATCAGAGGTCGGGCGATCTGGATAACCCGTACCGCAAGCCGCGCTACGCCACCAAGCA
PI 532501       GTATCAGAGGTCGGGCGATCTGGATAACCCGTACCGCAAGCCGCGCTACGCCACCAAGCA
PI 352278       GTATCAGAGGTCGGGCGATCTGGATAACCCGTACCGCAAGCCGCGCTACGCCACCAAGCA
PI 115816       GTATCAGAGGTCGGGCGATCTGGATAACCCGTACCGCAAGCCGCGCTACGCCACCAAGCA
PI 283887       GTATCAGAGGTCGGGCGATCTGGATAACCCGTACCGCAAGCCGCGCTACGCCACCAAGCA
PS5             GTATCAGAGGTCGGGCGATCTGGATAACCCGTACCGCAAGCCGCGCTACGCCACCAAGCA
CItr 7779       GTATCAGAGGTCGGGCGATCTGGATAACCCGTACCGCAAGCCGCGCTACGCCACCAAGCA
CItr 3686       GTATCAGAGGTCGGGCGATCTGGATAACCCGTACCGCAAGCCGCGCTACGCCACCAAGCA
PI 113961       GTATCAGAGGTCGGGCGATCTGGATAACCCGTACCGCAAGCCGCGCTACGCCACCAAGCA
PI 585017       GTATCAGAGGTCGGGCGATCTGGATAACCCGTACCGCAAGCCGCGCTACGCCACCAAGCA
PI 532486       GTATCAGAGGTCGGGCGATCTGGATAACCCGTACCGCAAGCCGCGCTACGCCACCAAGCA
PI 532475       GTATCAGAGGTCGGGCGATCTGGATAACCCGTACCGCAAGCCGCGCTACGCCACCAAGCA
PI 470732       GTATCAGAGGTCGGGCGATCTGGATAACCCGTACCGCAAGCCGCGCTACGCCACCAAGCA
PI 352281       GTATCAGAGGTCGGGCGATCTGGATAACCCGTACCGCAAGCCGCGCTACGCCACCAAGCA
```

```
PI 349040       GTATCAGAGGTCGGGCGATCTGGATAACCCGTACCGCAAGCCGCGCTACGCCACCAAGCA
PI 283889       GTATCAGAGGTCGGGCGATCTGGATAACCCGTACCGCAAGCCGCGCTACGCCACCAAGCA
PI 251914       GTATCAGAGGTCGGGCGATCTGGATAACCCGTACCGCAAGCCGCGCTACGCCACCAAGCA
PI 61102        GTATCAGAGGTCGGGCGATCTGGATAACCCGTACCGCAAGCCGCGCTACGCCACCAAGCA
PI 352282       GTATCAGAGGTCGGGCGATCTGGATAACCCGTACCGCAAGCCGCGCTACGCCACCAAGCA
PI 286070       GTATCAGAGGTCGGGCGATCTGGATAACCCGTACCGCAAGCCGCGCTACGCCACCAAGCA
PI 115817       GTATCAGAGGTCGGGCGATCTGGATAACCCGTACCGCAAGCCGCGCTACGCCACCAAGCA
PI 94749        GTATCAGAGGTCGGGCGATCTGGATAACCCGTACCGCAAGCCGCGCTACGCCACCAAGCA
PI 94748        GTATCAGAGGTCGGGCGATCTGGATAACCCGTACCGCAAGCCGCGCTACGCCACCAAGCA
                ************************************************************

PI 499972       AGTTACGCAAGCTGCAGCGATGGAGGAGCAATTGGCGGCGCTGGCCAAGGCGGTCAACGA
PI 532501       AGTTACGCAAGCTGCAGCGATGGAGGAGCAATTGGCGGCGCTGGCCAAGGCGGTCAACGA
PI 352278       AGTTACGCAAGCTGCAGCGATGGAGGAGCAATTGGCGGCGCTGGCCAAGGCGGTCAACGA
PI 115816       AGTTACGCAAGCTGCAGCGATGGAGGAGCAATTGGCGGCGCTGGCCAAGGCGGTCAACGA
PI 283887       AGTTACGCAAGCTGCAGCGATGGAGGAGCAATTGGCGGCGCTGGCCAAGGCGGTCAACGA
PS5             AGTTACGCAAGCTGCAGCGATGGAGGAGCAATTGGCGGCGCTGGCCAAGGCGGTCAACGA
CItr 7779       AGTTACGCAAGCTGCAGCGATGGAGGAGCAATTGGCGGCGCTGGCCAAGGCGGTCAACGA
CItr 3686       AGTTACGCAAGCTGCAGCGATGGAGGAGCAATTGGCGGCGCTGGCCAAGGCGGTCAACGA
PI 113961       AGTTACGCAAGCTGCAGCGATGGAGGAGCAATTGGCGGCGCTGGCCAAGGCGGTCAACGA
PI 585017       AGTTACGCAAGCTGCAGCGATGGAGGAGCAATTGGCGGCGCTGGCCAAGGCGGTCAACGA
PI 532486       AGTTACGCAAGCTGCAGCGATGGAGGAGCAATTGGCGGCGCTGGCCAAGGCGGTCAACGA
PI 532475       AGTTACGCAAGCTGCAGCGATGGAGGAGCAATTGGCGGCGCTGGCCAAGGCGGTCAACGA
PI 470732       AGTTACGCAAGCTGCAGCGATGGAGGAGCAATTGGCGGCGCTGGCCAAGGCGGTCAACGA
PI 352281       AGTTACGCAAGCTGCAGCGATGGAGGAGCAATTGGCGGCGCTGGCCAAGGCGGTCAACGA
PI 349040       AGTTACGCAAGCTGCAGCGATGGAGGAGCAATTGGCGGCGCTGGCCAAGGCGGTCAACGA
PI 283889       AGTTACGCAAGCTGCAGCGATGGAGGAGCAATTGGCGGCGCTGGCCAAGGCGGTCAACGA
PI 251914       AGTTACGCAAGCTGCAGCGATGGAGGAGCAATTGGCGGCGCTGGCCAAGGCGGTCAACGA
PI 61102        AGTTACGCAAGCTGCAGCGATGGAGGAGCAATTGGCGGCGCTGGCCAAGGCGGTCAACGA
PI 352282       AGTTACGCAAGCTGCAGCGATGGAGGAGCAATTGGCGGCGCTGGCCAAGGCGGTCAACGA
PI 286070       AGTTACGCAAGCTGCAGCGATGGAGGAGCAATTGGCGGCGCTGGCCAAGGCGGTCAACGA
PI 115817       AGTTACGCAAGCTGCAGCGATGGAGGAGCAATTGGCGGCGCTGGCCAAGGCGGTCAACGA
PI 94749        AGTTACGCAAGCTGCAGCGATGGAGGAGCAATTGGCGGCGCTGGCCAAGGCGGTCAACGA
PI 94748        AGTTACGCAAGCTGCAGCGATGGAGGAGCAATTGGCGGCGCTGGCCAAGGCGGTCAACGA
                ************************************************************

PI 499972       CGGCCGCACCGCCAACGAGGCCCGGCTCGAGGCCATCCAGACCTCGCTCGAGTTGTGGCG
PI 532501       CGGCCGCACCGCCAACGAGGCCCGGCTCGAGGCCATCCAGACCTCGCTCGAGTTGTGGCG
PI 352278       CGGCCGCACCGCCAACGAGGCCCGGCTCGAGGCCATCCAGACCTCGCTCGAGTTGTGGCG
PI 115816       CGGCCGCACCGCCAACGAGGCCCGGCTCGAGGCCATCCAGACCTCGCTCGAGTTGTGGCG
PI 283887       CGGCCGCACCGCCAACGAGGCCCGGCTCGAGGCCATCCAGACCTCGCTCGAGTTGTGGCG
PS5             CGGCCGCACCGCCAACGAGGCCCGGCTCGAGGCCATCCAGACCTCGCTCGAGTTGTGGCG
CItr 7779       CGGCCGCACCGCCAACGAGGCCCGGCTCGAGGCCATCCAGACCTCGCTCGAGTTGTGGCG
CItr 3686       CGGCCGCACCGCCAACGAGGCCCGGCTCGAGGCCATCCAGACCTCGCTCGAGTTGTGGCG
PI 113961       CGGCCGCACCGCCAACGAGGCCCGGCTCGAGGCCATCCAGACCTCGCTCGAGTTGTGGCG
PI 585017       CGGCCGCACCGCCAACGAGGCCCGGCTCGAGGCCATCCAGACCTCGCTCGAGTTGTGGCG
PI 532486       CGGCCGCACCGCCAACGAGGCCCGGCTCGAGGCCATCCAGACCTCGCTCGAGTTGTGGCG
PI 532475       CGGCCGCACCGCCAACGAGGCCCGGCTCGAGGCCATCCAGACCTCGCTCGAGTTGTGGCG
PI 470732       CGGCCGCACCGCCAACGAGGCCCGGCTCGAGGCCATCCAGACCTCGCTCGAGTTGTGGCG
PI 352281       CGGCCGCACCGCCAACGAGGCCCGGCTCGAGGCCATCCAGACCTCGCTCGAGTTGTGGCG
PI 349040       CGGCCGCACCGCCAACGAGGCCCGGCTCGAGGCCATCCAGACCTCGCTCGAGTTGTGGCG
PI 283889       CGGCCGCACCGCCAACGAGGCCCGGCTCGAGGCCATCCAGACCTCGCTCGAGTTGTGGCG
PI 251914       CGGCCGCACCGCCAACGAGGCCCGGCTCGAGGCCATCCAGACCTCGCTCGAGTTGTGGCG
PI 61102        CGGCCGCACCGCCAACGAGGCCCGGCTCGAGGCCATCCAGACCTCGCTCGAGTTGTGGCG
PI 352282       CGGCCGCACCGCCAACGAGGCCCGGCTCGAGGCCATCCAGACCTCGCTCGAGTTGTGGCG
```

```
PI 286070        CGGCCGCACCGCCAACGAGGCCCGGCTCGAGGCCATCCAGACCTCGCTCGAGTTGTGGCG
PI 115817        CGGCCGCACCGCCAACGAGGCCCGGCTCGAGGCCATCCAGACCTCGCTCGAGTTGTGGCG
PI 94749         CGGCCGCACCGCCAACGAGGCCCGGCTCGAGGCCATCCAGACCTCGCTCGAGTTGTGGCG
PI 94748         CGGCCGCACCGCCAACGAGGCCCGGCTCGAGGCCATCCAGACCTCGCTCGAGTTGTGGCG
                 ************************************************************

PI 499972        TCCAGCGGTCACCAACCTGCAACAACAACTCAACGAGCTCCAATCCCAAGTGGGGCGGAT
PI 532501        TCCAGCGGTCACCAACCTGCAACAACAACTCAACGAGCTCCAATCCCAAGTGGGGCGGAT
PI 352278        TCCAGCGGTCACCAACCTGCAACAACAACTCAACGAGCTCCAATCCCAAGTGGGGCGGAT
PI 115816        TCCAGCGGTCACCAACCTGCAACAACAACTCAACGAGCTCCAATCCCAAGTGGGGCGGAT
PI 283887        TCCAGCGGTCACCAACCTGCAACAACAACTCAACGAGCTCCAATCCCAAGTGGGGCGGAT
PS5              TCCAGCGGTCACCAACCTGCAACAACAACTCAACGAGCTCCAATCCCAAGTGGGGCGGAT
CItr 7779        TCCAGCGGTCACCAACCTGCAACAACAACTCAACGAGCTCCAATCCCAAGTGGGGCGGAT
CItr 3686        TCCAGCGGTCACCAACCTGCAACAACAACTCAACGAGCTCCAATCCCAAGTGGGGCGGAT
PI 113961        TCCAGCGGTCACCAACCTGCAACAACAACTCAACGAGCTCCAATCCCAAGTGGGGCGGAT
PI 585017        TCCAGCGGTCACCAACCTGCAACAACAACTCAACGAGCTCCAATCCCAAGTGGGGCGGAT
PI 532486        TCCAGCGGTCACCAACCTGCAACAACAACTCAACGAGCTCCAATCCCAAGTGGGGCGGAT
PI 532475        TCCAGCGGTCACCAACCTGCAACAACAACTCAACGAGCTCCAATCCCAAGTGGGGCGGAT
PI 470732        TCCAGCGGTCACCAACCTGCAACAACAACTCAACGAGCTCCAATCCCAAGTGGGGCGGAT
PI 352281        TCCAGCGGTCACCAACCTGCAACAACAACTCAACGAGCTCCAATCCCAAGTGGGGCGGAT
PI 349040        TCCAGCGGTCACCAACCTGCAACAACAACTCAACGAGCTCCAATCCCAAGTGGGGCGGAT
PI 283889        TCCAGCGGTCACCAACCTGCAACAACAACTCAACGAGCTCCAATCCCAAGTGGGGCGGAT
PI 251914        TCCAGCGGTCACCAACCTGCAACAACAACTCAACGAGCTCCAATCCCAAGTGGGGCGGAT
PI 61102         TCCAGCGGTCACCAACCTGCAACAACAACTCAACGAGCTCCAATCCCAAGTGGGGCGGAT
PI 352282        TCCAGCGGTCACCAACCTGCAACAACAACTCAACGAGCTCCAATCCCAAGTGGGGCGGAT
PI 286070        TCCAGCGGTCACCAACCTGCAACAACAACTCAACGAGCTCCAATCCCAAGTGGGGCGGAT
PI 115817        TCCAGCGGTCACCAACCTGCAACAACAACTCAACGAGCTCCAATCCCAAGTGGGGCGGAT
PI 94749         TCCAGCGGTCACCAACCTGCAACAACAACTCAACGAGCTCCAATCCCAAGTGGGGCGGAT
PI 94748         TCCAGCGGTCACCAACCTGCAACAACAACTCAACGAGCTCCAATCCCAAGTGGGGCGGAT
                 ************************************************************
```

Comparison of 615-bp sequence amplified by primers VBINS5F (5’-CAACCAGAGGCAATTCTGGACAC-3’) and VRNBPR1 (5’-GCCCCATCTCCGCTGGAGAACG-3’):

```
PI 499972      CAACCAGAGGCAATTCTGGACACCAAGTTCATCCGCGTCCGGGGAGAGATGCAACCTCGT
CItr 7779      CAACCAGAGGCAATTCTGGACACCAAGTTCATCCGCGTCCGGGGAGAGATGCAACCTCGT
PI 352281      CAACCAGAGGCAATTCTGGACACCAAGTTCATCCGCGTCCGGGGAGAGATGCAACCTCGT
PI 286070      CAACCAGAGGCAATTCTGGACACCAAGTTCATCCGCGTCCGGGGAGAGATGCAACCTCGT
PI 94748       CAACCAGAGGCAATTCTGGACACCAAGTTCATCCGCGTCCGGGGAGAGATGCAACCTCGT
PS5            CAACCAGAGGCAATTCTGGACACCAAGTTCATCCGCGTCCGGGGAGAGATGCAACCTCGT
PI 532501      CAACCAGAGGCAATTCTGGACACCAAGTTCATCCGCGTCCGGGGAGAGATGCAACCTCGT
PI 532475      CAACCAGAGGCAATTCTGGACACCAAGTTCATCCGCGTCCGGGGAGAGATGCAACCTCGT
PI 470732      CAACCAGAGGCAATTCTGGACACCAAGTTCATCCGCGTCCGGGGAGAGATGCAACCTCGT
PI 349040      CAACCAGAGGCAATTCTGGACACCAAGTTCATCCGCGTCCGGGGAGAGATGCAACCTCGT
PI 113961      CAACCAGAGGCAATTCTGGACACCAAGTTCATCCGCGTCCGGGGAGAGATGCAACCTCGT
CItr 3686      CAACCAGAGGCAATTCTGGACACCAAGTTCATCCGCGTCCGGGGAGAGATGCAACCTCGT
PI 585017      CAACCAGAGGCAATTCTGGACACCAAGTTCATCCGCGTCCGGGGAGAGATGCAACCTCGT
PI 532486      CAACCAGAGGCAATTCTGGACACCAAGTTCATCCGCGTCCGGGGAGAGATGCAACCTCGT
PI 352282      CAACCAGAGGCAATTCTGGACACCAAGTTCATCCGCGTCCGGGGAGAGATGCAACCTCGT
PI 352278      CAACCAGAGGCAATTCTGGACACCAAGTTCATCCGCGTCCGGGGAGAGATGCAACCTCGT
PI 283889      CAACCAGAGGCAATTCTGGACACCAAGTTCATCCGCGTCCGGGGAGAGATGCAACCTCGT
PI 251914      CAACCAGAGGCAATTCTGGACACCAAGTTCATCCGCGTCCGGGGAGAGATGCAACCTCGT
PI 115816      CAACCAGAGGCAATTCTGGACACCAAGTTCATCCGCGTCCGGGGAGAGATGCAACCTCGT
PI 61102       CAACCAGAGGCAATTCTGGACACCAAGTTCATCCGCGTCCGGGGAGAGATGCAACCTCGT
PI 283887      CAACCAGAGGCAATTCTGGACACCAAGTTCATCCGCGTCCGGGGAGAGATGCAACCTCGT
PI 94749       CAACCAGAGGCAATTCTGGACACCAAGTTCATCCGCGTCCGGGGAGAGATGCAACCTCGT
PI 115817      CAACCAGAGGCAATTCTGGACACCAAGTTCATCCGCGTCCGGGGAGAGATGCAACCTCGT
               ************************************************************

PI 499972      TCTTCTGGTACAATGGGGTGCGCTGCCGGCGTCGCTCGCCACCTGGGAGGAGCCCGCTGA
CItr 7779      TCTTCTGGTACAATGGGGTGCGCTGCCGGCGTCGCTCGCCACCTGGGAGGAGCCCGCTGA
PI 352281      TCTTCTGGTACAATGGGGTGCGCTGCCGGCGTCGCTCGCCACCTGGGAGGAGCCCGCTGA
PI 286070      TCTTCTGGTACAATGGGGTGCGCTGCCGGCGTCGCTCGCCACCTGGGAGGAGCCCGCTGA
PI 94748       TCTTCTGGTACAATGGGGTGCGCTGCCGGCGTCGCTCGCCACCTGGGAGGAGCCCGCTGA
PS5            TCTTCTGGTACAATGGGGTGCGCTGCCGGCGTCGCTCGCCACCTGGGAGGAGCCCGCTGA
PI 532501      TCTTCTGGTACAATGGGGTGCGCTGCCGGCGTCGCTCGCCACCTGGGAGGAGCCCGCTGA
PI 532475      TCTTCTGGTACAATGGGGTGCGCTGCCGGCGTCGCTCGCCACCTGGGAGGAGCCCGCTGA
PI 470732      TCTTCTGGTACAATGGGGTGCGCTGCCGGCGTCGCTCGCCACCTGGGAGGAGCCCGCTGA
PI 349040      TCTTCTGGTACAATGGGGTGCGCTGCCGGCGTCGCTCGCCACCTGGGAGGAGCCCGCTGA
PI 113961      TCTTCTGGTACAATGGGGTGCGCTGCCGGCGTCGCTCGCCACCTGGGAGGAGCCCGCTGA
CItr 3686      TCTTCTGGTACAATGGGGTGCGCTGCCGGCGTCGCTCGCCACCTGGGAGGAGCCCGCTGA
PI 585017      TCTTCTGGTACAATGGGGTGCGCTGCCGGCGTCGCTCGCCACCTGGGAGGAGCCCGCTGA
PI 532486      TCTTCTGGTACAATGGGGTGCGCTGCCGGCGTCGCTCGCCACCTGGGAGGAGCCCGCTGA
PI 352282      TCTTCTGGTACAATGGGGTGCGCTGCCGGCGTCGCTCGCCACCTGGGAGGAGCCCGCTGA
PI 352278      TCTTCTGGTACAATGGGGTGCGCTGCCGGCGTCGCTCGCCACCTGGGAGGAGCCCGCTGA
PI 283889      TCTTCTGGTACAATGGGGTGCGCTGCCGGCGTCGCTCGCCACCTGGGAGGAGCCCGCTGA
PI 251914      TCTTCTGGTACAATGGGGTGCGCTGCCGGCGTCGCTCGCCACCTGGGAGGAGCCCGCTGA
PI 115816      TCTTCTGGTACAATGGGGTGCGCTGCCGGCGTCGCTCGCCACCTGGGAGGAGCCCGCTGA
PI 61102       TCTTCTGGTACAATGGGGTGCGCTGCCGGCGTCGCTCGCCACCTGGGAGGAGCCCGCTGA
PI 283887      TCTTCTGGTACAATGGGGTGCGCTGCCGGCGTCGCTCGCCACCTGGGAGGAGCCCGCTGA
PI 94749       TCTTCTGGTACAATGGGGTGCGCTGCCGGCGTCGCTCGCCACCTGGGAGGAGCCCGCTGA
PI 115817      TCTTCTGGTACAATGGGGTGCGCTGCCGGCGTCGCTCGCCACCTGGGAGGAGCCCGCTGA
               ************************************************************

PI 499972      ACTGCGTCGTCGCTTTCCAGCAGCATCGGCTTGGGGACAAGCCTCACCTCAAGGGGGGGA
CItr 7779      ACTGCGTCGTCGCTTTCCAGCAGCATCGGCTTGGGGACAAGCCTCACCTCAAGGGGGGGA
PI 352281      ACTGCGTCGTCGCTTTCCAGCAGCATCGGCTTGGGGACAAGCCTCACCTCAAGGGGGGGA
PI 286070      ACTGCGTCGTCGCTTTCCAGCAGCATCGGCTTGGGGACAAGCCTCACCTCAAGGGGGGGA
```

PI 94748 ACTGCGTCGTCGCTTTCCAGCAGCATCGGCTTGGGGACAAGCCTCACCTCAAGGGGGGGA
PS5 ACTGCGTCGTCGCTTTCCAGCAGCATCGGCTTGGGGACAAGCCTCACCTCAAGGGGGGGA
PI 532501 ACTGCGTCGTCGCTTTCCAGCAGCATCGGCTTGGGGACAAGCCTCACCTCAAGGGGGGGA
PI 532475 ACTGCGTCGTCGCTTTCCAGCAGCATCGGCTTGGGGACAAGCCTCACCTCAAGGGGGGGA
PI 470732 ACTGCGTCGTCGCTTTCCAGCAGCATCGGCTTGGGGACAAGCCTCACCTCAAGGGGGGGA
PI 349040 ACTGCGTCGTCGCTTTCCAGCAGCATCGGCTTGGGGACAAGCCTCACCTCAAGGGGGGGA
PI 113961 ACTGCGTCGTCGCTTTCCAGCAGCATCGGCTTGGGGACAAGCCTCACCTCAAGGGGGGGA
CItr 3686 ACTGCGTCGTCGCTTTCCAGCAGCATCGGCTTGGGGACAAGCCTCACCTCAAGGGGGGGA
PI 585017 ACTGCGTCGTCGCTTTCCAGCAGCATCGGCTTGGGGACAAGCCTCACCTCAAGGGGGGGA
PI 532486 ACTGCGTCGTCGCTTTCCAGCAGCATCGGCTTGGGGACAAGCCTCACCTCAAGGGGGGGA
PI 352282 ACTGCGTCGTCGCTTTCCAGCAGCATCGGCTTGGGGACAAGCCTCACCTCAAGGGGGGGA
PI 352278 ACTGCGTCGTCGCTTTCCAGCAGCATCGGCTTGGGGACAAGCCTCACCTCAAGGGGGGGA
PI 283889 ACTGCGTCGTCGCTTTCCAGCAGCATCGGCTTGGGGACAAGCCTCACCTCAAGGGGGGGA
PI 251914 ACTGCGTCGTCGCTTTCCAGCAGCATCGGCTTGGGGACAAGCCTCACCTCAAGGGGGGGA
PI 115816 ACTGCGTCGTCGCTTTCCAGCAGCATCGGCTTGGGGACAAGCCTCACCTCAAGGGGGGGA
PI 61102 ACTGCGTCGTCGCTTTCCAGCAGCATCGGCTTGGGGACAAGCCTCACCTCAAGGGGGGGA
PI 283887 ACTGCGTCGTCGCTTTCCAGCAGCATCGGCTTGGGGACAAGCCTCACCTCAAGGGGGGGA
PI 94749 ACTGCGTCGTCGCTTTCCAGCAGCATCGGCTTGGGGACAAGCCTCACCTCAAGGGGGGGA
PI 115817 ACTGCGTCGTCGCTTTCCAGCAGCATCGGCTTGGGGACAAGCCTCACCTCAAGGGGGGGA
****

PI 499972 GGATGTCACGACTGTCAAGGTGACCTCACCAGCCAACAAGCGGGAAGAGGAACTGGTCAA
CItr 7779 GGATGTCACGACTGTCAAGGTGACCTCACCAGCCAACAAGCGGGAAGAGGAACTGGTCAA
PI 352281 GGATGTCACGACTGTCAAGGTGACCTCACCAGCCAACAAGCGGGAAGAGGAACTGGTCAA
PI 286070 GGATGTCACGACTGTCAAGGTGACCTCACCAGCCAACAAGCGGGAAGAGGAACTGGTCAA
PI 94748 GGATGTCACGACTGTCAAGGTGACCTCACCAGCCAACAAGCGGGAAGAGGAACTGGTCAA
PS5 GGATGTCACGACTGTCAAGGTGACCTCACCAGCCAACAAGCGGGAAGAGGAACTGGTCAA
PI 532501 GGATGTCACGACTGTCAAGGTGACCTCACCAGCCAACAAGCGGGAAGAGGAACTGGTCAA
PI 532475 GGATGTCACGACTGTCAAGGTGACCTCACCAGCCAACAAGCGGGAAGAGGAACTGGTCAA
PI 470732 GGATGTCACGACTGTCAAGGTGACCTCACCAGCCAACAAGCGGGAAGAGGAACTGGTCAA
PI 349040 GGATGTCACGACTGTCAAGGTGACCTCACCAGCCAACAAGCGGGAAGAGGAACTGGTCAA
PI 113961 GGATGTCACGACTGTCAAGGTGACCTCACCAGCCAACAAGCGGGAAGAGGAACTGGTCAA
CItr 3686 GGATGTCACGACTGTCAAGGTGACCTCACCAGCCAACAAGCGGGAAGAGGAACTGGTCAA
PI 585017 GGATGTCACGACTGTCAAGGTGACCTCACCAGCCAACAAGCGGGAAGAGGAACTGGTCAA
PI 532486 GGATGTCACGACTGTCAAGGTGACCTCACCAGCCAACAAGCGGGAAGAGGAACTGGTCAA
PI 352282 GGATGTCACGACTGTCAAGGTGACCTCACCAGCCAACAAGCGGGAAGAGGAACTGGTCAA
PI 352278 GGATGTCACGACTGTCAAGGTGACCTCACCAGCCAACAAGCGGGAAGAGGAACTGGTCAA
PI 283889 GGATGTCACGACTGTCAAGGTGACCTCACCAGCCAACAAGCGGGAAGAGGAACTGGTCAA
PI 251914 GGATGTCACGACTGTCAAGGTGACCTCACCAGCCAACAAGCGGGAAGAGGAACTGGTCAA
PI 115816 GGATGTCACGACTGTCAAGGTGACCTCACCAGCCAACAAGCGGGAAGAGGAACTGGTCAA
PI 61102 GGATGTCACGACTGTCAAGGTGACCTCACCAGCCAACAAGCGGGAAGAGGAACTGGTCAA
PI 283887 GGATGTCACGACTGTCAAGGTGACCTCACCAGCCAACAAGCGGGAAGAGGAACTGGTCAA
PI 94749 GGATGTCACGACTGTCAAGGTGACCTCACCAGCCAACAAGCGGGAAGAGGAACTGGTCAA
PI 115817 GGATGTCACGACTGTCAAGGTGACCTCACCAGCCAACAAGCGGGAAGAGGAACTGGTCAA
****

PI 499972 GGCGCTTGCTGATTCGACAGAAGGGCCGACCCGTTAGCAGGCCGCGGTGTCTTGGGTGCG
CItr 7779 GGCGCTTGCTGATTCGACAGAAGGGCCGACCCGTTAGCAGGCCGCGGTGTCTTGGGTGCG
PI 352281 GGCGCTTGCTGATTCGACAGAAGGGCCGACCCGTTAGCAGGCCGCGGTGTCTTGGGTGCG
PI 286070 GGCGCTTGCTGATTCGACAGAAGGGCCGACCCGTTAGCAGGCCGCGGTGTCTTGGGTGCG
PI 94748 GGCGCTTGCTGATTCGACAGAAGGGCCGACCCGTTAGCAGGCCGCGGTGTCTTGGGTGCG
PS5 GGCGCTTGCTGATTCGACAGAAGGGCCGACCCGTTAGCAGGCCGCGGTGTCTTGGGTGCG
PI 532501 GGCGCTTGCTGATTCGACAGAAGGGCCGACCCGTTAGCAGGCCGCGGTGTCTTGGGTGCG
PI 532475 GGCGCTTGCTGATTCGACAGAAGGGCCGACCCGTTAGCAGGCCGCGGTGTCTTGGGTGCG
PI 470732 GGCGCTTGCTGATTCGACAGAAGGGCCGACCCGTTAGCAGGCCGCGGTGTCTTGGGTGCG

```
PI 349040       GGCGCTTGCTGATTCGACAGAAGGGCCGACCCGTTAGCAGGCCGCGGTGTCTTGGGTGCG
PI 113961       GGCGCTTGCTGATTCGACAGAAGGGCCGACCCGTTAGCAGGCCGCGGTGTCTTGGGTGCG
CItr 3686       GGCGCTTGCTGATTCGACAGAAGGGCCGACCCGTTAGCAGGCCGCGGTGTCTTGGGTGCG
PI 585017       GGCGCTTGCTGATTCGACAGAAGGGCCGACCCGTTAGCAGGCCGCGGTGTCTTGGGTGCG
PI 532486       GGCGCTTGCTGATTCGACAGAAGGGCCGACCCGTTAGCAGGCCGCGGTGTCTTGGGTGCG
PI 352282       GGCGCTTGCTGATTCGACAGAAGGGCCGACCCGTTAGCAGGCCGCGGTGTCTTGGGTGCG
PI 352278       GGCGCTTGCTGATTCGACAGAAGGGCCGACCCGTTAGCAGGCCGCGGTGTCTTGGGTGCG
PI 283889       GGCGCTTGCTGATTCGACAGAAGGGCCGACCCGTTAGCAGGCCGCGGTGTCTTGGGTGCG
PI 251914       GGCGCTTGCTGATTCGACAGAAGGGCCGACCCGTTAGCAGGCCGCGGTGTCTTGGGTGCG
PI 115816       GGCGCTTGCTGATTCGACAGAAGGGCCGACCCGTTAGCAGGCCGCGGTGTCTTGGGTGCG
PI 61102        GGCGCTTGCTGATTCGACAGAAGGGCCGACCCGTTAGCAGGCCGCGGTGTCTTGGGTGCG
PI 283887       GGCGCTTGCTGATTCGACAGAAGGGCCGACCCGTTAGCAGGCCGCGGTGTCTTGGGTGCG
PI 94749        GGCGCTTGCTGATTCGACAGAAGGGCCGACCCGTTAGCAGGCCGCGGTGTCTTGGGTGCG
PI 115817       GGCGCTTGCTGATTCGACAGAAGGGCCGACCCGTTAGCAGGCCGCGGTGTCTTGGGTGCG
                ************************************************************

PI 499972       TGGCCTTGTGGGCCGTATCGAGCCTGCGAGCTCAGGGTTAAGTAAGTGGCTTGGCACCGT
CItr 7779       TGGCCTTGTGGGCCGTATCGAGCCTGCGAGCTCAGGGTTAAGTAAGTGGCTTGGCACCGT
PI 352281       TGGCCTTGTGGGCCGTATCGAGCCTGCGAGCTCAGGGTTAAGTAAGTGGCTTGGCACCGT
PI 286070       TGGCCTTGTGGGCCGTATCGAGCCTGCGAGCTCAGGGTTAAGTAAGTGGCTTGGCACCGT
PI 94748        TGGCCTTGTGGGCCGTATCGAGCCTGCGAGCTCAGGGTTAAGTAAGTGGCTTGGCACCGT
PS5             TGGCCTTGTGGGCCGTATCGAGCCTGCGAGCTCAGGGTTAAGTAAGTGGCTTGGCACCGT
PI 532501       TGGCCTTGTGGGCCGTATCGAGCCTGCGAGCTCAGGGTTAAGTAAGTGGCTTGGCACCGT
PI 532475       TGGCCTTGTGGGCCGTATCGAGCCTGCGAGCTCAGGGTTAAGTAAGTGGCTTGGCACCGT
PI 470732       TGGCCTTGTGGGCCGTATCGAGCCTGCGAGCTCAGGGTTAAGTAAGTGGCTTGGCACCGT
PI 349040       TGGCCTTGTGGGCCGTATCGAGCCTGCGAGCTCAGGGTTAAGTAAGTGGCTTGGCACCGT
PI 113961       TGGCCTTGTGGGCCGTATCGAGCCTGCGAGCTCAGGGTTAAGTAAGTGGCTTGGCACCGT
CItr 3686       TGGCCTTGTGGGCCGTATCGAGCCTGCGAGCTCAGGGTTAAGTAAGTGGCTTGGCACCGT
PI 585017       TGGCCTTGTGGGCCGTATCGAGCCTGCGAGCTCAGGGTTAAGTAAGTGGCTTGGCACCGT
PI 532486       TGGCCTTGTGGGCCGTATCGAGCCTGCGAGCTCAGGGTTAAGTAAGTGGCTTGGCACCGT
PI 352282       TGGCCTTGTGGGCCGTATCGAGCCTGCGAGCTCAGGGTTAAGTAAGTGGCTTGGCACCGT
PI 352278       TGGCCTTGTGGGCCGTATCGAGCCTGCGAGCTCAGGGTTAAGTAAGTGGCTTGGCACCGT
PI 283889       TGGCCTTGTGGGCCGTATCGAGCCTGCGAGCTCAGGGTTAAGTAAGTGGCTTGGCACCGT
PI 251914       TGGCCTTGTGGGCCGTATCGAGCCTGCGAGCTCAGGGTTAAGTAAGTGGCTTGGCACCGT
PI 115816       TGGCCTTGTGGGCCGTATCGAGCCTGCGAGCTCAGGGTTAAGTAAGTGGCTTGGCACCGT
PI 61102        TGGCCTTGTGGGCCGTATCGAGCCTGCGAGCTCAGGGTTAAGTAAGTGGCTTGGCACCGT
PI 283887       TGGCCTTGTGGGCCGTATCGAGCCTGCGAGCTCAGGGTTAAGTAAGTGGCTTGGCACCGT
PI 94749        TGGCCTTGTGGGCCGTATCGAGCCTGCGAGCTCAGGGTTAAGTAAGTGGCTTGGCACCGT
PI 115817       TGGCCTTGTGGGCCGTATCGAGCCTGCGAGCTCAGGGTTAAGTAAGTGGCTTGGCACCGT
                ************************************************************


PI 499972       CAAGGGCATCAAATTGTATCTCACTGAATAGACAGAGAGAAGCTGCTCTTCTCCTGGTTA
CItr 7779       CAAGGGCATCAAATTGTATCTCACTGAATAGACAGAGAGAAGCTGCTCTTCTCCTGGTTA
PI 352281       CAAGGGCATCAAATTGTATCTCACTGAATAGACAGAGAGAAGCTGCTCTTCTCCTGGTTA
PI 286070       CAAGGGCATCAAATTGTATCTCACTGAATAGACAGAGAGAAGCTGCTCTTCTCCTGGTTA
PI 94748        CAAGGGCATCAAATTGTATCTCACTGAATAGACAGAGAGAAGCTGCTCTTCTCCTGGTTA
PS5             CAAGGGCATCAAATTGTATCTCACTGAATAGACAGAGAGAAGCTGCTCTTCTCCTGGTTA
PI 532501       CAAGGGCATCAAATTGTATCTCACTGAATAGACAGAGAGAAGCTGCTCTTCTCCTGGTTA
PI 532475       CAAGGGCATCAAATTGTATCTCACTGAATAGACAGAGAGAAGCTGCTCTTCTCCTGGTTA
PI 470732       CAAGGGCATCAAATTGTATCTCACTGAATAGACAGAGAGAAGCTGCTCTTCTCCTGGTTA
PI 349040       CAAGGGCATCAAATTGTATCTCACTGAATAGACAGAGAGAAGCTGCTCTTCTCCTGGTTA
PI 113961       CAAGGGCATCAAATTGTATCTCACTGAATAGACAGAGAGAAGCTGCTCTTCTCCTGGTTA
CItr 3686       CAAGGGCATCAAATTGTATCTCACTGAATAGACAGAGAGAAGCTGCTCTTCTCCTGGTTA
PI 585017       CAAGGGCATCAAATTGTATCTCACTGAATAGACAGAGAGAAGCTGCTCTTCTCCTGGTTA
PI 532486       CAAGGGCATCAAATTGTATCTCACTGAATAGACAGAGAGAAGCTGCTCTTCTCCTGGTTA
```

```
PI 352282       CAAGGGCATCAAATTGTATCTCACTGAATAGACAGAGAGAAGCTGCTCTTCTCCTGGTTA
PI 352278       CAAGGGCATCAAATTGTATCTCACTGAATAGACAGAGAGAAGCTGCTCTTCTCCTGGTTA
PI 283889       CAAGGGCATCAAATTGTATCTCACTGAATAGACAGAGAGAAGCTGCTCTTCTCCTGGTTA
PI 251914       CAAGGGCATCAAATTGTATCTCACTGAATAGACAGAGAGAAGCTGCTCTTCTCCTGGTTA
PI 115816       CAAGGGCATCAAATTGTATCTCACTGAATAGACAGAGAGAAGCTGCTCTTCTCCTGGTTA
PI 61102        CAAGGGCATCAAATTGTATCTCACTGAATAGACAGAGAGAAGCTGCTCTTCTCCTGGTTA
PI 283887       CAAGGGCATCAAATTGTATCTCACTGAATAGACAGAGAGAAGCTGCTCTTCTCCTGGTTA
PI 94749        CAAGGGCATCAAATTGTATCTCACTGAATAGACAGAGAGAAGCTGCTCTTCTCCTGGTTA
PI 115817       CAAGGGCATCAAATTGTATCTCACTGAATAGACAGAGAGAAGCTGCTCTTCTCCTGGTTA
                ************************************************************

PI 499972       TCCCCTTCCTTCTCCACCTACCTCAATCTCACCTGCCCCCTTCCTCCTCCTCTCAGATCT
CItr 7779       TCCCCTTCCTTCTCCACCTACCTCAATCTCACCTGCCCCCTTCCTCCTCCTCTCAGATCT
PI 352281       TCCCCTTCCTTCTCCACCTACCTCAATCTCACCTGCCCCCTTCCTCCTCCTCTCAGATCT
PI 286070       TCCCCTTCCTTCTCCACCTACCTCAATCTCACCTGCCCCCTTCCTCCTCCTCTCAGATCT
PI 94748        TCCCCTTCCTTCTCCACCTACCTCAATCTCACCTGCCCCCTTCCTCCTCCTCTCAGATCT
PS5             TCCCCTTCCTTCTCCACCTACCTCAATCTCACCTGCCCCCTTCCTCCTCCTCTCAGATCT
PI 532501       TCCCCTTCCTTCTCCACCTACCTCAATCTCACCTGCCCCCTTCCTCCTCCTCTCAGATCT
PI 532475       TCCCCTTCCTTCTCCACCTACCTCAATCTCACCTGCCCCCTTCCTCCTCCTCTCAGATCT
PI 470732       TCCCCTTCCTTCTCCACCTACCTCAATCTCACCTGCCCCCTTCCTCCTCCTCTCAGATCT
PI 349040       TCCCCTTCCTTCTCCACCTACCTCAATCTCACCTGCCCCCTTCCTCCTCCTCTCAGATCT
PI 113961       TCCCCTTCCTTCTCCACCTACCTCAATCTCACCTGCCCCCTTCCTCCTCCTCTCAGATCT
CItr 3686       TCCCCTTCCTTCTCCACCTACCTCAATCTCACCTGCCCCCTTCCTCCTCCTCTCAGATCT
PI 585017       TCCCCTTCCTTCTCCACCTACCTCAATCTCACCTGCCCCCTTCCTCCTCCTCTCAGATCT
PI 532486       TCCCCTTCCTTCTCCACCTACCTCAATCTCACCTGCCCCCTTCCTCCTCCTCTCAGATCT
PI 352282       TCCCCTTCCTTCTCCACCTACCTCAATCTCACCTGCCCCCTTCCTCCTCCTCTCAGATCT
PI 352278       TCCCCTTCCTTCTCCACCTACCTCAATCTCACCTGCCCCCTTCCTCCTCCTCTCAGATCT
PI 283889       TCCCCTTCCTTCTCCACCTACCTCAATCTCACCTGCCCCCTTCCTCCTCCTCTCAGATCT
PI 251914       TCCCCTTCCTTCTCCACCTACCTCAATCTCACCTGCCCCCTTCCTCCTCCTCTCAGATCT
PI 115816       TCCCCTTCCTTCTCCACCTACCTCAATCTCACCTGCCCCCTTCCTCCTCCTCTCAGATCT
PI 61102        TCCCCTTCCTTCTCCACCTACCTCAATCTCACCTGCCCCCTTCCTCCTCCTCTCAGATCT
PI 283887       TCCCCTTCCTTCTCCACCTACCTCAATCTCACCTGCCCCCTTCCTCCTCCTCTCAGATCT
PI 94749        TCCCCTTCCTTCTCCACCTACCTCAATCTCACCTGCCCCCTTCCTCCTCCTCTCAGATCT
PI 115817       TCCCCTTCCTTCTCCACCTACCTCAATCTCACCTGCCCCCTTCCTCCTCCTCTCAGATCT
                ************************************************************


PI 499972       GATCGATTAGGTAGATCGGGCCGTTACACTCCGCCCCCCCGCCCCCGCCTGCGCCTGTCG
CItr 7779       GATCGATTAGGTAGATCGGGCCGTTACACTCCGCCCCCCCGCCCCCGCCTGCGCCTGTCG
PI 352281       GATCGATTAGGTAGATCGGGCCGTTACACTCCGCCCCCCCGCCCCCGCCTGCGCCTGTCG
PI 286070       GATCGATTAGGTAGATCGGGCCGTTACACTCCGCCCCCCCGCCCCCGCCTGCGCCTGTCG
PI 94748        GATCGATTAGGTAGATCGGGCCGTTACACTCCGCCCCCCCGCCCCCGCCTGCGCCTGTCG
PS5             GATCGATTAGGTAGATCGGGCCGTTACACTCCGCCCCCCCGCCCCCGCCTGCGCCTGTCG
PI 532501       GATCGATTAGGTAGATCGGGCCGTTACACTCCGCCCCCCCGCCCCCGCCTGCGCCTGTCG
PI 532475       GATCGATTAGGTAGATCGGGCCGTTACACTCCGCCCCCCCGCCCCCGCCTGCGCCTGTCG
PI 470732       GATCGATTAGGTAGATCGGGCCGTTACACTCCGCCCCCCCGCCCCCGCCTGCGCCTGTCG
PI 349040       GATCGATTAGGTAGATCGGGCCGTTACACTCCGCCCCCCCGCCCCCGCCTGCGCCTGTCG
PI 113961       GATCGATTAGGTAGATCGGGCCGTTACACTCCGCCCCCCCGCCCCCGCCTGCGCCTGTCG
CItr 3686       GATCGATTAGGTAGATCGGGCCGTTACACTCCGCCCCCCCGCCCCCGCCTGCGCCTGTCG
PI 585017       GATCGATTAGGTAGATCGGGCCGTTACACTCCGCCCCCCCGCCCCCGCCTGCGCCTGTCG
PI 532486       GATCGATTAGGTAGATCGGGCCGTTACACTCCGCCCCCCCGCCCCCGCCTGCGCCTGTCG
PI 352282       GATCGATTAGGTAGATCGGGCCGTTACACTCCGCCCCCCCGCCCCCGCCTGCGCCTGTCG
PI 352278       GATCGATTAGGTAGATCGGGCCGTTACACTCCGCCCCCCCGCCCCCGCCTGCGCCTGTCG
PI 283889       GATCGATTAGGTAGATCGGGCCGTTACACTCCGCCCCCCCGCCCCCGCCTGCGCCTGTCG
PI 251914       GATCGATTAGGTAGATCGGGCCGTTACACTCCGCCCCCCCGCCCCCGCCTGCGCCTGTCG
PI 115816       GATCGATTAGGTAGATCGGGCCGTTACACTCCGCCCCCCCGCCCCCGCCTGCGCCTGTCG
```

```
PI 61102        GATCGATTAGGTAGATCGGGCCGTTACACTCCGCCCCCCCGCCCCCGCCTGCGCCTGTCG
PI 283887       GATCGATTAGGTAGATCGGGCCGTTACACTCCGCCCCCCCGCCCCCGCCTGCGCCTGTCG
PI 94749        GATCGATTAGGTAGATCGGGCCGTTACACTCCGCCCCCCCGCCCCCGCCTGCGCCTGTCG
PI 115817       GATCGATTAGGTAGATCGGGCCGTTACACTCCGCCCCCCCGCCCCCGCCTGCGCCTGTCG
                ************************************************************

PI 499972       GAGTAGCCGTCGCGGTCTGCCGGTGTTGGAGGCTTGGGGTGTAGGGTTGGCCCCGTTCTC
CItr 7779       GAGTAGCCGTCGCGGTCTGCCGGTGTTGGAGGCTTGGGGTGTAGGGTTGGCCCCGTTCTC
PI 352281       GAGTAGCCGTCGCGGTCTGCCGGTGTTGGAGGCTTGGGGTGTAGGGTTGGCCCCGTTCTC
PI 286070       GAGTAGCCGTCGCGGTCTGCCGGTGTTGGAGGCTTGGGGTGTAGGGTTGGCCCCGTTCTC
PI 94748        GAGTAGCCGTCGCGGTCTGCCGGTGTTGGAGGCTTGGGGTGTAGGGTTGGCCCCGTTCTC
PS5             GAGTAGCCGTCGCGGTCTGCCGGTGTTGGAGGCTTGGGGTGTAGGGTTGGCCCCGTTCTC
PI 532501       GAGTAGCCGTCGCGGTCTGCCGGTGTTGGAGGCTTGGGGTGTAGGGTTGGCCCCGTTCTC
PI 532475       GAGTAGCCGTCGCGGTCTGCCGGTGTTGGAGGCTTGGGGTGTAGGGTTGGCCCCGTTCTC
PI 470732       GAGTAGCCGTCGCGGTCTGCCGGTGTTGGAGGCTTGGGGTGTAGGGTTGGCCCCGTTCTC
PI 349040       GAGTAGCCGTCGCGGTCTGCCGGTGTTGGAGGCTTGGGGTGTAGGGTTGGCCCCGTTCTC
PI 113961       GAGTAGCCGTCGCGGTCTGCCGGTGTTGGAGGCTTGGGGTGTAGGGTTGGCCCCGTTCTC
CItr 3686       GAGTAGCCGTCGCGGTCTGCCGGTGTTGGAGGCTTGGGGTGTAGGGTTGGCCCCGTTCTC
PI 585017       GAGTAGCCGTCGCGGTCTGCCGGTGTTGGAGGCTTGGGGTGTAGGGTTGGCCCCGTTCTC
PI 532486       GAGTAGCCGTCGCGGTCTGCCGGTGTTGGAGGCTTGGGGTGTAGGGTTGGCCCCGTTCTC
PI 352282       GAGTAGCCGTCGCGGTCTGCCGGTGTTGGAGGCTTGGGGTGTAGGGTTGGCCCCGTTCTC
PI 352278       GAGTAGCCGTCGCGGTCTGCCGGTGTTGGAGGCTTGGGGTGTAGGGTTGGCCCCGTTCTC
PI 283889       GAGTAGCCGTCGCGGTCTGCCGGTGTTGGAGGCTTGGGGTGTAGGGTTGGCCCCGTTCTC
PI 251914       GAGTAGCCGTCGCGGTCTGCCGGTGTTGGAGGCTTGGGGTGTAGGGTTGGCCCCGTTCTC
PI 115816       GAGTAGCCGTCGCGGTCTGCCGGTGTTGGAGGCTTGGGGTGTAGGGTTGGCCCCGTTCTC
PI 61102        GAGTAGCCGTCGCGGTCTGCCGGTGTTGGAGGCTTGGGGTGTAGGGTTGGCCCCGTTCTC
PI 283887       GAGTAGCCGTCGCGGTCTGCCGGTGTTGGAGGCTTGGGGTGTAGGGTTGGCCCCGTTCTC
PI 94749        GAGTAGCCGTCGCGGTCTGCCGGTGTTGGAGGCTTGGGGTGTAGGGTTGGCCCCGTTCTC
PI 115817       GAGTAGCCGTCGCGGTCTGCCGGTGTTGGAGGCTTGGGGTGTAGGGTTGGCCCCGTTCTC
                ************************************************************

PI 499972       CAGCGGAGATGGGGC
CItr 7779       CAGCGGAGATGGGGC
PI 352281       CAGCGGAGATGGGGC
PI 286070       CAGCGGAGATGGGGC
PI 94748        CAGCGGAGATGGGGC
PS5             CAGCGGAGATGGGGC
PI 532501       CAGCGGAGATGGGGC
PI 532475       CAGCGGAGATGGGGC
PI 470732       CAGCGGAGATGGGGC
PI 349040       CAGCGGAGATGGGGC
PI 113961       CAGCGGAGATGGGGC
CItr 3686       CAGCGGAGATGGGGC
PI 585017       CAGCGGAGATGGGGC
PI 532486       CAGCGGAGATGGGGC
PI 352282       CAGCGGAGATGGGGC
PI 352278       CAGCGGAGATGGGGC
PI 283889       CAGCGGAGATGGGGC
PI 251914       CAGCGGAGATGGGGC
PI 115816       CAGCGGAGATGGGGC
PI 61102        CAGCGGAGATGGGGC
PI 283887       CAGCGGAGATGGGGC
PI 94749        CAGCGGAGATGGGGC
PI 115817       CAGCGGAGATGGGGC
                ***************
```

Figure S7 Alignment of the sequences contains 5'- and 3'- ends of retrotrans_VRN in PI 94749 and other 22 tetraploid wheat accessions. The two CTCCG motifs are shown in red and highlighted in yellow.

Table S1 Winter/spring growth habit in the 146 double haploid (DH) lines derived from the cross between durum wheat 'Lebsock' and *T. turgidum* subsp. *carthlicum* accession PI 94749 [a]

Spring DH lines with *Vrn-A1* and *Vrn-B1* alleles (31 lines):

LP749-6	LP749-7	LP749-13	LP749-16	LP749-22	LP749-27	LP749-29	LP749-37
LP749-42	LP749-49	LP749-52	LP749-55	LP749-57	LP749-64	LP749-66	LP749-68
LP749-71	LP749-80	LP749-81	LP749-85	LP749-87	LP749-89	LP749-90	LP749-92
LP749-93	LP749-96	LP749-107	LP749-111	LP749-117	LP749-122	LP749-138	

Spring DH lines with *Vrn-A1* and *vrn-B1* alleles (33 lines):

LP749-4	LP749-8	LP749-11	LP749-14	LP749-15	LP749-17	LP749-19	LP749-21
LP749-32	LP749-33	LP749-39	LP749-41	LP749-44	LP749-50	LP749-60	LP749-62
LP749-63	LP749-67	LP749-70	LP749-72	LP749-82	LP749-83	LP749-84	LP749-97
LP749-109	LP749-113	LP749-114	LP749-120	LP749-126	LP749-130	LP749-139	LP749-140
LP749-146							

Spring DH lines with *vrn-A1* and *Vrn-B1* alleles (37 lines):

LP749-2	LP749-5	LP749-10	LP749-18	LP749-20	LP749-23	LP749-35	LP749-40
LP749-43	LP749-47	LP749-54	LP749-56	LP749-58	LP749-59	LP749-65	LP749-73
LP749-74	LP749-75	LP749-76	LP749-78	LP749-86	LP749-88	LP749-91	LP749-102
LP749-103	LP749-104	LP749-108	LP749-110	LP749-116	LP749-118	LP749-123	LP749-124
LP749-125	LP749-133	LP749-137	LP749-141	LP749-145			

Winter DH lines with *vrn-A1* and *vrn-B1* alleles (45 lines):

LP749-1	LP749-3	LP749-9	LP749-12	LP749-24	LP749-25	LP749-26	LP749-28
LP749-30	LP749-31	LP749-34	LP749-36	LP749-38	LP749-45	LP749-46	LP749-48
LP749-51	LP749-53	LP749-61	LP749-69	LP749-77	LP749-79	LP749-94	LP749-95
LP749-98	LP749-99	LP749-100	LP749-101	LP749-105	LP749-106	LP749-112	LP749-115
LP749-119	LP749-121	LP749-127	LP749-128	LP749-129	LP749-131	LP749-132	LP749-134
LP749-135	LP749-136	LP749-142	LP749-143	LP749-144			

[a] *VRN*-1 alleles carried by each line were verified through gene specific marker developed in this study.

Table S2 Frequency of *Vrn-B1* allele containing retrotrans_VRN in 154 spring type accessions or lines from six tetraploid wheat (*Triticum turgidum* L.) sub-species.

Accession no. or line [a]	Origin [b]	Sub-species	PCR amplification [c] VRNBPF1/VRNBPR1	PCR amplification [c] VRNBPF1/INSR1	Containing retrotrans_VRN [d]
CItr 7665	Russian Federation	*T. turgidum* subsp. *carthlicum*	1	0	-
PI 182471	Turkey	*T. turgidum* subsp. *carthlicum*	1	0	-
PI 341800	Russian Federation	*T. turgidum* subsp. *carthlicum*	1	0	-
PI 61102	Georgia	*T. turgidum* subsp. *carthlicum*	0	1	+
PI 94748	Georgia	*T. turgidum* subsp. *carthlicum*	0	1	+
PI 115816	Georgia	*T. turgidum* subsp. *carthlicum*	0	1	+
PI 115817	Georgia	*T. turgidum* subsp. *carthlicum*	0	1	+
PI 251914	Georgia	*T. turgidum* subsp. *carthlicum*	0	1	+
PI 283887	Iran	*T. turgidum* subsp. *carthlicum*	0	1	+
PI 283889	Iran	*T. turgidum* subsp. *carthlicum*	0	1	+
PI 286070	Poland	*T. turgidum* subsp. *carthlicum*	0	1	+
PI 349040	Armenia	*T. turgidum* subsp. *carthlicum*	0	1	+
PI 352278	Georgia	*T. turgidum* subsp. *carthlicum*	0	1	+
PI 352281	Caucasus region in Former Soviet Union	*T. turgidum* subsp. *carthlicum*	0	1	+
PI 352282	Georgia	*T. turgidum* subsp. *carthlicum*	0	1	+
PI 470732	Turkey	*T. turgidum* subsp. *carthlicum*	0	1	+
PI 499972	Georgia	*T. turgidum* subsp. *carthlicum*	0	1	+
PI 532475	Turkey	*T. turgidum* subsp. *carthlicum*	0	1	+
PI 532486	Turkey	*T. turgidum* subsp. *carthlicum*	0	1	+
PI 532501	Former Soviet Union	*T. turgidum* subsp. *carthlicum*	0	1	+
PI 585017	Georgia	*T. turgidum* subsp. *carthlicum*	0	1	+
PS5	China	*T. turgidum* subsp. *carthlicum*	0	1	+
CItr 3686	MN, USA	*T. turgidum* subsp. *dicoccum*	0	1	+
CItr 7779	Ethiopia	*T. turgidum* subsp. *dicoccum*	0	1	+
PI 113961	Georgia	*T. turgidum* subsp. *dicoccum*	0	1	+
CItr 14133	AZ, USA	*T. turgidum* subsp. *dicoccum*	1	0	-
CItr 4013	India	*T. turgidum* subsp. *dicoccum*	1	0	-
CItr 7685	Russian Federation	*T. turgidum* subsp. *dicoccum*	1	0	-
CItr 7962	Ethiopia	*T. turgidum* subsp. *dicoccum*	1	0	-
CItr 12214	India	*T. turgidum* subsp. *dicoccum*	1	0	-
CItr 14098	Ethiopia	*T. turgidum* subsp. *dicoccum*	1	0	-
CItr 14454	Ethiopia	*T. turgidum* subsp. *dicoccum*	1	0	-

CItr 14592	Ethiopia	*T. turgidum* subsp. *dicoccum*	1	0	-
CItr 14621	Ethiopia	*T. turgidum* subsp. *dicoccum*	1	0	-
CItr 14636	Ethiopia	*T. turgidum* subsp. *dicoccum*	1	0	-
CItr 14751	Ethiopia	*T. turgidum* subsp. *dicoccum*	1	0	-
CItr 14822	Eritrea	*T. turgidum* subsp. *dicoccum*	1	0	-
CItr 14868	Ethiopia	*T. turgidum* subsp. *dicoccum*	1	0	-
CItr 14919	Unknown	*T. turgidum* subsp. *dicoccum*	1	0	-
CItr 14970	Unknown	*T. turgidum* subsp. *dicoccum*	1	0	-
PI 101971	India	*T. turgidum* subsp. *dicoccum*	1	0	-
PI 133134	Peru	*T. turgidum* subsp. *dicoccum*	1	0	-
PI 154582	China	*T. turgidum* subsp. *dicoccum*	1	0	-
PI 164578	India	*T. turgidum* subsp. *dicoccum*	1	0	-
PI 168673	WI, USA	*T. turgidum* subsp. *dicoccum*	1	0	-
PI 190926	Belgium	*T. turgidum* subsp. *dicoccum*	1	0	-
PI 191091	Spain	*T. turgidum* subsp. *dicoccum*	1	0	-
PI 191387	Ethiopia	*T. turgidum* subsp. *dicoccum*	1	0	-
PI 193641	Ethiopia	*T. turgidum* subsp. *dicoccum*	1	0	-
PI 193873	Ethiopia	*T. turgidum* subsp. *dicoccum*	1	0	-
PI 193878	Ethiopia	*T. turgidum* subsp. *dicoccum*	1	0	-
PI 194041	Ethiopia	*T. turgidum* subsp. *dicoccum*	1	0	-
Alkabo	ND, USA	*T. turgidum* subsp. *durum*	1	0	-
Ben	ND, USA	*T. turgidum* subsp. *durum*	1	0	-
Golden Ball	South Africa	*T. turgidum* subsp. *durum*	1	0	-
Mountrail	ND, USA	*T. turgidum* subsp. *durum*	1	0	-
Nora	ND, USA	*T. turgidum* subsp. *durum*	1	0	-
Parshall	ND, USA	*T. turgidum* subsp. *durum*	1	0	-
Renville	ND, USA	*T. turgidum* subsp. *durum*	1	0	-
Rugby	ND, USA	*T. turgidum* subsp. *durum*	1	0	-
Wells	ND, USA	*T. turgidum* subsp. *durum*	1	0	-
TA 4154-1	CIMMYT	*T. turgidum* subsp. *durum*	1	0	-
TA 4154-2	CIMMYT	*T. turgidum* subsp. *durum*	1	0	-
TA 4154-3	CIMMYT	*T. turgidum* subsp. *durum*	1	0	-
TA 4154-4	CIMMYT	*T. turgidum* subsp. *durum*	1	0	-
TA 4154-5	CIMMYT	*T. turgidum* subsp. *durum*	1	0	-
TA 4154-6	CIMMYT	*T. turgidum* subsp. *durum*	1	0	-
TA 4154-7	CIMMYT	*T. turgidum* subsp. *durum*	1	0	-
TA 4154-8	CIMMYT	*T. turgidum* subsp. *durum*	1	0	-
TA 4154-9	CIMMYT	*T. turgidum* subsp. *durum*	1	0	-
TA 4154-10	CIMMYT	*T. turgidum* subsp. *durum*	1	0	-

TA 4154-11	CIMMYT	*T. turgidum* subsp. *durum*	1	0	-
TA 4154-12	CIMMYT	*T. turgidum* subsp. *durum*	1	0	-
TA 4154-13	CIMMYT	*T. turgidum* subsp. *durum*	1	0	-
TA 4154-14	CIMMYT	*T. turgidum* subsp. *durum*	1	0	-
TA 4154-15	CIMMYT	*T. turgidum* subsp. *durum*	1	0	-
TA 4154-16	CIMMYT	*T. turgidum* subsp. *durum*	1	0	-
TA 4154-17	CIMMYT	*T. turgidum* subsp. *durum*	1	0	-
TA 4154-18	CIMMYT	*T. turgidum* subsp. *durum*	1	0	-
TA 4154-19	CIMMYT	*T. turgidum* subsp. *durum*	1	0	-
TA 4154-20	CIMMYT	*T. turgidum* subsp. *durum*	1	0	-
TA 4154-21	CIMMYT	*T. turgidum* subsp. *durum*	1	0	-
TA 4154-22	CIMMYT	*T. turgidum* subsp. *durum*	1	0	-
TA 4154-23	CIMMYT	*T. turgidum* subsp. *durum*	1	0	-
TA 4154-25	CIMMYT	*T. turgidum* subsp. *durum*	1	0	-
PI 42209	Australia	*T. turgidum* subsp. *polonicum*	1	0	-
PI 56261	Portugal	*T. turgidum* subsp. *polonicum*	1	0	-
PI 56262	Portugal	*T. turgidum* subsp. *polonicum*	1	0	-
PI 167622	Turkey	*T. turgidum* subsp. *polonicum*	1	0	-
PI 190951	Portugal	*T. turgidum* subsp. *polonicum*	1	0	-
PI 191620	Portugal	*T. turgidum* subsp. *polonicum*	1	0	-
CItr 14139	Unknown	*T. turgidum* subsp. *polonicum*	1	0	-
CItr 17442	CA, USA	*T. turgidum* subsp. *polonicum*	1	0	-
PI 192666	Portugal	*T. turgidum* subsp. *polonicum*	1	0	-
PI 208911	Iraq	*T. turgidum* subsp. *polonicum*	1	0	-
PI 210845	Iran	*T. turgidum* subsp. *polonicum*	1	0	-
PI 223171	Jordan	*T. turgidum* subsp. *polonicum*	1	0	-
PI 266846	England, UK	*T. turgidum* subsp. *polonicum*	1	0	-
PI 272564	Hungary	*T. turgidum* subsp. *polonicum*	1	0	-
PI 272570	Hungary	*T. turgidum* subsp. *polonicum*	1	0	-
PI 286547	Ecuador	*T. turgidum* subsp. *polonicum*	1	0	-
PI 290512	Portugal	*T. turgidum* subsp. *polonicum*	1	0	-
PI 306548	Romania	*T. turgidum* subsp. *polonicum*	1	0	-
PI 330554	England, UK	*T. turgidum* subsp. *polonicum*	1	0	-
PI 330555	England, UK	*T. turgidum* subsp. *polonicum*	1	0	-
CItr 7809	Ethiopia	*T. turgidum* subsp. *turgidum*	1	0	-
CItr 7839	Ethiopia	*T. turgidum* subsp. *turgidum*	1	0	-
CItr 7859	Ethiopia	*T. turgidum* subsp. *turgidum*	1	0	-
CItr 13712	OR, USA	*T. turgidum* subsp. *turgidum*	1	0	-
PI 32039	China	*T. turgidum* subsp. *turgidum*	1	0	-

PI 41029	Georgia	*T. turgidum* subsp. *turgidum*	1	0	-
PI 60617	Ethiopia	*T. turgidum* subsp. *turgidum*	1	0	-
PI 60729	Egypt	*T. turgidum* subsp. *turgidum*	1	0	-
PI 67339	Australia	*T. turgidum* subsp. *turgidum*	1	0	-
PI 94689	Armenia	*T. turgidum* subsp. *turgidum*	1	0	-
PI 134947	Portugal	*T. turgidum* subsp. *turgidum*	1	0	-
PI 134948	Portugal	*T. turgidum* subsp. *turgidum*	1	0	-
PI 134951	Portugal	*T. turgidum* subsp. *turgidum*	1	0	-
PI 134953	Portugal	*T. turgidum* subsp. *turgidum*	1	0	-
PI 134954	Portugal	*T. turgidum* subsp. *turgidum*	1	0	-
PI 134956	Portugal	*T. turgidum* subsp. *turgidum*	1	0	-
PI 134957	Portugal	*T. turgidum* subsp. *turgidum*	1	0	-
PI 134959	Portugal	*T. turgidum* subsp. *turgidum*	1	0	-
PI 134962	Portugal	*T. turgidum* subsp. *turgidum*	1	0	-
PI 254206	Iran	*T. turgidum* subsp. *turanicum*	1	0	-
PI 254208	Iran	*T. turgidum* subsp. *turanicum*	1	0	-
PI 254212	Iran	*T. turgidum* subsp. *turanicum*	1	0	-
PI 254213	Turkey	*T. turgidum* subsp. *turanicum*	1	0	-
PI 256034	Spain	*T. turgidum* subsp. *turanicum*	1	0	-
PI 272601	Hungary	*T. turgidum* subsp. *turanicum*	1	0	-
PI 272602	Hungary	*T. turgidum* subsp. *turanicum*	1	0	-
PI 278350	Italy	*T. turgidum* subsp. *turanicum*	1	0	-
PI 283795	Afghanistan	*T. turgidum* subsp. *turanicum*	1	0	-
PI 286069	Poland	*T. turgidum* subsp. *turanicum*	1	0	-
PI 290530	Hungary	*T. turgidum* subsp. *turanicum*	1	0	-
PI 306665	France	*T. turgidum* subsp. *turanicum*	1	0	-
PI 317495	Afghanistan	*T. turgidum* subsp. *turanicum*	1	0	-
PI 321737	Afghanistan	*T. turgidum* subsp. *turanicum*	1	0	-
PI 337643	Afghanistan	*T. turgidum* subsp. *turanicum*	1	0	-
PI 347132	Afghanistan	*T. turgidum* subsp. *turanicum*	1	0	-
PI 349055	Russian Federation	*T. turgidum* subsp. *turanicum*	1	0	-
PI 352514	Azerbaijan	*T. turgidum* subsp. *turanicum*	1	0	-
PI 352515	Iran	*T. turgidum* subsp. *turanicum*	1	0	-
PI 362067	Romania	*T. turgidum* subsp. *turanicum*	1	0	-
PI 481582	Iraq	*T. turgidum* subsp. *turanicum*	1	0	-
PI 532136	Egypt	*T. turgidum* subsp. *turanicum*	1	0	-
PI 537992	Yemen	*T. turgidum* subsp. *turanicum*	1	0	-
PI 559976	Morocco	*T. turgidum* subsp. *turanicum*	1	0	-
PI 560896	Turkey	*T. turgidum* subsp. *turanicum*	1	0	-

PI 623629	Iran	*T. turgidum* subsp. *turanicum*	1	0	-
PI 624208	Iran	*T. turgidum* subsp. *turanicum*	1	0	-
PI 624217	Iran	*T. turgidum* subsp. *turanicum*	1	0	-
PI 624421	Iran	*T. turgidum* subsp. *turanicum*	1	0	-
PI 624422	Iran	*T. turgidum* subsp. *turanicum*	1	0	-

[a] TA and PI/CItr are the accession numbers in Wheat Genetic and Genomic Resource Center (WGGRC) at Kansas State University in Manhattan, Kansas and USDA National Small Grains Collection, Aberdeen, Idaho, respectively.

[b] Origin of the accessions was obtained through USDA National Plant Germplasm System (NPGS) (http://www.ars-grin.gov/npgs/). Durum lines with TA number were originally obtained from International Maize and Wheat Improvement Center (CIMMYT), Mexico.

[c] Two primer pairs VRNBPF1/ VRNBPR1 and VRNBPF1/ INSR1 can detect the absence and presence of retrotrans_VRN in *VRN-B1*, respectively. For PCR amplification, 1 and 0 means the presence and absence of the specific band, respectively.

[d] ‘+’ and ‘-‘ represents presence and absence, respectively.

Table S3 The growth habit and days to heading of the DH lines that were used in the expression experiment for *VRN-1* genes.

Line	Genotype	Growth habit	No. of plants used		Days to heading (days)	
			Unvernalized	vernalized	Unvernalized	Vernalized
Lebsock	*Vrn-A1Vrn-A1 vrn-B1vrn-B1*	Spring	5	-	51.6	-
PI 94749	*vrn-A1vrn-A1 Vrn-B1Vrn-B1*	Spring	5	-	67	-
LP749-7	*Vrn-A1Vrn-A1 Vrn-B1Vrn-B1*	Spring	5	-	54	-
LP749-16	*Vrn-A1Vrn-A1 Vrn-B1Vrn-B1*	Spring	5	-	47	-
LP749-71	*Vrn-A1Vrn-A1 Vrn-B1Vrn-B1*	Spring	4	-	50	-
LP749-80	*Vrn-A1Vrn-A1 Vrn-B1Vrn-B1*	Spring	5	-	49.2	-
LP749-8	*Vrn-A1Vrn-A1 vrn-B1vrn-B1*	Spring	5	-	54.4	-
LP749-11	*Vrn-A1Vrn-A1 vrn-B1vrn-B1*	Spring	4	-	52.5	-
LP749-82	*Vrn-A1Vrn-A1 vrn-B1vrn-B1*	Spring	5	-	56	-
LP749-97	*Vrn-A1Vrn-A1 vrn-B1vrn-B1*	Spring	5	-	56.4	-
LP749-10	*vrn-A1vrn-A1 Vrn-B1Vrn-B1*	Spring	4	-	50.3	-
LP749-18	*vrn-A1vrn-A1 Vrn-B1Vrn-B1*	Spring	5	-	55.4	-
LP749-20	*vrn-A1vrn-A1 Vrn-B1Vrn-B1*	Spring	4	-	52.3	-
LP749-43	*vrn-A1vrn-A1 Vrn-B1Vrn-B1*	Spring	5	-	56.6	-
LP749-54	*vrn-A1vrn-A1 Vrn-B1Vrn-B1*	Spring	5	-	58.8	-
LP749-58	*vrn-A1vrn-A1 Vrn-B1Vrn-B1*	Spring	5	-	55.4	-
LP749-65	*vrn-A1vrn-A1 Vrn-B1Vrn-B1*	Spring	4	-	47.8	-

LP749-88	*vrn-A1vrn-A1 Vrn-B1Vrn-B1*	Spring	5	-	49.4	-
LP749-25	*vrn-A1vrn-A1 vrn-B1vrn-B1*	Winter	5	-	92.6	-
LP749-28	*vrn-A1vrn-A1 vrn-B1vrn-B1*	Winter	5	-	105	-
LP749-30	*vrn-A1vrn-A1 vrn-B1vrn-B1*	Winter	5	-	145	-
LP749-34	*vrn-A1vrn-A1 vrn-B1vrn-B1*	Winter	3	-	97	-
LP749-53	*vrn-A1vrn-A1 vrn-B1vrn-B1*	Winter	4	-	111.3	-
LP749-36	*vrn-A1vrn-A1 vrn-B1vrn-B1*	Winter	5	5	145	112.8
LP749-61	*vrn-A1vrn-A1 vrn-B1vrn-B1*	Winter	5	5	145	106.8
