# Supplementary material for: A Novel Retrotransposon Inserted in the Dominant Vrn-B1 Allele Confers Spring Growth Habit in Tetraploid Wheat (Triticum turgidum L.)
Source: G3 (Bethesda). 2011 Dec 1;1(7):637–45. doi: 10.1534/g3.111.001131 (PMC3276170; doi:10.1534/g3.111.001131)
Supplement: Supporting Information [file supp_1.7.637_FigureS1.pdf]

```
PI  GAAAGGAAAAATTCTGCTCGTTTTTTTTTCTCTGTGGTGTGTGTTTGTGGCGAGAGAAAAT
LB  GAAAGGAAAAATTCTGCTCGTTTTTTTTTCTCTGTGGTGTGTGTTTGTGGCGAGAGAAAAT
    ************************************************************

PI  GATTTGGGGAAAGCAAAATCCGGAGATTCGCACGTACGATCGTTCGACACGTCGACGCCC
LB  GATTTGGGGAAAGCAAAATCCGGAGATTCGCACGTACGATCGTTCGACACGTCGACGCCC
    ************************************************************

PI  GGCGGGCCCGGGGTGGGGCATCGTGTGGCTGCAGGACCGCGGGGCCCCGCAAAGCGGGCC
LB  GGCGGGCCCGGGGTGGGGCATCGTGTGGCTGCAGGACCGCGGGGCCCCGCAAAGCGGGCC
    ************************************************************

PI  GGGCCAATGGGTGCTCGACAGCGGCTATGCTCCAGACCAGCCCGGTATTGCATACCGCGC
LB  GGGCCAATGGGTGCTCGACAGCGGCTATGCTCCAGACCAGCCCGGTATTGCATACCGCGC
    ************************************************************

PI  TCGGGGCCAGATCCCTTTAAAAACCCCTCCCCCCCTGCCGGAATCCTCGTTTTGGCCTGG
LB  TCGGGGCCAGATCCCTTTAAAAACCCCTCCCCCCCTGCCGGAATCCTCGTTTTGGCCTGG
    ************************************************************

PI  CCATCCTCCCTCTCCTCCCCTCTCTTCCACCTCACGTCCTCACCCAACCACCTGATAGCC
LB  CCATCCTCCCTCTCCTCCCCTCTCTTCCACCTCACGTCCTCACCCAACCACCTGATAGCC
    ************************************************************

PI  ATGGCTCCGCCGCCTCGCCTCCGCCTGCGCCAGTCGGAGTAGCCGTCGCGGTCTGCCGGT
LB  ATGGCTCCGCCGCCTCGCCTCCGCCTGCGCCAGTCGGAGTAGCCGTCGCGGTCTGCCGGT
    ************************************************************

PI  GTTGGAGGGTAGGGGCGTAGGGTTGGCCCGGTTCTCGAGCGGAGATGGGGCGGGGGAAGG
LB  GTTGGAGGGTAGGGGCGTAGGGTTGGCCCGGTTCTCGAGCGGAGATGGGGCGGGGGAAGG
    ************************************************************

PI  TGCA
LB  TGCA
    ****
```

**Figure S1** DNA sequence comparison indicates the same 484-bp fragment amplified by primer pair VRN1AF/VRN1AR in both Lebsock (LB) and PI 94749 (PI), which indicated no sequence variation in the promoter region of the *VRN-A1* gene of the two parental lines.
