# Supplementary material for: A Novel Retrotransposon Inserted in the Dominant Vrn-B1 Allele Confers Spring Growth Habit in Tetraploid Wheat (Triticum turgidum L.)
Source: G3 (Bethesda). 2011 Dec 1;1(7):637–45. doi: 10.1534/g3.111.001131 (PMC3276170; doi:10.1534/g3.111.001131)
Supplement: Supporting Information [file supp_1.7.637_FigureS2.pdf]

```
PI  CAAGTGGAACGGTTAGGACAGTAATCTCTTGATATTTTTATCTGGCTGGGGATATTTACGTAAAAAAATTAT
LB  CAAGTGGAACGGTTAGGACAGTAATCTCTTGATATTTTTATCTGGCTGGGGATATTTACGTAAAAAAATTAT
    ************************************************************************

PI  ATGGGGTTAAAGTGACATCGCAATTTAGCATGCTACCTCATCTTCTCATTTAGAATCTTACTAGACGCTACA
LB  ATGGGGTTAAAGTGACATCGCAATTTAGCATGCTACCTCATCTTCTCATTTAGAATCTTACTAGACGCTACA
    ************************************************************************

PI  ATACCTTGTTGTCTGGCTCATCAAATCTGTGCTTGCTGCTTGAACAAATGAACCTCGTCATCTCGGTTATTT
LB  ATACCTTGTTGTCTGGCTCATCAAATCTGTGCTTGCTGCTTGAACAAATGAACCTCGTCATCTCGGTTATTT
    ************************************************************************

PI  CCAGAATTTTGTTCCACAGGCTTCCCTATCATTCGTATTGCTAGCTCCGGCCATGCGGCCATTTTGTTGCTT
LB  CCAGAATTTTGTTCCACAGGCTTCCCTATCATTCGTATTGCTAGCTCCGGCCATGCGGCCATTTTGTTGCTT
    ************************************************************************

PI  GCCTGGAGATACTGTCTACGGCACGCACGGAGAAAAGAGTCACTTGACTAGCTAATGCATGGAATAATTGTC
LB  GCCTGGAGATACTGTCTACGGCACGCACGGAGAAAAGAGTCACTTGACTAGCTAATGCATGGAATAATTGTC
    ************************************************************************

PI  TGCAGCTGATGAAACTCCGGCATGAAGAGTCAAACCAAAAAGTAGAGAGTTCCTTCCAAATATAAAATGAGA
LB  TGCAGCTGATGAAACTCCGGCATGAAGAGTCAAACCAAAAAGTAGAGAGTTCCTTCCAAATATAAAATGAGA
    ************************************************************************

PI  GTTTCTGCAGACTTTTTCCCTTTCAACCATCATAATTTGCCTGTGATATTTGTTGGTGCTGGCGATGGTTCT
LB  GTTTCTGCAGACTTTTTCCCTTTCAACCATCATAATTTGCCTGTGATATTTGTTGGTGCTGGCGATGGTTCT
    ************************************************************************

PI  TGACAAAGTAAAGGAGTCAATAAAATCACGGAGACTGATCCATTCTTTCCCCCACACGCTGACATTAGTCCA
LB  TGACAAAGTAAAGGAGTCAATAAAATCACGGAGACTGATCCATTCTTTCCCCCACACGCTGACATTAGTCCA
    ************************************************************************

PI  TGTTAGTTTCCCGTTTCTGCCTGCTTCCATAATTCCCGGCCGGCGAAGTACTAGATCAACCTCCACGGTTTC
LB  TGTTAGTTTCCCGTTTCTGCCTGCTTCCATAATTCCCGGCCGGCGAAGTACTAGATCAACCTCCACGGTTTC
    ************************************************************************

PI  AAAAAGTAGGAAATATCATACCATCGGAATGACCGCTGCTTAGTAAATATCCATTGTTGTTTGTAATCTTGC
LB  AAAAAGTAGGAAATATCATACCATCGGAATGACCGCTGCTTAGTAAATATCCATTGTTGTTTGTAATCTTGC
    ************************************************************************

PI  TGAGAAAGCAACGTTACCATTTGCCTCATGGCAAAGACCTGTATGTTGAGGTGCTAAATCTTTTCTAGTTTT
LB  TGAGAAAGCAACGTTACCATTTGCCTCATGGCAAAGACCTGTATGTTGAGGTGCTAAATCTTTTCTAGTTTT
    ************************************************************************

PI  GTACCACTGAGGGTATGAGTGGCGCTAACGGAAAAGGGTAAGCAAGTTTGATTGGCTTACCTTCAGCCTCCT
LB  GTACCACTGAGGGTATGAGTGGCGCTAACGGAAAAGGGTAAGCAAGTTTGATTGGCTTACCTTCAGCCTCCT
    ************************************************************************

PI  TGGTTGTTTGAAGCATAGGTGCTTGCATGCATGTATCAAGCTGGTCACGTGATGAAAACGCGTAAGAATCAA
LB  TGGTTGTTTGAAGCATAGGTGCTTGCATGCATGTATCAAGCTGGTCACGTGATGAAAACGCGTAAGAATCAA
    ************************************************************************

PI  AGTCAGTTAAATTAAGATATAAACAGATGCAGTCATATTTTAAGCTAGTGCTGCACTGTGAACTTCAGTATC
LB  AGTCAGTTAAATTAAGATATAAACAGATGCAGTCATATTTTAAGCTAGTGCTGCACTGTGAACTTCAGTATC
    ************************************************************************

PI  TCAGATCAAAGAATTGAATAATGCTACCCCCTGTTTCTGCGCTGTTCATTTGGAAAAGACTGTCATGAACAT
LB  TCAGATCAAAGAATTGAATAATGCTACCCCCTGTTTCTGCGCTGTTCATTTGGAAAAGACTGTCATGAACAT
    ************************************************************************

PI  CCTAATTGGTAGCCATGCATTTATCAGCTTGCCGGCTTTATTTCTTTGCTCTCATTCCTTTTCATTTG
LB  CCTAATTGGTAGCCATGCATTTATCAGCTTGCCGGCTTTATTTCTTTGCTCTCATTCCTTTTCATTTG
    ********************************************************************
```

Figure S2 Sequence comparison of the 1149-bp fragment amplified by the primer pair Intr1/B/F with Intr1/B/R4 indicates no sequence variation within the first intron of the *VRN-B1* gene in Lebsock (LB) and PI 94749 (PI).
