# Supplementary material for: A Novel Retrotransposon Inserted in the Dominant Vrn-B1 Allele Confers Spring Growth Habit in Tetraploid Wheat (Triticum turgidum L.)
Source: G3 (Bethesda). 2011 Dec 1;1(7):637–45. doi: 10.1534/g3.111.001131 (PMC3276170; doi:10.1534/g3.111.001131)
Supplement: Supporting Information [file supp_1.7.637_FigureS3.pdf]

```
AY   CCCCTGCTACCAGTGCCTACTACTAGGACGGGCGAGTATCTTCATTCATTCCCGAAATACGCGGGTCGGCCA
LB   CCCCTGCTACCAGTGCCTACTACTAGGACGGGCGAGTATCTTCATTCATTCCCGAAATACGCGGGTCGGCCA
     ************************************************************************

AY   AAAGTAGAAAAATGCACTGCGCCCACCCAACCCACGCAGCGCACTGCACAGTAACGCTTCCTGTCAAAAGTC
LB   AAAGTAGAAAAATGCACTGCGCCCACCCAACCCACGCAGCGCACTGCACAGTAACGCTTCCTGTCAAAAGTC
     ************************************************************************

AY   CAGCTCAATCATGCACGCACACACGGTAGACGCGGTGCGAACGACCCGTCGTGGCACGAGCACGCGGTGTCT
LB   CAGCTCAATCATGCACGCACACACGGTAGACGCGGTGCGAACGACCCGTCGTGGCACGAGCACGCGGTGTCT
     ************************************************************************

AY   GCCCGCCCGTCCGCCCCGCAGCCGCCCTCCCAAACGGGACAAGCTAGACGGCCCAAAACAAGAAAGGAAAGC
LB   GCCCGCCCGTCCGCCCCGCAGCCGCCCTCCCAAACGGGACAAGCTAGACGGCCCAAAACAAGAAAGGAAAGC
     ************************************************************************

AY   AGCCTCCTACTGTGGCAGCCCGCCCCCACGACCGTCATCTCGCCTTCCATGCCATTTTCCCTGGACGGACAG
LB   AGCCTCCTACTGTGGCAGCCCGCCCCCACGACCGTCATCTCGCCTTCCATGCCATTTTCCCTGGACGGACAG
     ************************************************************************

AY   ACCCGTCCGAGCCGCCCTGACCTAGCCAGCCAGCCAGCCAGCATTTCCTGTTTCGTCCCGCGCCGCCGTGAC
LB   ACCCGTCCGAGCCGCCCTGACCTAGCCAGCCAGCCAGCCAGCATTTCCTGTTTCGTCCCGCGCCGCCGTGAC
     ************************************************************************

AY   CAAAAAAGCAAAAAATTAAAAAGGAAAATGCTAAAGGAAAAACTCTGCTCTTTCCCTTCTACTAGGCCTAGG
LB   CAAAAAAGCAAAAAATTAAAAAGGAAAATGCTAAAGGAAAAACTCTGCTCTTTCCCTTCTACTAGGCCTAGG
     ************************************************************************

AY   GTACAGTAGAATAGTAGTATAAAAAGGACAATTGTGCTCTTTTTTTTTGCTCTGTGGTGTGTGTTTGTGGCG
LB   GTACAGTAGAATAGTAGTATAAAAAGGACAATTGTGCTCTTTTTTTTTGCTCTGTGGTGTGTGTTTGTGGCG
     ************************************************************************

AY   AGAGAAAATGATTTGGGGAAAGCAATATCGGGAGATTCGCACGTAAGATCGTTCGACACGTCGACACCGGGC
LB   AGAGAAAATGATTTGGGGAAAGCAATATCGGGAGATTCGCACGTAAGATCGTTCGACACGTCGACACCGGGC
     ************************************************************************

AY   GGGCCCGTGGTGGGGCATCGTGTGGCTGCAGTACCGCGGGGCCCCGCGGGTCGGGCTGGGCCAATGGTTGCT
LB   GGGCCCGTGGTGGGGCATCGTGTGGCTGCAGTACCGCGGGGCCCCGCGGGTCGGGCTGGGCCAATGGTTGCT
     ************************************************************************

AY   CGACAGCGGCTATGCTGCAGACCAGCCCGGTATTGCATACCGCGCTCGGGGCCAGATCCCTTTAAAAACCCC
LB   CGACAGCGGCTATGCTGCAGACCAGCCCGGTATTGCATACCGCGCTCGGGGCCAGATCCCTTTAAAAACCCC
     ************************************************************************

AY   TCCCCCCACTTGCCGGAAACCTCGTTTTGGCCTGGCCATCCTCCCTCTCCTCCCTCTCTTCCGCCTCACCCA
LB   TCCCCCCACTTGCCGGAAACCTCGTTTTGGCCTGGCCATCCTCCCTCTCCTCCCTCTCTTCCGCCTCACCCA
     ************************************************************************

AY   ACCACCTGACAGCCATGGCTCCGCCCCCCCGCCCCCGCCTGCGCCTGTCGGAGTAGCCGTCGCGGTCTGCCG
LB   ACCACCTGACAGCCATGGCTCCGCCCCCCCGCCCCCGCCTGCGCCTGTCGGAGTAGCCGTCGCGGTCTGCCG
     ************************************************************************

AY   GTGTTGGAGGCTTGGGGTGTAGGGTTGGCCCCGTTCTCCAGCGGAGATGGGGC
LB   GTGTTGGAGGCTTGGGGTGTAGGGTTGGCCCCGTTCTCCAGCGGAGATGGGGC
     *****************************************************
```

Figure S3 DNA sequence comparison indicates that the 989-bp fragment produced by the primer pair VRNBPF1/VRNBPR1 in Lebsock is the same as the reported *VRN-B1* gene in durum wheat 'Langdon' (BAC clone 1225D16, GeneBank accession number AY616453) (Yan et al. 2004a). LB and AY represents Lebsock and AY616453, respectively.
