# Supplementary material for: A Novel Retrotransposon Inserted in the Dominant Vrn-B1 Allele Confers Spring Growth Habit in Tetraploid Wheat (Triticum turgidum L.)
Source: G3 (Bethesda). 2011 Dec 1;1(7):637–45. doi: 10.1534/g3.111.001131 (PMC3276170; doi:10.1534/g3.111.001131)
Supplement: Supporting Information [file supp_1.7.637_FigureS4.pdf]

```
PI   CCCCTGCTACCAGTGCCTACTACTAGGACGGGCGAGTATCTTCATTCATTCCCGAAATACGCGGGTCGGCC
LB   CCCCTGCTACCAGTGCCTACTACTAGGACGGGCGAGTATCTTCATTCATTCCCGAAATACGCGGGTCGGCC
     ***********************************************************************

PI   AAAAGTAGAAAAATGCACTGCGCCCACCCAACCCACGCAGCGCACTGCACAGTAACGCTTCCTGTCAAAAG
LB   AAAAGTAGAAAAATGCACTGCGCCCACCCAACCCACGCAGCGCACTGCACAGTAACGCTTCCTGTCAAAAG
     ***********************************************************************

PI   TCCAGCTCAATCATGCACGCACACACGGTAGACGCGGTGCGAACGACCCGTCGTGGCACGAGCACGCGGTG
LB   TCCAGCTCAATCATGCACGCACACACGGTAGACGCGGTGCGAACGACCCGTCGTGGCACGAGCACGCGGTG
     ***********************************************************************

PI   TCTGCCCGCCCGTCCGCCCCGCAGCCGCCCTCCCAAACGGGACAAGCTAGACGGCCCAAAACAAGAAAGGA
LB   TCTGCCCGCCCGTCCGCCCCGCAGCCGCCCTCCCAAACGGGACAAGCTAGACGGCCCAAAACAAGAAAGGA
     ***********************************************************************

PI   AAGCAGCCTCCTACTGTGGCAGCCCGCCCCCACGACCGTCATCTCGCCTTCCATGCCATTTTCCCTGGACG
LB   AAGCAGCCTCCTACTGTGGCAGCCCGCCCCCACGACCGTCATCTCGCCTTCCATGCCATTTTCCCTGGACG
     ***********************************************************************

PI   GACAGACCCGTCCGAGCCGCCCTGACCTAGCCAGCCAGCCAGCCAGCATTTCCTGTTTCGTCCCGCGCCGC
LB   GACAGACCCGTCCGAGCCGCCCTGACCTAGCCAGCCAGCCAGCCAGCATTTCCTGTTTCGTCCCGCGCCGC
     ***********************************************************************

PI   CGTGACCAAAAAAGCAAAAAATTAAAAAGGAAAATGCTAAAGGAAAAACTCTGCTCTTTCCCTTCTACTAG
LB   CGTGACCAAAAAAGCAAAAAATTAAAAAGGAAAATGCTAAAGGAAAAACTCTGCTCTTTCCCTTCTACTAG
     ***********************************************************************

PI   GCCTAGGGTACAGTAGAATAGTAGTATAAAAAGGACAATTGTGCTCTTTTTTTTTGCTCTGTGGTGTGTGT
LB   GCCTAGGGTACAGTAGAATAGTAGTATAAAAAGGACAATTGTGCTCTTTTTTTTTGCTCTGTGGTGTGTGT
     ***********************************************************************

PI   TTGTGGCGAGAGAAAATGATTTGGGGAAAGCAATATCGGGAGATTCGCACGTAAGATCGTTCGACACGTCG
LB   TTGTGGCGAGAGAAAATGATTTGGGGAAAGCAATATCGGGAGATTCGCACGTAAGATCGTTCGACACGTCG
     ***********************************************************************

PI   ACACCGGGCGGGCCCGTGGTGGGGCATCGTGTGGCTGCAGTACCGCGGGGCCCCGCGGGTCGGGCTGGGCC
LB   ACACCGGGCGGGCCCGTGGTGGGGCATCGTGTGGCTGCAGTACCGCGGGGCCCCGCGGGTCGGGCTGGGCC
     ***********************************************************************

PI   AATGGTTGCTCGACAGCGGCTATGCTGCAGACCAGCCCGGTATTGCATACCGCGCTCGGGGCCAGATCCCT
LB   AATGGTTGCTCGACAGCGGCTATGCTGCAGACCAGCCCGGTATTGCATACCGCGCTCGGGGCCAGATCCCT
     ***********************************************************************

PI   TTAAAAACCCCTCCCCCCACTTGCCGGAAACCTCGTTTTGGCCTGGCCATCCTCCCTCTCCTCCCTCTCTT
LB   TTAAAAACCCCTCCCCCCACTTGCCGGAAACCTCGTTTTGGCCTGGCCATCCTCCCTCTCCTCCCTCTCTT
     ***********************************************************************

PI   CCGCCTCACCCAACCACCTG
LB   CCGCCTCACCCAACCACCTG
     ********************
```

Figure S4 Comparison of DNA sequence in the 872-bp fragment produced by primer pair VRNBPF1/VRNBP R2 in Lebsock (LB) and PI 94749 (PI) suggests no allelic variation in the corresponding region close to *VRN-B1* promoter.
