# Supplementary material for: A Novel Retrotransposon Inserted in the Dominant Vrn-B1 Allele Confers Spring Growth Habit in Tetraploid Wheat (Triticum turgidum L.)
Source: G3 (Bethesda). 2011 Dec 1;1(7):637–45. doi: 10.1534/g3.111.001131 (PMC3276170; doi:10.1534/g3.111.001131)
Supplement: Supporting Information [file supp_1.7.637_FigureS5.pdf]

CTCCGTGTCACGACTGTCAAGGTGACCTCACCAGCCAACAAGCGGGAAGAGGAACTGGTCAAGGCGCTTGCTGATTCGACAGAAGGGCCGAC
CCGTTAGCAGGCCGCGGTGTCTTGGGTGCGTGGCCTTGTGGGCCGTATCGAGCCTGCGAGCTCAGGGTTAAGTAAGTGGCTTGGCACCGTCA
AGGGCATCAAATTGTATCTCACTGAATAGACAGAGAGAAGCTGCTCTTCTCCTGGTTATCCCCTTCCTTCTCCACCTACCTCAATCTCACCT
GCCCCCTTCCTCCTCCTCTCAGATCTGATCGATTAGGTAGATCGGGCCGTTACAAGTGGTATCAGAGGTCGGGCGATCTGGATAACCCGTAC
PBS
CGCAAGCCGCGCTACGCCACCAAGCAAGTTACGCAAGCTGCAGCGATGGAGGAGCAATTGGCGGCGCTGGCCAAGGCGGTCAACGACGGCCG
CACCGCCGACGAGGCCCGGCTCGAGGCCATCCAGACCTCGCTCGAGTTGTGGCGTCCAGCGGTCACCAACCTGCAACAACAACTCAACGAGC
TCCAATCCCAAGTGGGGCGGATCGCACTCCACCCCGCGCTGGCAGATCCGCAACAGCCACCGGTGGAACAAGTGGTGCACGGCGCGCCGACC
GAGTCGGCGGGCGACTTCGAGCACCACGGGCCATCTGGCCACGGCGAGATCGACAAAACCGGGGGTCGGGCACACGGGGTAGTCACTACCCT
CGCGCCACCTCCGGTCAAGGGTGCGTACTCATCCCAATCTATCATACCTGCTTCTCCTCGCGGTGATAGTGGTCCAGAGGCGGAAAGGAGGG
GCACTCAGGACAACCCGTTCGTTCCATTCGCGCATCACGCTCACTGGGCTCTGCCCAAGATGGATTTTCCATCGTTTGATGGAGAAAATCCG
CAGTTCTGGAAGACCAAATGTGAAAAGTATTTTGATGTGTATGGAGTGGCTCCGGATCTCTGGGTTCGATTAGCCACGCTGAATTTCACAGG
CACAGCGGCCAGATGGCTGCAACTTCATGAGACACAGAGCACTTCATTCACTTGGGCGTCACTCTGTGAGGCCTTGTGCCATAAATTTGGTA
GAGAACAGTACCAGTCCCATCTCCGTCAGTTTAACACCCTTCGTCAGTCAGGGACTGTAGCGGATTACATGACACGATTTGAAGAATTGATG
CATCACATATTAGCTCACAACCCAGCGTTCGACTCCGTGTATTTTACTACCCAGTTTCTAGATGGTCTTAAGGGAGAGATTCGTGCTGTTGT
TATGCTTCATCAACCAAAAGACTTGGACTCCGCCTTTTCCTTGGCTACATTGCAGGAGGAGCTGATGGAGGCGTTACCGCGAAGGGAGTACA
AGCGACAGGATGCAGCTAACCAGCGGTCACCTGCACAACGGCCTTTGCTGGCGATTGGTGCTCCGCCCGTCCGGCAGGTGTTGCCAGGACCG
CCACCTGCAGCTGAAGATCGACGGGCGATCGATGCGGCAAACCCTCCGGATCGCCGCGATCAAGGCAGAGGTGATGATCGGGTAGCAGCCCT
CCGTAACTACAGGCGTGCTCGGGGGCTCTGTTTTAAATGTGGTGAGCGATGGGGCCAAGGGCATCAATGCGGGCCTACAGTTCAATTACATG
TGGTCGAAGAACTGCTAGAACTGTTGCAAGCTGATCAGGGCGTTCCAGTAGTGCCTGACCCTGATTCTGATGAAGATGTTCTGATGTGCATC
TCCAAGGGAGCCACCACGGGGCAAACTACTCCCCGTACAGTCAGGTTGCTGGGGCAGATAGGTGGACAAGAGATGTTGATCCTCGTGGATTC
GGGCAGCTCTCATAGCTTTCTCAGTGATACAGTGGTGGCGCGACTTCAACTACCAATTCAAGCCATGTCCACGGTTGCAGTCAAAATAGCAG
ACGGGGGAACTCTATCATGTTCAGGTGTGGTGCCAGAGTGCAGATGGAAGACACAAGGACATGAATTTGTCACTGACCTCAGAGTTTTAGCG
CTTGGGTGCTATGATATGATAGTTGGCATGGACTGGCTGGAGTCATGTGGGCCTATGTGGATCGATTGGTCCGCAAAGCAACTGATATTCAA
CCACGGCGGGCAGCAAATTCAGTTGGCAGGGGTGCAAACGCAATTGCGACAAGTCCAACCAATCTCCTCGGCTCAGTTGTGTGCACTGGAAG
AGGCTAATGCGGTGGCTCACATCATCTGTTTGCATGCGGTGGGGGATGATGTAGTGGTGGAACACATTCCAGTGGAAGTACAAGCTGTGCTG
CAAGAATACAGTGTGGTGTTTGAAAAGCCTACTGATCTACCTCCACACCGCGCTTGGGACCATGCAATTCCAATCATCCCTGGAGCCAAGCC
GGTCAACATTCGACCGTATCGGTATACCCCGGAACAAAAGACAGAGATTGAGCTTCAGGTGAAGGAAATGCTTAAAGCTGGACTAATCGTGC
CTAGTACCAGTCCGTTCTCCTCGCCTGTTCTGTTAGTGAAAAAGAAAGATATGACATGGCGCCTCTGCGTGGACTATCGTCACCTGAATGCA
ATTACACTAAAAAGCACCTATCCATTGCCAGTCATTGACGAGCTGCTCGATGAGCTTGCAGGATCGTGTTGGTTCTCAAAGATGGATCTACG
AGCGGGCTACCACCAAATCAGGTTGAGAGAAGAAGATGAACCAAAAACAGCCTTTACAACCCATCAAGGGCACTTTCAGTTTCGGGTGTTGC
CGTATGGTGTGACCGGAGGGCCAGCAACATTCCAAGGAGGGATGAACACAGTGCTGGGTCCTTTGCTCAGGCACGGTGTGTGTGTTTTCATG
GACGACATCCTCACCCACTCAGCAACCTTAGAGGGACACGTGGAACTGCTGCGGCAAGTGTTGAGTATTTTGGCACAACATGGTTTGAAGGT
CAAAATGTCCAAATGCTCCTTTGCACAACGCAAGATCGATTTCTTGGGGCACACCATAAGCAAGGAAGGTGTAACCACTGATGAGAGCAAGA
TTGCAACAGTCCGGGACTGGCCGCGCCCAGGGTCAGTTCGCGAGCTACGAGGCTTCTTGGGGCTTGCGGGATACTACCGTAAGTTCGTCAGA
AACTTCGGCGTCATATCCCGTCCTCTGACAGACATGTTGAAGAAGGGCACCCCTCTTTATTTGGACACCGCTAGCAGAAACAGCATTTGCAGA
GTTGAAGCAGGCACTCATCCAGGCCCCCGTGCTCGCATTACCTGATTTCAACAAGAAGTTCGTCGTAGAGACTGACGCAAGTGCCAAGGGCG
TCGGTGCAGTCCTGATGCAGGACTTCCACCCGTTAGCTTACTTGAGCAAGGCGTTAGCGCCACGCAACCTTGGTCTATCAGCATATGAAAAA
GAGTGTCTTGCCTTGATTTTAGCAGTTGATCACTGGAGGCCGTATTTACAGCATGCCGAGTTTCTGGTGCGCACCGATCAAAAGAGCTTGCT
GAATCTGACCGACCAGAGGCTCAACACACCAATCCAGCAGCGGGCTTTCACCAAGTTAGTGGGGCTGCAGTTCCAGATCCAGTACAAGGCAG
GCATCACTAACAAAGCAGCAGATGCGCTCTCACGGCGCGAACATGACACTGAAGCAGCAGTGGCAGCGATTTCAATTTGCAAGCCCGCCTGG
TTGGAAGCAGTGGCCGTCAGTTATCGCGAAGACAAAGAAATTCAAGACAAGATGGCGCAAATCGCACTAGATCCTGGAAGTGACTCGGATTA
CTCTCTGAAGGATGGAGTCATGCGTTACAAGGGGCGTATCTGGATTGGCTCGGATAGTATGATACAACAATCGCTGGTCAAGGCGCTCCACG
ACAGTGCAGTGGGTGGACATTCAGGGTTCTACGCCACGTATCACAGAATCAAAAACCTCTTCTTCTGGAAAGGCATGAAGGCTCAGATTAAG
CAATATGTCAAGGAGTGTGTCACTTGTCAGCGTGCGAAAACAGAACGAATCGCTCCAGCGGGGCTGTTACAACCCCTCCCCATTCCAAAGCG
GCCATGGGCAGTGATCTCCCTGGATTTTATTGAAGGCCTTCCCAAAATCCGGAGGCCATGATGTGATTTTGGTCGTGGTGGATAAGTTCTCC
AAATATGCTCACTTCGTTCCACTGACTCATCCATTCACGGCTCTGACGGTGGCTACGGCCTTCATGAAAAACATCTTTCGGCTTCACGGGCT
GCCTCTAGCTATTATATCTGACCGAGACCGGATCTTCACAAGCACAATTTGGCAAGAGTTGTTCAAACTGTCTCAGACCCAGCTGCGTTTGA
GTTCGTCATACCATCCCCAAACTGATGGGCAGACAGAGCGCGTAAACCAGTGCTTAGAAGGATACCTGCGCTGTGCAGTGCACTCGTGTCCT
GGTAATTGGATCAAGTGGCTATTCTTAGCCGAGTATTGGTATAACACGACATTTCACTCCTCTCTGGGGCGCACTCCATTTGAAGTAATCTA
TGGCCATCTGCCAAGGGAGTTCGCTGTTACTCAAGTGGAAGAAAGTTCAGTGCCGGACCTGGCAGCTTGGCTACAGGAACGAGAAGTGATGG
CTCAGCACTTACAGCAACAACTGAAACATGCTCAGGATCGCATGAAAGCTCAGGCCGACAAACACCGCACTGACCGTTCATTCGAAGTGGGT
GACATGGTTTTCCTCAAGCTCCAGCCACCATATACAAACATCCGTAGCCCAACGCCCTTACCAGAAAGCTGGCATTCCGCTACTACGGGCCC
ATACCCAAGTGCTCGCTCGGATCGGAAAAAGTGGCATACAAACTTCAGTTACCAGCTGACAGTAAGATACATTCCGTGGTGCACGTCTCACA
ACTAAAAAAGGCTGTTGGTCCTAGCACCCCAGGTGAGCTGCGATCTTCCTCCTGTTAACTCTATTCTGCAGGCTGAACATCAACCAGAGGCA
ATTCTGGACACCAAGTTCATCCGCGTCCGGGGAGAGATGCAACCTCGTCTTCTGGTACAATGGGGTGCGCTGCCGGCGTCGCTCGCCACCTG
GGAGGAGCCCGCTGAACTGCGTCGTCGCTTTCCAGCAGCATCGGCTTGGGGACAAGCCTCACCTCAAGGGGGGGAGGATGTCACGACTGTCA
PPT
AGGTGACCTCACCAGCCAACAAGCGGGAAGAGGAACTGGTCAAGGCGCTTGCTGATTCGACAGAAGGGCCGACCCGTTAGCAGGCCGCGGTG
TCTTGGGTGCGTGGCCTTGTGGGCCGTATCGAGCCTGCGAGCTCAGGGTTAAGTAAGTGGCTTGGCACCGTCAAGGGCATCAAATTGTATCT
CACTGAATAGACAGAGAGAAGCTGCTCTTCTCCTGGTTATCCCCTTCCTTCTCCACCTACCTCAATCTCACCTGCCCCCTTCCTCCTCCTCT
CAGATCTGATCGATTAGGTAGATCGGGCCGTTACA**CTCCG**

Figure S5 DNA sequence of the 5,463-bp insertion in the dominant *Vrn-B1* allele carried by PI 94749. The two CTCCG motifs on both ends of the insertion are shown in bold and highlighted in gray. The short inverted repeat 5′-TG...CA-3′ on the ends of the long terminal repeat (LTR) is highlighted in green, and LTR is shown in red. The motifs primer binding site (PBS) and polypurine tract (PPT) are underlined but highlighted in light blue and purple, respectively. For sequence annotation of the insertion that predicted by computer program GenScan at GeniusNet, the start codon (ATG) and stop codon (TGA) are shown in red with yellow highlight, and an open reading frame is highlighted in gray. The 1,231 amino acids encoded by the deduced coding

region are shown in Figure S6. The sequence data of the insertion has been deposited with the GenBank Data Libraries under accession number HQ186251.
