# Supplementary material for: A Novel Retrotransposon Inserted in the Dominant Vrn-B1 Allele Confers Spring Growth Habit in Tetraploid Wheat (Triticum turgidum L.)
Source: G3 (Bethesda). 2011 Dec 1;1(7):637–45. doi: 10.1534/g3.111.001131 (PMC3276170; doi:10.1534/g3.111.001131)
Supplement: Supporting Information [file supp_1.7.637_FigureS6.pdf]

```
MEEQLAALAKAVNDGRTADEARLEAIQTSLELWRPAVTNLQQQLNELQSQVGRIALHPALADPQQPPVEQV
VHGAPTESAGDFEHHGPSGHGEIDKTGGRAHGVVTTLAPPPVKGAYSSQSIIPASPRGDSGPEAERRGTQD
NPFVPFAHHAHWALPKMDFPSFDGENPQFWKTKCEKYFDVYGVAPDLWVRLATLNFTGTAARWLQLHETQS
TSFTWASLCEALCHKFGREQYQSHLRQFNTLRQSGTVADYMTRFEELMHHILAHNPAFDSVYFTTQFLDGL
KGEIRAVVMLHQPKDLDSAFSLATLQEELMEALPRREYKRQDAANQRSPAQRPLLAIGAPPVRQVLPGPPP
AAEDRRAIDAANPPDRRDQGRGDDRVAALRNYRRARGLCFKCGERWGQGHQCGPTVQLHVVEELLELLQAD
QGVPVVPDPDSDEDVLMCISKGATTGQTTPRTVRLLGQIGGQEMLILVDSGSSHSFLSDTVVARLQLPIQA
MSTVAVKIADGGTLSCSGVVPECRWKTQGHEFVTDLRVLALGCYDMIVGMDWLESCGPMWIDWSAKQLIFN
HGGQQIQLAGVQTQLRQVQPISSAQLCALEEANAVAHIICLHAVGDDVVVEHIPVEVQAVLQEYSVVFEKP
TDLPPHRAWDHAIPIIPGAKPVNIRPYRYTPEQKTEIELQVKEMLKAGLIVPSTSPFSSPVLLVKKKDMTW
RLCVDYRHLNAITLKSTYPLPVIDELLDELAGSCWFSKMDLRAGYHQIRLREEDEPKTAFTTHQGHFQFRV
LPYGVTGGPATFQGGMNTVLGPLLRHGVCVFMDDILTHSATLEGHVELLRQVLSILAQHGLKVKMSKCSFA
QRKIDFLGHTISKEGVTTDESKIATVRDWPRPGSVRELRGFLGLAGYYRKFVRNFGVISRPLTDMLKKGTL
FIWTPLAETAFAELKQALIQAPVLALPDFNKKFVVETDASAKGVGAVLMQDFHPLAYLSKALAPRNLGLSA
YEKECLALILAVDHWRPYLQHAEFLVRTDQKSLLNLTDQRLNTPIQQRAFTKLVGLQFQIQYKAGITNKAA
DALSRREHDTEAAVAAISICKPAWLEAVAVSYREDKEIQDKMAQIALDPGSDSDYSLKDGVMRYKGRIWIG
SDSMIQQSLVKALHDSAVGGHSGFYATYHRIKNLFFWKGMKAQIKQYVKECVTCQRAKTERIAPAGLLQPL
PIPKRPWAVISLDFIEGLPKIRRP*
```

Figure S6 The 1,231 amino acids in the deduced protein predicted by the web-based computer program GenScan at GeniusNet (http://genome.dkfz-heidelberg.de/cgi-bin/GENSCAN/genscan.cgi).
