# Supplementary material for: A Novel Retrotransposon Inserted in the Dominant Vrn-B1 Allele Confers Spring Growth Habit in Tetraploid Wheat (Triticum turgidum L.)
Source: G3 (Bethesda). 2011 Dec 1;1(7):637–45. doi: 10.1534/g3.111.001131 (PMC3276170; doi:10.1534/g3.111.001131)
Supplement: Supporting Information [file supp_1.7.637_FigureS7.pdf]

Comparison of 600-bp sequence amplified by primers VBINS2F (5’-CTCACCCAACCACCTGACAGC-3’) and VBINS2R (5’-ATCCGCCCCACTTGGGATT-3’):

```
PI 499972       CTCACCCAACCACCTGACAGCCATGGCTCCGTGTCACGACTGTCAAGGTGACCTCACCAG
PI 532501       CTCACCCAACCACCTGACAGCCATGGCTCCGTGTCACGACTGTCAAGGTGACCTCACCAG
PI 352278       CTCACCCAACCACCTGACAGCCATGGCTCCGTGTCACGACTGTCAAGGTGACCTCACCAG
PI 115816       CTCACCCAACCACCTGACAGCCATGGCTCCGTGTCACGACTGTCAAGGTGACCTCACCAG
PI 283887       CTCACCCAACCACCTGACAGCCATGGCTCCGTGTCACGACTGTCAAGGTGACCTCACCAG
PS5             CTCACCCAACCACCTGACAGCCATGGCTCCGTGTCACGACTGTCAAGGTGACCTCACCAG
CItr 7779       CTCACCCAACCACCTGACAGCCATGGCTCCGTGTCACGACTGTCAAGGTGACCTCACCAG
CItr 3686       CTCACCCAACCACCTGACAGCCATGGCTCCGTGTCACGACTGTCAAGGTGACCTCACCAG
PI 113961       CTCACCCAACCACCTGACAGCCATGGCTCCGTGTCACGACTGTCAAGGTGACCTCACCAG
PI 585017       CTCACCCAACCACCTGACAGCCATGGCTCCGTGTCACGACTGTCAAGGTGACCTCACCAG
PI 532486       CTCACCCAACCACCTGACAGCCATGGCTCCGTGTCACGACTGTCAAGGTGACCTCACCAG
PI 532475       CTCACCCAACCACCTGACAGCCATGGCTCCGTGTCACGACTGTCAAGGTGACCTCACCAG
PI 470732       CTCACCCAACCACCTGACAGCCATGGCTCCGTGTCACGACTGTCAAGGTGACCTCACCAG
PI 352281       CTCACCCAACCACCTGACAGCCATGGCTCCGTGTCACGACTGTCAAGGTGACCTCACCAG
PI 349040       CTCACCCAACCACCTGACAGCCATGGCTCCGTGTCACGACTGTCAAGGTGACCTCACCAG
PI 283889       CTCACCCAACCACCTGACAGCCATGGCTCCGTGTCACGACTGTCAAGGTGACCTCACCAG
PI 251914       CTCACCCAACCACCTGACAGCCATGGCTCCGTGTCACGACTGTCAAGGTGACCTCACCAG
PI 61102        CTCACCCAACCACCTGACAGCCATGGCTCCGTGTCACGACTGTCAAGGTGACCTCACCAG
PI 352282       CTCACCCAACCACCTGACAGCCATGGCTCCGTGTCACGACTGTCAAGGTGACCTCACCAG
PI 286070       CTCACCCAACCACCTGACAGCCATGGCTCCGTGTCACGACTGTCAAGGTGACCTCACCAG
PI 115817       CTCACCCAACCACCTGACAGCCATGGCTCCGTGTCACGACTGTCAAGGTGACCTCACCAG
PI 94749        CTCACCCAACCACCTGACAGCCATGGCTCCGTGTCACGACTGTCAAGGTGACCTCACCAG
PI 94748        CTCACCCAACCACCTGACAGCCATGGCTCCGTGTCACGACTGTCAAGGTGACCTCACCAG
                ************************************************************

PI 499972       CCAACAAGCGGGAAGAGGAACTGGTCAAGGCGCTTGCTGATTCGACAGAAGGGCCGACCC
PI 532501       CCAACAAGCGGGAAGAGGAACTGGTCAAGGCGCTTGCTGATTCGACAGAAGGGCCGACCC
PI 352278       CCAACAAGCGGGAAGAGGAACTGGTCAAGGCGCTTGCTGATTCGACAGAAGGGCCGACCC
PI 115816       CCAACAAGCGGGAAGAGGAACTGGTCAAGGCGCTTGCTGATTCGACAGAAGGGCCGACCC
PI 283887       CCAACAAGCGGGAAGAGGAACTGGTCAAGGCGCTTGCTGATTCGACAGAAGGGCCGACCC
PS5             CCAACAAGCGGGAAGAGGAACTGGTCAAGGCGCTTGCTGATTCGACAGAAGGGCCGACCC
CItr 7779       CCAACAAGCGGGAAGAGGAACTGGTCAAGGCGCTTGCTGATTCGACAGAAGGGCCGACCC
CItr 3686       CCAACAAGCGGGAAGAGGAACTGGTCAAGGCGCTTGCTGATTCGACAGAAGGGCCGACCC
PI 113961       CCAACAAGCGGGAAGAGGAACTGGTCAAGGCGCTTGCTGATTCGACAGAAGGGCCGACCC
PI 585017       CCAACAAGCGGGAAGAGGAACTGGTCAAGGCGCTTGCTGATTCGACAGAAGGGCCGACCC
PI 532486       CCAACAAGCGGGAAGAGGAACTGGTCAAGGCGCTTGCTGATTCGACAGAAGGGCCGACCC
PI 532475       CCAACAAGCGGGAAGAGGAACTGGTCAAGGCGCTTGCTGATTCGACAGAAGGGCCGACCC
PI 470732       CCAACAAGCGGGAAGAGGAACTGGTCAAGGCGCTTGCTGATTCGACAGAAGGGCCGACCC
PI 352281       CCAACAAGCGGGAAGAGGAACTGGTCAAGGCGCTTGCTGATTCGACAGAAGGGCCGACCC
PI 349040       CCAACAAGCGGGAAGAGGAACTGGTCAAGGCGCTTGCTGATTCGACAGAAGGGCCGACCC
PI 283889       CCAACAAGCGGGAAGAGGAACTGGTCAAGGCGCTTGCTGATTCGACAGAAGGGCCGACCC
PI 251914       CCAACAAGCGGGAAGAGGAACTGGTCAAGGCGCTTGCTGATTCGACAGAAGGGCCGACCC
PI 61102        CCAACAAGCGGGAAGAGGAACTGGTCAAGGCGCTTGCTGATTCGACAGAAGGGCCGACCC
PI 352282       CCAACAAGCGGGAAGAGGAACTGGTCAAGGCGCTTGCTGATTCGACAGAAGGGCCGACCC
PI 286070       CCAACAAGCGGGAAGAGGAACTGGTCAAGGCGCTTGCTGATTCGACAGAAGGGCCGACCC
PI 115817       CCAACAAGCGGGAAGAGGAACTGGTCAAGGCGCTTGCTGATTCGACAGAAGGGCCGACCC
PI 94749        CCAACAAGCGGGAAGAGGAACTGGTCAAGGCGCTTGCTGATTCGACAGAAGGGCCGACCC
PI 94748        CCAACAAGCGGGAAGAGGAACTGGTCAAGGCGCTTGCTGATTCGACAGAAGGGCCGACCC
                ************************************************************

PI 499972       GTTAGCAGGCCGCGGTGTCTTGGGTGCGTGGCCTTGTGGGCCGTATCGAGCCTGCGAGCT
PI 532501       GTTAGCAGGCCGCGGTGTCTTGGGTGCGTGGCCTTGTGGGCCGTATCGAGCCTGCGAGCT
PI 352278       GTTAGCAGGCCGCGGTGTCTTGGGTGCGTGGCCTTGTGGGCCGTATCGAGCCTGCGAGCT
PI 115816       GTTAGCAGGCCGCGGTGTCTTGGGTGCGTGGCCTTGTGGGCCGTATCGAGCCTGCGAGCT
```

```
PI 283887        GTTAGCAGGCCGCGGTGTCTTGGGTGCGTGGCCTTGTGGGCCGTATCGAGCCTGCGAGCT
PS5              GTTAGCAGGCCGCGGTGTCTTGGGTGCGTGGCCTTGTGGGCCGTATCGAGCCTGCGAGCT
CItr 7779        GTTAGCAGGCCGCGGTGTCTTGGGTGCGTGGCCTTGTGGGCCGTATCGAGCCTGCGAGCT
CItr 3686        GTTAGCAGGCCGCGGTGTCTTGGGTGCGTGGCCTTGTGGGCCGTATCGAGCCTGCGAGCT
PI 113961        GTTAGCAGGCCGCGGTGTCTTGGGTGCGTGGCCTTGTGGGCCGTATCGAGCCTGCGAGCT
PI 585017        GTTAGCAGGCCGCGGTGTCTTGGGTGCGTGGCCTTGTGGGCCGTATCGAGCCTGCGAGCT
PI 532486        GTTAGCAGGCCGCGGTGTCTTGGGTGCGTGGCCTTGTGGGCCGTATCGAGCCTGCGAGCT
PI 532475        GTTAGCAGGCCGCGGTGTCTTGGGTGCGTGGCCTTGTGGGCCGTATCGAGCCTGCGAGCT
PI 470732        GTTAGCAGGCCGCGGTGTCTTGGGTGCGTGGCCTTGTGGGCCGTATCGAGCCTGCGAGCT
PI 352281        GTTAGCAGGCCGCGGTGTCTTGGGTGCGTGGCCTTGTGGGCCGTATCGAGCCTGCGAGCT
PI 349040        GTTAGCAGGCCGCGGTGTCTTGGGTGCGTGGCCTTGTGGGCCGTATCGAGCCTGCGAGCT
PI 283889        GTTAGCAGGCCGCGGTGTCTTGGGTGCGTGGCCTTGTGGGCCGTATCGAGCCTGCGAGCT
PI 251914        GTTAGCAGGCCGCGGTGTCTTGGGTGCGTGGCCTTGTGGGCCGTATCGAGCCTGCGAGCT
PI 61102         GTTAGCAGGCCGCGGTGTCTTGGGTGCGTGGCCTTGTGGGCCGTATCGAGCCTGCGAGCT
PI 352282        GTTAGCAGGCCGCGGTGTCTTGGGTGCGTGGCCTTGTGGGCCGTATCGAGCCTGCGAGCT
PI 286070        GTTAGCAGGCCGCGGTGTCTTGGGTGCGTGGCCTTGTGGGCCGTATCGAGCCTGCGAGCT
PI 115817        GTTAGCAGGCCGCGGTGTCTTGGGTGCGTGGCCTTGTGGGCCGTATCGAGCCTGCGAGCT
PI 94749         GTTAGCAGGCCGCGGTGTCTTGGGTGCGTGGCCTTGTGGGCCGTATCGAGCCTGCGAGCT
PI 94748         GTTAGCAGGCCGCGGTGTCTTGGGTGCGTGGCCTTGTGGGCCGTATCGAGCCTGCGAGCT
                 ************************************************************

PI 499972        CAGGGTTAAGTAAGTGGCTTGGCACCGTCAAGGGCATCAAATTGTATCTCACTGAATAGA
PI 532501        CAGGGTTAAGTAAGTGGCTTGGCACCGTCAAGGGCATCAAATTGTATCTCACTGAATAGA
PI 352278        CAGGGTTAAGTAAGTGGCTTGGCACCGTCAAGGGCATCAAATTGTATCTCACTGAATAGA
PI 115816        CAGGGTTAAGTAAGTGGCTTGGCACCGTCAAGGGCATCAAATTGTATCTCACTGAATAGA
PI 283887        CAGGGTTAAGTAAGTGGCTTGGCACCGTCAAGGGCATCAAATTGTATCTCACTGAATAGA
PS5              CAGGGTTAAGTAAGTGGCTTGGCACCGTCAAGGGCATCAAATTGTATCTCACTGAATAGA
CItr 7779        CAGGGTTAAGTAAGTGGCTTGGCACCGTCAAGGGCATCAAATTGTATCTCACTGAATAGA
CItr 3686        CAGGGTTAAGTAAGTGGCTTGGCACCGTCAAGGGCATCAAATTGTATCTCACTGAATAGA
PI 113961        CAGGGTTAAGTAAGTGGCTTGGCACCGTCAAGGGCATCAAATTGTATCTCACTGAATAGA
PI 585017        CAGGGTTAAGTAAGTGGCTTGGCACCGTCAAGGGCATCAAATTGTATCTCACTGAATAGA
PI 532486        CAGGGTTAAGTAAGTGGCTTGGCACCGTCAAGGGCATCAAATTGTATCTCACTGAATAGA
PI 532475        CAGGGTTAAGTAAGTGGCTTGGCACCGTCAAGGGCATCAAATTGTATCTCACTGAATAGA
PI 470732        CAGGGTTAAGTAAGTGGCTTGGCACCGTCAAGGGCATCAAATTGTATCTCACTGAATAGA
PI 352281        CAGGGTTAAGTAAGTGGCTTGGCACCGTCAAGGGCATCAAATTGTATCTCACTGAATAGA
PI 349040        CAGGGTTAAGTAAGTGGCTTGGCACCGTCAAGGGCATCAAATTGTATCTCACTGAATAGA
PI 283889        CAGGGTTAAGTAAGTGGCTTGGCACCGTCAAGGGCATCAAATTGTATCTCACTGAATAGA
PI 251914        CAGGGTTAAGTAAGTGGCTTGGCACCGTCAAGGGCATCAAATTGTATCTCACTGAATAGA
PI 61102         CAGGGTTAAGTAAGTGGCTTGGCACCGTCAAGGGCATCAAATTGTATCTCACTGAATAGA
PI 352282        CAGGGTTAAGTAAGTGGCTTGGCACCGTCAAGGGCATCAAATTGTATCTCACTGAATAGA
PI 286070        CAGGGTTAAGTAAGTGGCTTGGCACCGTCAAGGGCATCAAATTGTATCTCACTGAATAGA
PI 115817        CAGGGTTAAGTAAGTGGCTTGGCACCGTCAAGGGCATCAAATTGTATCTCACTGAATAGA
PI 94749         CAGGGTTAAGTAAGTGGCTTGGCACCGTCAAGGGCATCAAATTGTATCTCACTGAATAGA
PI 94748         CAGGGTTAAGTAAGTGGCTTGGCACCGTCAAGGGCATCAAATTGTATCTCACTGAATAGA
                 ************************************************************


PI 499972        CAGAGAGAAGCTGCTCTTCTCCTGGTTATCCCCTTCCTTCTCCACCTACCTCAATCTCAC
PI 532501        CAGAGAGAAGCTGCTCTTCTCCTGGTTATCCCCTTCCTTCTCCACCTACCTCAATCTCAC
PI 352278        CAGAGAGAAGCTGCTCTTCTCCTGGTTATCCCCTTCCTTCTCCACCTACCTCAATCTCAC
PI 115816        CAGAGAGAAGCTGCTCTTCTCCTGGTTATCCCCTTCCTTCTCCACCTACCTCAATCTCAC
PI 283887        CAGAGAGAAGCTGCTCTTCTCCTGGTTATCCCCTTCCTTCTCCACCTACCTCAATCTCAC
PS5              CAGAGAGAAGCTGCTCTTCTCCTGGTTATCCCCTTCCTTCTCCACCTACCTCAATCTCAC
CItr 7779        CAGAGAGAAGCTGCTCTTCTCCTGGTTATCCCCTTCCTTCTCCACCTACCTCAATCTCAC
CItr 3686        CAGAGAGAAGCTGCTCTTCTCCTGGTTATCCCCTTCCTTCTCCACCTACCTCAATCTCAC
PI 113961        CAGAGAGAAGCTGCTCTTCTCCTGGTTATCCCCTTCCTTCTCCACCTACCTCAATCTCAC
```

```
PI 585017        CAGAGAGAAGCTGCTCTTCTCCTGGTTATCCCCTTCCTTCTCCACCTACCTCAATCTCAC
PI 532486        CAGAGAGAAGCTGCTCTTCTCCTGGTTATCCCCTTCCTTCTCCACCTACCTCAATCTCAC
PI 532475        CAGAGAGAAGCTGCTCTTCTCCTGGTTATCCCCTTCCTTCTCCACCTACCTCAATCTCAC
PI 470732        CAGAGAGAAGCTGCTCTTCTCCTGGTTATCCCCTTCCTTCTCCACCTACCTCAATCTCAC
PI 352281        CAGAGAGAAGCTGCTCTTCTCCTGGTTATCCCCTTCCTTCTCCACCTACCTCAATCTCAC
PI 349040        CAGAGAGAAGCTGCTCTTCTCCTGGTTATCCCCTTCCTTCTCCACCTACCTCAATCTCAC
PI 283889        CAGAGAGAAGCTGCTCTTCTCCTGGTTATCCCCTTCCTTCTCCACCTACCTCAATCTCAC
PI 251914        CAGAGAGAAGCTGCTCTTCTCCTGGTTATCCCCTTCCTTCTCCACCTACCTCAATCTCAC
PI 61102         CAGAGAGAAGCTGCTCTTCTCCTGGTTATCCCCTTCCTTCTCCACCTACCTCAATCTCAC
PI 352282        CAGAGAGAAGCTGCTCTTCTCCTGGTTATCCCCTTCCTTCTCCACCTACCTCAATCTCAC
PI 286070        CAGAGAGAAGCTGCTCTTCTCCTGGTTATCCCCTTCCTTCTCCACCTACCTCAATCTCAC
PI 115817        CAGAGAGAAGCTGCTCTTCTCCTGGTTATCCCCTTCCTTCTCCACCTACCTCAATCTCAC
PI 94749         CAGAGAGAAGCTGCTCTTCTCCTGGTTATCCCCTTCCTTCTCCACCTACCTCAATCTCAC
PI 94748         CAGAGAGAAGCTGCTCTTCTCCTGGTTATCCCCTTCCTTCTCCACCTACCTCAATCTCAC
                 ************************************************************

PI 499972        CTGCCCCCTTCCTCCTCCTCTCAGATCTGATCGATTAGGTAGATCGGGCCGTTACAAGTG
PI 532501        CTGCCCCCTTCCTCCTCCTCTCAGATCTGATCGATTAGGTAGATCGGGCCGTTACAAGTG
PI 352278        CTGCCCCCTTCCTCCTCCTCTCAGATCTGATCGATTAGGTAGATCGGGCCGTTACAAGTG
PI 115816        CTGCCCCCTTCCTCCTCCTCTCAGATCTGATCGATTAGGTAGATCGGGCCGTTACAAGTG
PI 283887        CTGCCCCCTTCCTCCTCCTCTCAGATCTGATCGATTAGGTAGATCGGGCCGTTACAAGTG
PS5              CTGCCCCCTTCCTCCTCCTCTCAGATCTGATCGATTAGGTAGATCGGGCCGTTACAAGTG
CItr 7779        CTGCCCCCTTCCTCCTCCTCTCAGATCTGATCGATTAGGTAGATCGGGCCGTTACAAGTG
CItr 3686        CTGCCCCCTTCCTCCTCCTCTCAGATCTGATCGATTAGGTAGATCGGGCCGTTACAAGTG
PI 113961        CTGCCCCCTTCCTCCTCCTCTCAGATCTGATCGATTAGGTAGATCGGGCCGTTACAAGTG
PI 585017        CTGCCCCCTTCCTCCTCCTCTCAGATCTGATCGATTAGGTAGATCGGGCCGTTACAAGTG
PI 532486        CTGCCCCCTTCCTCCTCCTCTCAGATCTGATCGATTAGGTAGATCGGGCCGTTACAAGTG
PI 532475        CTGCCCCCTTCCTCCTCCTCTCAGATCTGATCGATTAGGTAGATCGGGCCGTTACAAGTG
PI 470732        CTGCCCCCTTCCTCCTCCTCTCAGATCTGATCGATTAGGTAGATCGGGCCGTTACAAGTG
PI 352281        CTGCCCCCTTCCTCCTCCTCTCAGATCTGATCGATTAGGTAGATCGGGCCGTTACAAGTG
PI 349040        CTGCCCCCTTCCTCCTCCTCTCAGATCTGATCGATTAGGTAGATCGGGCCGTTACAAGTG
PI 283889        CTGCCCCCTTCCTCCTCCTCTCAGATCTGATCGATTAGGTAGATCGGGCCGTTACAAGTG
PI 251914        CTGCCCCCTTCCTCCTCCTCTCAGATCTGATCGATTAGGTAGATCGGGCCGTTACAAGTG
PI 61102         CTGCCCCCTTCCTCCTCCTCTCAGATCTGATCGATTAGGTAGATCGGGCCGTTACAAGTG
PI 352282        CTGCCCCCTTCCTCCTCCTCTCAGATCTGATCGATTAGGTAGATCGGGCCGTTACAAGTG
PI 286070        CTGCCCCCTTCCTCCTCCTCTCAGATCTGATCGATTAGGTAGATCGGGCCGTTACAAGTG
PI 115817        CTGCCCCCTTCCTCCTCCTCTCAGATCTGATCGATTAGGTAGATCGGGCCGTTACAAGTG
PI 94749         CTGCCCCCTTCCTCCTCCTCTCAGATCTGATCGATTAGGTAGATCGGGCCGTTACAAGTG
PI 94748         CTGCCCCCTTCCTCCTCCTCTCAGATCTGATCGATTAGGTAGATCGGGCCGTTACAAGTG
                 ************************************************************


PI 499972        GTATCAGAGGTCGGGCGATCTGGATAACCCGTACCGCAAGCCGCGCTACGCCACCAAGCA
PI 532501        GTATCAGAGGTCGGGCGATCTGGATAACCCGTACCGCAAGCCGCGCTACGCCACCAAGCA
PI 352278        GTATCAGAGGTCGGGCGATCTGGATAACCCGTACCGCAAGCCGCGCTACGCCACCAAGCA
PI 115816        GTATCAGAGGTCGGGCGATCTGGATAACCCGTACCGCAAGCCGCGCTACGCCACCAAGCA
PI 283887        GTATCAGAGGTCGGGCGATCTGGATAACCCGTACCGCAAGCCGCGCTACGCCACCAAGCA
PS5              GTATCAGAGGTCGGGCGATCTGGATAACCCGTACCGCAAGCCGCGCTACGCCACCAAGCA
CItr 7779        GTATCAGAGGTCGGGCGATCTGGATAACCCGTACCGCAAGCCGCGCTACGCCACCAAGCA
CItr 3686        GTATCAGAGGTCGGGCGATCTGGATAACCCGTACCGCAAGCCGCGCTACGCCACCAAGCA
PI 113961        GTATCAGAGGTCGGGCGATCTGGATAACCCGTACCGCAAGCCGCGCTACGCCACCAAGCA
PI 585017        GTATCAGAGGTCGGGCGATCTGGATAACCCGTACCGCAAGCCGCGCTACGCCACCAAGCA
PI 532486        GTATCAGAGGTCGGGCGATCTGGATAACCCGTACCGCAAGCCGCGCTACGCCACCAAGCA
PI 532475        GTATCAGAGGTCGGGCGATCTGGATAACCCGTACCGCAAGCCGCGCTACGCCACCAAGCA
PI 470732        GTATCAGAGGTCGGGCGATCTGGATAACCCGTACCGCAAGCCGCGCTACGCCACCAAGCA
PI 352281        GTATCAGAGGTCGGGCGATCTGGATAACCCGTACCGCAAGCCGCGCTACGCCACCAAGCA
```

```
PI 349040     GTATCAGAGGTCGGGCGATCTGGATAACCCGTACCGCAAGCCGCGCTACGCCACCAAGCA
PI 283889     GTATCAGAGGTCGGGCGATCTGGATAACCCGTACCGCAAGCCGCGCTACGCCACCAAGCA
PI 251914     GTATCAGAGGTCGGGCGATCTGGATAACCCGTACCGCAAGCCGCGCTACGCCACCAAGCA
PI 61102      GTATCAGAGGTCGGGCGATCTGGATAACCCGTACCGCAAGCCGCGCTACGCCACCAAGCA
PI 352282     GTATCAGAGGTCGGGCGATCTGGATAACCCGTACCGCAAGCCGCGCTACGCCACCAAGCA
PI 286070     GTATCAGAGGTCGGGCGATCTGGATAACCCGTACCGCAAGCCGCGCTACGCCACCAAGCA
PI 115817     GTATCAGAGGTCGGGCGATCTGGATAACCCGTACCGCAAGCCGCGCTACGCCACCAAGCA
PI 94749      GTATCAGAGGTCGGGCGATCTGGATAACCCGTACCGCAAGCCGCGCTACGCCACCAAGCA
PI 94748      GTATCAGAGGTCGGGCGATCTGGATAACCCGTACCGCAAGCCGCGCTACGCCACCAAGCA
              ************************************************************

PI 499972     AGTTACGCAAGCTGCAGCGATGGAGGAGCAATTGGCGGCGCTGGCCAAGGCGGTCAACGA
PI 532501     AGTTACGCAAGCTGCAGCGATGGAGGAGCAATTGGCGGCGCTGGCCAAGGCGGTCAACGA
PI 352278     AGTTACGCAAGCTGCAGCGATGGAGGAGCAATTGGCGGCGCTGGCCAAGGCGGTCAACGA
PI 115816     AGTTACGCAAGCTGCAGCGATGGAGGAGCAATTGGCGGCGCTGGCCAAGGCGGTCAACGA
PI 283887     AGTTACGCAAGCTGCAGCGATGGAGGAGCAATTGGCGGCGCTGGCCAAGGCGGTCAACGA
PS5           AGTTACGCAAGCTGCAGCGATGGAGGAGCAATTGGCGGCGCTGGCCAAGGCGGTCAACGA
CItr 7779     AGTTACGCAAGCTGCAGCGATGGAGGAGCAATTGGCGGCGCTGGCCAAGGCGGTCAACGA
CItr 3686     AGTTACGCAAGCTGCAGCGATGGAGGAGCAATTGGCGGCGCTGGCCAAGGCGGTCAACGA
PI 113961     AGTTACGCAAGCTGCAGCGATGGAGGAGCAATTGGCGGCGCTGGCCAAGGCGGTCAACGA
PI 585017     AGTTACGCAAGCTGCAGCGATGGAGGAGCAATTGGCGGCGCTGGCCAAGGCGGTCAACGA
PI 532486     AGTTACGCAAGCTGCAGCGATGGAGGAGCAATTGGCGGCGCTGGCCAAGGCGGTCAACGA
PI 532475     AGTTACGCAAGCTGCAGCGATGGAGGAGCAATTGGCGGCGCTGGCCAAGGCGGTCAACGA
PI 470732     AGTTACGCAAGCTGCAGCGATGGAGGAGCAATTGGCGGCGCTGGCCAAGGCGGTCAACGA
PI 352281     AGTTACGCAAGCTGCAGCGATGGAGGAGCAATTGGCGGCGCTGGCCAAGGCGGTCAACGA
PI 349040     AGTTACGCAAGCTGCAGCGATGGAGGAGCAATTGGCGGCGCTGGCCAAGGCGGTCAACGA
PI 283889     AGTTACGCAAGCTGCAGCGATGGAGGAGCAATTGGCGGCGCTGGCCAAGGCGGTCAACGA
PI 251914     AGTTACGCAAGCTGCAGCGATGGAGGAGCAATTGGCGGCGCTGGCCAAGGCGGTCAACGA
PI 61102      AGTTACGCAAGCTGCAGCGATGGAGGAGCAATTGGCGGCGCTGGCCAAGGCGGTCAACGA
PI 352282     AGTTACGCAAGCTGCAGCGATGGAGGAGCAATTGGCGGCGCTGGCCAAGGCGGTCAACGA
PI 286070     AGTTACGCAAGCTGCAGCGATGGAGGAGCAATTGGCGGCGCTGGCCAAGGCGGTCAACGA
PI 115817     AGTTACGCAAGCTGCAGCGATGGAGGAGCAATTGGCGGCGCTGGCCAAGGCGGTCAACGA
PI 94749      AGTTACGCAAGCTGCAGCGATGGAGGAGCAATTGGCGGCGCTGGCCAAGGCGGTCAACGA
PI 94748      AGTTACGCAAGCTGCAGCGATGGAGGAGCAATTGGCGGCGCTGGCCAAGGCGGTCAACGA
              ************************************************************


PI 499972     CGGCCGCACCGCCAACGAGGCCCGGCTCGAGGCCATCCAGACCTCGCTCGAGTTGTGGCG
PI 532501     CGGCCGCACCGCCAACGAGGCCCGGCTCGAGGCCATCCAGACCTCGCTCGAGTTGTGGCG
PI 352278     CGGCCGCACCGCCAACGAGGCCCGGCTCGAGGCCATCCAGACCTCGCTCGAGTTGTGGCG
PI 115816     CGGCCGCACCGCCAACGAGGCCCGGCTCGAGGCCATCCAGACCTCGCTCGAGTTGTGGCG
PI 283887     CGGCCGCACCGCCAACGAGGCCCGGCTCGAGGCCATCCAGACCTCGCTCGAGTTGTGGCG
PS5           CGGCCGCACCGCCAACGAGGCCCGGCTCGAGGCCATCCAGACCTCGCTCGAGTTGTGGCG
CItr 7779     CGGCCGCACCGCCAACGAGGCCCGGCTCGAGGCCATCCAGACCTCGCTCGAGTTGTGGCG
CItr 3686     CGGCCGCACCGCCAACGAGGCCCGGCTCGAGGCCATCCAGACCTCGCTCGAGTTGTGGCG
PI 113961     CGGCCGCACCGCCAACGAGGCCCGGCTCGAGGCCATCCAGACCTCGCTCGAGTTGTGGCG
PI 585017     CGGCCGCACCGCCAACGAGGCCCGGCTCGAGGCCATCCAGACCTCGCTCGAGTTGTGGCG
PI 532486     CGGCCGCACCGCCAACGAGGCCCGGCTCGAGGCCATCCAGACCTCGCTCGAGTTGTGGCG
PI 532475     CGGCCGCACCGCCAACGAGGCCCGGCTCGAGGCCATCCAGACCTCGCTCGAGTTGTGGCG
PI 470732     CGGCCGCACCGCCAACGAGGCCCGGCTCGAGGCCATCCAGACCTCGCTCGAGTTGTGGCG
PI 352281     CGGCCGCACCGCCAACGAGGCCCGGCTCGAGGCCATCCAGACCTCGCTCGAGTTGTGGCG
PI 349040     CGGCCGCACCGCCAACGAGGCCCGGCTCGAGGCCATCCAGACCTCGCTCGAGTTGTGGCG
PI 283889     CGGCCGCACCGCCAACGAGGCCCGGCTCGAGGCCATCCAGACCTCGCTCGAGTTGTGGCG
PI 251914     CGGCCGCACCGCCAACGAGGCCCGGCTCGAGGCCATCCAGACCTCGCTCGAGTTGTGGCG
PI 61102      CGGCCGCACCGCCAACGAGGCCCGGCTCGAGGCCATCCAGACCTCGCTCGAGTTGTGGCG
PI 352282     CGGCCGCACCGCCAACGAGGCCCGGCTCGAGGCCATCCAGACCTCGCTCGAGTTGTGGCG
```

```
PI 286070        CGGCCGCACCGCCAACGAGGCCCGGCTCGAGGCCATCCAGACCTCGCTCGAGTTGTGGCG
PI 115817        CGGCCGCACCGCCAACGAGGCCCGGCTCGAGGCCATCCAGACCTCGCTCGAGTTGTGGCG
PI 94749         CGGCCGCACCGCCAACGAGGCCCGGCTCGAGGCCATCCAGACCTCGCTCGAGTTGTGGCG
PI 94748         CGGCCGCACCGCCAACGAGGCCCGGCTCGAGGCCATCCAGACCTCGCTCGAGTTGTGGCG
                 ************************************************************

PI 499972        TCCAGCGGTCACCAACCTGCAACAACAACTCAACGAGCTCCAATCCCAAGTGGGGCGGAT
PI 532501        TCCAGCGGTCACCAACCTGCAACAACAACTCAACGAGCTCCAATCCCAAGTGGGGCGGAT
PI 352278        TCCAGCGGTCACCAACCTGCAACAACAACTCAACGAGCTCCAATCCCAAGTGGGGCGGAT
PI 115816        TCCAGCGGTCACCAACCTGCAACAACAACTCAACGAGCTCCAATCCCAAGTGGGGCGGAT
PI 283887        TCCAGCGGTCACCAACCTGCAACAACAACTCAACGAGCTCCAATCCCAAGTGGGGCGGAT
PS5              TCCAGCGGTCACCAACCTGCAACAACAACTCAACGAGCTCCAATCCCAAGTGGGGCGGAT
CItr 7779        TCCAGCGGTCACCAACCTGCAACAACAACTCAACGAGCTCCAATCCCAAGTGGGGCGGAT
CItr 3686        TCCAGCGGTCACCAACCTGCAACAACAACTCAACGAGCTCCAATCCCAAGTGGGGCGGAT
PI 113961        TCCAGCGGTCACCAACCTGCAACAACAACTCAACGAGCTCCAATCCCAAGTGGGGCGGAT
PI 585017        TCCAGCGGTCACCAACCTGCAACAACAACTCAACGAGCTCCAATCCCAAGTGGGGCGGAT
PI 532486        TCCAGCGGTCACCAACCTGCAACAACAACTCAACGAGCTCCAATCCCAAGTGGGGCGGAT
PI 532475        TCCAGCGGTCACCAACCTGCAACAACAACTCAACGAGCTCCAATCCCAAGTGGGGCGGAT
PI 470732        TCCAGCGGTCACCAACCTGCAACAACAACTCAACGAGCTCCAATCCCAAGTGGGGCGGAT
PI 352281        TCCAGCGGTCACCAACCTGCAACAACAACTCAACGAGCTCCAATCCCAAGTGGGGCGGAT
PI 349040        TCCAGCGGTCACCAACCTGCAACAACAACTCAACGAGCTCCAATCCCAAGTGGGGCGGAT
PI 283889        TCCAGCGGTCACCAACCTGCAACAACAACTCAACGAGCTCCAATCCCAAGTGGGGCGGAT
PI 251914        TCCAGCGGTCACCAACCTGCAACAACAACTCAACGAGCTCCAATCCCAAGTGGGGCGGAT
PI 61102         TCCAGCGGTCACCAACCTGCAACAACAACTCAACGAGCTCCAATCCCAAGTGGGGCGGAT
PI 352282        TCCAGCGGTCACCAACCTGCAACAACAACTCAACGAGCTCCAATCCCAAGTGGGGCGGAT
PI 286070        TCCAGCGGTCACCAACCTGCAACAACAACTCAACGAGCTCCAATCCCAAGTGGGGCGGAT
PI 115817        TCCAGCGGTCACCAACCTGCAACAACAACTCAACGAGCTCCAATCCCAAGTGGGGCGGAT
PI 94749         TCCAGCGGTCACCAACCTGCAACAACAACTCAACGAGCTCCAATCCCAAGTGGGGCGGAT
PI 94748         TCCAGCGGTCACCAACCTGCAACAACAACTCAACGAGCTCCAATCCCAAGTGGGGCGGAT
                 ************************************************************
```

Comparison of 615-bp sequence amplified by primers VBINS5F (5’-CAACCAGAGGCAATTCTGGACAC-3’) and VRNBPR1 (5’-GCCCCATCTCCGCTGGAGAACG-3’):

```
PI 499972      CAACCAGAGGCAATTCTGGACACCAAGTTCATCCGCGTCCGGGGAGAGATGCAACCTCGT
CItr 7779      CAACCAGAGGCAATTCTGGACACCAAGTTCATCCGCGTCCGGGGAGAGATGCAACCTCGT
PI 352281      CAACCAGAGGCAATTCTGGACACCAAGTTCATCCGCGTCCGGGGAGAGATGCAACCTCGT
PI 286070      CAACCAGAGGCAATTCTGGACACCAAGTTCATCCGCGTCCGGGGAGAGATGCAACCTCGT
PI 94748       CAACCAGAGGCAATTCTGGACACCAAGTTCATCCGCGTCCGGGGAGAGATGCAACCTCGT
PS5            CAACCAGAGGCAATTCTGGACACCAAGTTCATCCGCGTCCGGGGAGAGATGCAACCTCGT
PI 532501      CAACCAGAGGCAATTCTGGACACCAAGTTCATCCGCGTCCGGGGAGAGATGCAACCTCGT
PI 532475      CAACCAGAGGCAATTCTGGACACCAAGTTCATCCGCGTCCGGGGAGAGATGCAACCTCGT
PI 470732      CAACCAGAGGCAATTCTGGACACCAAGTTCATCCGCGTCCGGGGAGAGATGCAACCTCGT
PI 349040      CAACCAGAGGCAATTCTGGACACCAAGTTCATCCGCGTCCGGGGAGAGATGCAACCTCGT
PI 113961      CAACCAGAGGCAATTCTGGACACCAAGTTCATCCGCGTCCGGGGAGAGATGCAACCTCGT
CItr 3686      CAACCAGAGGCAATTCTGGACACCAAGTTCATCCGCGTCCGGGGAGAGATGCAACCTCGT
PI 585017      CAACCAGAGGCAATTCTGGACACCAAGTTCATCCGCGTCCGGGGAGAGATGCAACCTCGT
PI 532486      CAACCAGAGGCAATTCTGGACACCAAGTTCATCCGCGTCCGGGGAGAGATGCAACCTCGT
PI 352282      CAACCAGAGGCAATTCTGGACACCAAGTTCATCCGCGTCCGGGGAGAGATGCAACCTCGT
PI 352278      CAACCAGAGGCAATTCTGGACACCAAGTTCATCCGCGTCCGGGGAGAGATGCAACCTCGT
PI 283889      CAACCAGAGGCAATTCTGGACACCAAGTTCATCCGCGTCCGGGGAGAGATGCAACCTCGT
PI 251914      CAACCAGAGGCAATTCTGGACACCAAGTTCATCCGCGTCCGGGGAGAGATGCAACCTCGT
PI 115816      CAACCAGAGGCAATTCTGGACACCAAGTTCATCCGCGTCCGGGGAGAGATGCAACCTCGT
PI 61102       CAACCAGAGGCAATTCTGGACACCAAGTTCATCCGCGTCCGGGGAGAGATGCAACCTCGT
PI 283887      CAACCAGAGGCAATTCTGGACACCAAGTTCATCCGCGTCCGGGGAGAGATGCAACCTCGT
PI 94749       CAACCAGAGGCAATTCTGGACACCAAGTTCATCCGCGTCCGGGGAGAGATGCAACCTCGT
PI 115817      CAACCAGAGGCAATTCTGGACACCAAGTTCATCCGCGTCCGGGGAGAGATGCAACCTCGT
               ************************************************************

PI 499972      TCTTCTGGTACAATGGGGTGCGCTGCCGGCGTCGCTCGCCACCTGGGAGGAGCCCGCTGA
CItr 7779      TCTTCTGGTACAATGGGGTGCGCTGCCGGCGTCGCTCGCCACCTGGGAGGAGCCCGCTGA
PI 352281      TCTTCTGGTACAATGGGGTGCGCTGCCGGCGTCGCTCGCCACCTGGGAGGAGCCCGCTGA
PI 286070      TCTTCTGGTACAATGGGGTGCGCTGCCGGCGTCGCTCGCCACCTGGGAGGAGCCCGCTGA
PI 94748       TCTTCTGGTACAATGGGGTGCGCTGCCGGCGTCGCTCGCCACCTGGGAGGAGCCCGCTGA
PS5            TCTTCTGGTACAATGGGGTGCGCTGCCGGCGTCGCTCGCCACCTGGGAGGAGCCCGCTGA
PI 532501      TCTTCTGGTACAATGGGGTGCGCTGCCGGCGTCGCTCGCCACCTGGGAGGAGCCCGCTGA
PI 532475      TCTTCTGGTACAATGGGGTGCGCTGCCGGCGTCGCTCGCCACCTGGGAGGAGCCCGCTGA
PI 470732      TCTTCTGGTACAATGGGGTGCGCTGCCGGCGTCGCTCGCCACCTGGGAGGAGCCCGCTGA
PI 349040      TCTTCTGGTACAATGGGGTGCGCTGCCGGCGTCGCTCGCCACCTGGGAGGAGCCCGCTGA
PI 113961      TCTTCTGGTACAATGGGGTGCGCTGCCGGCGTCGCTCGCCACCTGGGAGGAGCCCGCTGA
CItr 3686      TCTTCTGGTACAATGGGGTGCGCTGCCGGCGTCGCTCGCCACCTGGGAGGAGCCCGCTGA
PI 585017      TCTTCTGGTACAATGGGGTGCGCTGCCGGCGTCGCTCGCCACCTGGGAGGAGCCCGCTGA
PI 532486      TCTTCTGGTACAATGGGGTGCGCTGCCGGCGTCGCTCGCCACCTGGGAGGAGCCCGCTGA
PI 352282      TCTTCTGGTACAATGGGGTGCGCTGCCGGCGTCGCTCGCCACCTGGGAGGAGCCCGCTGA
PI 352278      TCTTCTGGTACAATGGGGTGCGCTGCCGGCGTCGCTCGCCACCTGGGAGGAGCCCGCTGA
PI 283889      TCTTCTGGTACAATGGGGTGCGCTGCCGGCGTCGCTCGCCACCTGGGAGGAGCCCGCTGA
PI 251914      TCTTCTGGTACAATGGGGTGCGCTGCCGGCGTCGCTCGCCACCTGGGAGGAGCCCGCTGA
PI 115816      TCTTCTGGTACAATGGGGTGCGCTGCCGGCGTCGCTCGCCACCTGGGAGGAGCCCGCTGA
PI 61102       TCTTCTGGTACAATGGGGTGCGCTGCCGGCGTCGCTCGCCACCTGGGAGGAGCCCGCTGA
PI 283887      TCTTCTGGTACAATGGGGTGCGCTGCCGGCGTCGCTCGCCACCTGGGAGGAGCCCGCTGA
PI 94749       TCTTCTGGTACAATGGGGTGCGCTGCCGGCGTCGCTCGCCACCTGGGAGGAGCCCGCTGA
PI 115817      TCTTCTGGTACAATGGGGTGCGCTGCCGGCGTCGCTCGCCACCTGGGAGGAGCCCGCTGA
               ************************************************************

PI 499972      ACTGCGTCGTCGCTTTCCAGCAGCATCGGCTTGGGGACAAGCCTCACCTCAAGGGGGGGA
CItr 7779      ACTGCGTCGTCGCTTTCCAGCAGCATCGGCTTGGGGACAAGCCTCACCTCAAGGGGGGGA
PI 352281      ACTGCGTCGTCGCTTTCCAGCAGCATCGGCTTGGGGACAAGCCTCACCTCAAGGGGGGGA
PI 286070      ACTGCGTCGTCGCTTTCCAGCAGCATCGGCTTGGGGACAAGCCTCACCTCAAGGGGGGGA
```

```
PI 94748        ACTGCGTCGTCGCTTTCCAGCAGCATCGGCTTGGGGACAAGCCTCACCTCAAGGGGGGGA
PS5             ACTGCGTCGTCGCTTTCCAGCAGCATCGGCTTGGGGACAAGCCTCACCTCAAGGGGGGGA
PI 532501       ACTGCGTCGTCGCTTTCCAGCAGCATCGGCTTGGGGACAAGCCTCACCTCAAGGGGGGGA
PI 532475       ACTGCGTCGTCGCTTTCCAGCAGCATCGGCTTGGGGACAAGCCTCACCTCAAGGGGGGGA
PI 470732       ACTGCGTCGTCGCTTTCCAGCAGCATCGGCTTGGGGACAAGCCTCACCTCAAGGGGGGGA
PI 349040       ACTGCGTCGTCGCTTTCCAGCAGCATCGGCTTGGGGACAAGCCTCACCTCAAGGGGGGGA
PI 113961       ACTGCGTCGTCGCTTTCCAGCAGCATCGGCTTGGGGACAAGCCTCACCTCAAGGGGGGGA
CItr 3686       ACTGCGTCGTCGCTTTCCAGCAGCATCGGCTTGGGGACAAGCCTCACCTCAAGGGGGGGA
PI 585017       ACTGCGTCGTCGCTTTCCAGCAGCATCGGCTTGGGGACAAGCCTCACCTCAAGGGGGGGA
PI 532486       ACTGCGTCGTCGCTTTCCAGCAGCATCGGCTTGGGGACAAGCCTCACCTCAAGGGGGGGA
PI 352282       ACTGCGTCGTCGCTTTCCAGCAGCATCGGCTTGGGGACAAGCCTCACCTCAAGGGGGGGA
PI 352278       ACTGCGTCGTCGCTTTCCAGCAGCATCGGCTTGGGGACAAGCCTCACCTCAAGGGGGGGA
PI 283889       ACTGCGTCGTCGCTTTCCAGCAGCATCGGCTTGGGGACAAGCCTCACCTCAAGGGGGGGA
PI 251914       ACTGCGTCGTCGCTTTCCAGCAGCATCGGCTTGGGGACAAGCCTCACCTCAAGGGGGGGA
PI 115816       ACTGCGTCGTCGCTTTCCAGCAGCATCGGCTTGGGGACAAGCCTCACCTCAAGGGGGGGA
PI 61102        ACTGCGTCGTCGCTTTCCAGCAGCATCGGCTTGGGGACAAGCCTCACCTCAAGGGGGGGA
PI 283887       ACTGCGTCGTCGCTTTCCAGCAGCATCGGCTTGGGGACAAGCCTCACCTCAAGGGGGGGA
PI 94749        ACTGCGTCGTCGCTTTCCAGCAGCATCGGCTTGGGGACAAGCCTCACCTCAAGGGGGGGA
PI 115817       ACTGCGTCGTCGCTTTCCAGCAGCATCGGCTTGGGGACAAGCCTCACCTCAAGGGGGGGA
                ************************************************************

PI 499972       GGATGTCACGACTGTCAAGGTGACCTCACCAGCCAACAAGCGGGAAGAGGAACTGGTCAA
CItr 7779       GGATGTCACGACTGTCAAGGTGACCTCACCAGCCAACAAGCGGGAAGAGGAACTGGTCAA
PI 352281       GGATGTCACGACTGTCAAGGTGACCTCACCAGCCAACAAGCGGGAAGAGGAACTGGTCAA
PI 286070       GGATGTCACGACTGTCAAGGTGACCTCACCAGCCAACAAGCGGGAAGAGGAACTGGTCAA
PI 94748        GGATGTCACGACTGTCAAGGTGACCTCACCAGCCAACAAGCGGGAAGAGGAACTGGTCAA
PS5             GGATGTCACGACTGTCAAGGTGACCTCACCAGCCAACAAGCGGGAAGAGGAACTGGTCAA
PI 532501       GGATGTCACGACTGTCAAGGTGACCTCACCAGCCAACAAGCGGGAAGAGGAACTGGTCAA
PI 532475       GGATGTCACGACTGTCAAGGTGACCTCACCAGCCAACAAGCGGGAAGAGGAACTGGTCAA
PI 470732       GGATGTCACGACTGTCAAGGTGACCTCACCAGCCAACAAGCGGGAAGAGGAACTGGTCAA
PI 349040       GGATGTCACGACTGTCAAGGTGACCTCACCAGCCAACAAGCGGGAAGAGGAACTGGTCAA
PI 113961       GGATGTCACGACTGTCAAGGTGACCTCACCAGCCAACAAGCGGGAAGAGGAACTGGTCAA
CItr 3686       GGATGTCACGACTGTCAAGGTGACCTCACCAGCCAACAAGCGGGAAGAGGAACTGGTCAA
PI 585017       GGATGTCACGACTGTCAAGGTGACCTCACCAGCCAACAAGCGGGAAGAGGAACTGGTCAA
PI 532486       GGATGTCACGACTGTCAAGGTGACCTCACCAGCCAACAAGCGGGAAGAGGAACTGGTCAA
PI 352282       GGATGTCACGACTGTCAAGGTGACCTCACCAGCCAACAAGCGGGAAGAGGAACTGGTCAA
PI 352278       GGATGTCACGACTGTCAAGGTGACCTCACCAGCCAACAAGCGGGAAGAGGAACTGGTCAA
PI 283889       GGATGTCACGACTGTCAAGGTGACCTCACCAGCCAACAAGCGGGAAGAGGAACTGGTCAA
PI 251914       GGATGTCACGACTGTCAAGGTGACCTCACCAGCCAACAAGCGGGAAGAGGAACTGGTCAA
PI 115816       GGATGTCACGACTGTCAAGGTGACCTCACCAGCCAACAAGCGGGAAGAGGAACTGGTCAA
PI 61102        GGATGTCACGACTGTCAAGGTGACCTCACCAGCCAACAAGCGGGAAGAGGAACTGGTCAA
PI 283887       GGATGTCACGACTGTCAAGGTGACCTCACCAGCCAACAAGCGGGAAGAGGAACTGGTCAA
PI 94749        GGATGTCACGACTGTCAAGGTGACCTCACCAGCCAACAAGCGGGAAGAGGAACTGGTCAA
PI 115817       GGATGTCACGACTGTCAAGGTGACCTCACCAGCCAACAAGCGGGAAGAGGAACTGGTCAA
                ************************************************************

PI 499972       GGCGCTTGCTGATTCGACAGAAGGGCCGACCCGTTAGCAGGCCGCGGTGTCTTGGGTGCG
CItr 7779       GGCGCTTGCTGATTCGACAGAAGGGCCGACCCGTTAGCAGGCCGCGGTGTCTTGGGTGCG
PI 352281       GGCGCTTGCTGATTCGACAGAAGGGCCGACCCGTTAGCAGGCCGCGGTGTCTTGGGTGCG
PI 286070       GGCGCTTGCTGATTCGACAGAAGGGCCGACCCGTTAGCAGGCCGCGGTGTCTTGGGTGCG
PI 94748        GGCGCTTGCTGATTCGACAGAAGGGCCGACCCGTTAGCAGGCCGCGGTGTCTTGGGTGCG
PS5             GGCGCTTGCTGATTCGACAGAAGGGCCGACCCGTTAGCAGGCCGCGGTGTCTTGGGTGCG
PI 532501       GGCGCTTGCTGATTCGACAGAAGGGCCGACCCGTTAGCAGGCCGCGGTGTCTTGGGTGCG
PI 532475       GGCGCTTGCTGATTCGACAGAAGGGCCGACCCGTTAGCAGGCCGCGGTGTCTTGGGTGCG
PI 470732       GGCGCTTGCTGATTCGACAGAAGGGCCGACCCGTTAGCAGGCCGCGGTGTCTTGGGTGCG
```

```
PI 349040       GGCGCTTGCTGATTCGACAGAAGGGCCGACCCGTTAGCAGGCCGCGGTGTCTTGGGTGCG
PI 113961       GGCGCTTGCTGATTCGACAGAAGGGCCGACCCGTTAGCAGGCCGCGGTGTCTTGGGTGCG
CItr 3686       GGCGCTTGCTGATTCGACAGAAGGGCCGACCCGTTAGCAGGCCGCGGTGTCTTGGGTGCG
PI 585017       GGCGCTTGCTGATTCGACAGAAGGGCCGACCCGTTAGCAGGCCGCGGTGTCTTGGGTGCG
PI 532486       GGCGCTTGCTGATTCGACAGAAGGGCCGACCCGTTAGCAGGCCGCGGTGTCTTGGGTGCG
PI 352282       GGCGCTTGCTGATTCGACAGAAGGGCCGACCCGTTAGCAGGCCGCGGTGTCTTGGGTGCG
PI 352278       GGCGCTTGCTGATTCGACAGAAGGGCCGACCCGTTAGCAGGCCGCGGTGTCTTGGGTGCG
PI 283889       GGCGCTTGCTGATTCGACAGAAGGGCCGACCCGTTAGCAGGCCGCGGTGTCTTGGGTGCG
PI 251914       GGCGCTTGCTGATTCGACAGAAGGGCCGACCCGTTAGCAGGCCGCGGTGTCTTGGGTGCG
PI 115816       GGCGCTTGCTGATTCGACAGAAGGGCCGACCCGTTAGCAGGCCGCGGTGTCTTGGGTGCG
PI 61102        GGCGCTTGCTGATTCGACAGAAGGGCCGACCCGTTAGCAGGCCGCGGTGTCTTGGGTGCG
PI 283887       GGCGCTTGCTGATTCGACAGAAGGGCCGACCCGTTAGCAGGCCGCGGTGTCTTGGGTGCG
PI 94749        GGCGCTTGCTGATTCGACAGAAGGGCCGACCCGTTAGCAGGCCGCGGTGTCTTGGGTGCG
PI 115817       GGCGCTTGCTGATTCGACAGAAGGGCCGACCCGTTAGCAGGCCGCGGTGTCTTGGGTGCG
                ************************************************************

PI 499972       TGGCCTTGTGGGCCGTATCGAGCCTGCGAGCTCAGGGTTAAGTAAGTGGCTTGGCACCGT
CItr 7779       TGGCCTTGTGGGCCGTATCGAGCCTGCGAGCTCAGGGTTAAGTAAGTGGCTTGGCACCGT
PI 352281       TGGCCTTGTGGGCCGTATCGAGCCTGCGAGCTCAGGGTTAAGTAAGTGGCTTGGCACCGT
PI 286070       TGGCCTTGTGGGCCGTATCGAGCCTGCGAGCTCAGGGTTAAGTAAGTGGCTTGGCACCGT
PI 94748        TGGCCTTGTGGGCCGTATCGAGCCTGCGAGCTCAGGGTTAAGTAAGTGGCTTGGCACCGT
PS5             TGGCCTTGTGGGCCGTATCGAGCCTGCGAGCTCAGGGTTAAGTAAGTGGCTTGGCACCGT
PI 532501       TGGCCTTGTGGGCCGTATCGAGCCTGCGAGCTCAGGGTTAAGTAAGTGGCTTGGCACCGT
PI 532475       TGGCCTTGTGGGCCGTATCGAGCCTGCGAGCTCAGGGTTAAGTAAGTGGCTTGGCACCGT
PI 470732       TGGCCTTGTGGGCCGTATCGAGCCTGCGAGCTCAGGGTTAAGTAAGTGGCTTGGCACCGT
PI 349040       TGGCCTTGTGGGCCGTATCGAGCCTGCGAGCTCAGGGTTAAGTAAGTGGCTTGGCACCGT
PI 113961       TGGCCTTGTGGGCCGTATCGAGCCTGCGAGCTCAGGGTTAAGTAAGTGGCTTGGCACCGT
CItr 3686       TGGCCTTGTGGGCCGTATCGAGCCTGCGAGCTCAGGGTTAAGTAAGTGGCTTGGCACCGT
PI 585017       TGGCCTTGTGGGCCGTATCGAGCCTGCGAGCTCAGGGTTAAGTAAGTGGCTTGGCACCGT
PI 532486       TGGCCTTGTGGGCCGTATCGAGCCTGCGAGCTCAGGGTTAAGTAAGTGGCTTGGCACCGT
PI 352282       TGGCCTTGTGGGCCGTATCGAGCCTGCGAGCTCAGGGTTAAGTAAGTGGCTTGGCACCGT
PI 352278       TGGCCTTGTGGGCCGTATCGAGCCTGCGAGCTCAGGGTTAAGTAAGTGGCTTGGCACCGT
PI 283889       TGGCCTTGTGGGCCGTATCGAGCCTGCGAGCTCAGGGTTAAGTAAGTGGCTTGGCACCGT
PI 251914       TGGCCTTGTGGGCCGTATCGAGCCTGCGAGCTCAGGGTTAAGTAAGTGGCTTGGCACCGT
PI 115816       TGGCCTTGTGGGCCGTATCGAGCCTGCGAGCTCAGGGTTAAGTAAGTGGCTTGGCACCGT
PI 61102        TGGCCTTGTGGGCCGTATCGAGCCTGCGAGCTCAGGGTTAAGTAAGTGGCTTGGCACCGT
PI 283887       TGGCCTTGTGGGCCGTATCGAGCCTGCGAGCTCAGGGTTAAGTAAGTGGCTTGGCACCGT
PI 94749        TGGCCTTGTGGGCCGTATCGAGCCTGCGAGCTCAGGGTTAAGTAAGTGGCTTGGCACCGT
PI 115817       TGGCCTTGTGGGCCGTATCGAGCCTGCGAGCTCAGGGTTAAGTAAGTGGCTTGGCACCGT
                ************************************************************


PI 499972       CAAGGGCATCAAATTGTATCTCACTGAATAGACAGAGAGAAGCTGCTCTTCTCCTGGTTA
CItr 7779       CAAGGGCATCAAATTGTATCTCACTGAATAGACAGAGAGAAGCTGCTCTTCTCCTGGTTA
PI 352281       CAAGGGCATCAAATTGTATCTCACTGAATAGACAGAGAGAAGCTGCTCTTCTCCTGGTTA
PI 286070       CAAGGGCATCAAATTGTATCTCACTGAATAGACAGAGAGAAGCTGCTCTTCTCCTGGTTA
PI 94748        CAAGGGCATCAAATTGTATCTCACTGAATAGACAGAGAGAAGCTGCTCTTCTCCTGGTTA
PS5             CAAGGGCATCAAATTGTATCTCACTGAATAGACAGAGAGAAGCTGCTCTTCTCCTGGTTA
PI 532501       CAAGGGCATCAAATTGTATCTCACTGAATAGACAGAGAGAAGCTGCTCTTCTCCTGGTTA
PI 532475       CAAGGGCATCAAATTGTATCTCACTGAATAGACAGAGAGAAGCTGCTCTTCTCCTGGTTA
PI 470732       CAAGGGCATCAAATTGTATCTCACTGAATAGACAGAGAGAAGCTGCTCTTCTCCTGGTTA
PI 349040       CAAGGGCATCAAATTGTATCTCACTGAATAGACAGAGAGAAGCTGCTCTTCTCCTGGTTA
PI 113961       CAAGGGCATCAAATTGTATCTCACTGAATAGACAGAGAGAAGCTGCTCTTCTCCTGGTTA
CItr 3686       CAAGGGCATCAAATTGTATCTCACTGAATAGACAGAGAGAAGCTGCTCTTCTCCTGGTTA
PI 585017       CAAGGGCATCAAATTGTATCTCACTGAATAGACAGAGAGAAGCTGCTCTTCTCCTGGTTA
PI 532486       CAAGGGCATCAAATTGTATCTCACTGAATAGACAGAGAGAAGCTGCTCTTCTCCTGGTTA
```

```
PI 352282       CAAGGGCATCAAATTGTATCTCACTGAATAGACAGAGAGAAGCTGCTCTTCTCCTGGTTA
PI 352278       CAAGGGCATCAAATTGTATCTCACTGAATAGACAGAGAGAAGCTGCTCTTCTCCTGGTTA
PI 283889       CAAGGGCATCAAATTGTATCTCACTGAATAGACAGAGAGAAGCTGCTCTTCTCCTGGTTA
PI 251914       CAAGGGCATCAAATTGTATCTCACTGAATAGACAGAGAGAAGCTGCTCTTCTCCTGGTTA
PI 115816       CAAGGGCATCAAATTGTATCTCACTGAATAGACAGAGAGAAGCTGCTCTTCTCCTGGTTA
PI 61102        CAAGGGCATCAAATTGTATCTCACTGAATAGACAGAGAGAAGCTGCTCTTCTCCTGGTTA
PI 283887       CAAGGGCATCAAATTGTATCTCACTGAATAGACAGAGAGAAGCTGCTCTTCTCCTGGTTA
PI 94749        CAAGGGCATCAAATTGTATCTCACTGAATAGACAGAGAGAAGCTGCTCTTCTCCTGGTTA
PI 115817       CAAGGGCATCAAATTGTATCTCACTGAATAGACAGAGAGAAGCTGCTCTTCTCCTGGTTA
                ************************************************************

PI 499972       TCCCCTTCCTTCTCCACCTACCTCAATCTCACCTGCCCCCTTCCTCCTCCTCTCAGATCT
CItr 7779       TCCCCTTCCTTCTCCACCTACCTCAATCTCACCTGCCCCCTTCCTCCTCCTCTCAGATCT
PI 352281       TCCCCTTCCTTCTCCACCTACCTCAATCTCACCTGCCCCCTTCCTCCTCCTCTCAGATCT
PI 286070       TCCCCTTCCTTCTCCACCTACCTCAATCTCACCTGCCCCCTTCCTCCTCCTCTCAGATCT
PI 94748        TCCCCTTCCTTCTCCACCTACCTCAATCTCACCTGCCCCCTTCCTCCTCCTCTCAGATCT
PS5             TCCCCTTCCTTCTCCACCTACCTCAATCTCACCTGCCCCCTTCCTCCTCCTCTCAGATCT
PI 532501       TCCCCTTCCTTCTCCACCTACCTCAATCTCACCTGCCCCCTTCCTCCTCCTCTCAGATCT
PI 532475       TCCCCTTCCTTCTCCACCTACCTCAATCTCACCTGCCCCCTTCCTCCTCCTCTCAGATCT
PI 470732       TCCCCTTCCTTCTCCACCTACCTCAATCTCACCTGCCCCCTTCCTCCTCCTCTCAGATCT
PI 349040       TCCCCTTCCTTCTCCACCTACCTCAATCTCACCTGCCCCCTTCCTCCTCCTCTCAGATCT
PI 113961       TCCCCTTCCTTCTCCACCTACCTCAATCTCACCTGCCCCCTTCCTCCTCCTCTCAGATCT
CItr 3686       TCCCCTTCCTTCTCCACCTACCTCAATCTCACCTGCCCCCTTCCTCCTCCTCTCAGATCT
PI 585017       TCCCCTTCCTTCTCCACCTACCTCAATCTCACCTGCCCCCTTCCTCCTCCTCTCAGATCT
PI 532486       TCCCCTTCCTTCTCCACCTACCTCAATCTCACCTGCCCCCTTCCTCCTCCTCTCAGATCT
PI 352282       TCCCCTTCCTTCTCCACCTACCTCAATCTCACCTGCCCCCTTCCTCCTCCTCTCAGATCT
PI 352278       TCCCCTTCCTTCTCCACCTACCTCAATCTCACCTGCCCCCTTCCTCCTCCTCTCAGATCT
PI 283889       TCCCCTTCCTTCTCCACCTACCTCAATCTCACCTGCCCCCTTCCTCCTCCTCTCAGATCT
PI 251914       TCCCCTTCCTTCTCCACCTACCTCAATCTCACCTGCCCCCTTCCTCCTCCTCTCAGATCT
PI 115816       TCCCCTTCCTTCTCCACCTACCTCAATCTCACCTGCCCCCTTCCTCCTCCTCTCAGATCT
PI 61102        TCCCCTTCCTTCTCCACCTACCTCAATCTCACCTGCCCCCTTCCTCCTCCTCTCAGATCT
PI 283887       TCCCCTTCCTTCTCCACCTACCTCAATCTCACCTGCCCCCTTCCTCCTCCTCTCAGATCT
PI 94749        TCCCCTTCCTTCTCCACCTACCTCAATCTCACCTGCCCCCTTCCTCCTCCTCTCAGATCT
PI 115817       TCCCCTTCCTTCTCCACCTACCTCAATCTCACCTGCCCCCTTCCTCCTCCTCTCAGATCT
                ************************************************************


PI 499972       GATCGATTAGGTAGATCGGGCCGTTACACTCCGCCCCCCCGCCCCCGCCTGCGCCTGTCG
CItr 7779       GATCGATTAGGTAGATCGGGCCGTTACACTCCGCCCCCCCGCCCCCGCCTGCGCCTGTCG
PI 352281       GATCGATTAGGTAGATCGGGCCGTTACACTCCGCCCCCCCGCCCCCGCCTGCGCCTGTCG
PI 286070       GATCGATTAGGTAGATCGGGCCGTTACACTCCGCCCCCCCGCCCCCGCCTGCGCCTGTCG
PI 94748        GATCGATTAGGTAGATCGGGCCGTTACACTCCGCCCCCCCGCCCCCGCCTGCGCCTGTCG
PS5             GATCGATTAGGTAGATCGGGCCGTTACACTCCGCCCCCCCGCCCCCGCCTGCGCCTGTCG
PI 532501       GATCGATTAGGTAGATCGGGCCGTTACACTCCGCCCCCCCGCCCCCGCCTGCGCCTGTCG
PI 532475       GATCGATTAGGTAGATCGGGCCGTTACACTCCGCCCCCCCGCCCCCGCCTGCGCCTGTCG
PI 470732       GATCGATTAGGTAGATCGGGCCGTTACACTCCGCCCCCCCGCCCCCGCCTGCGCCTGTCG
PI 349040       GATCGATTAGGTAGATCGGGCCGTTACACTCCGCCCCCCCGCCCCCGCCTGCGCCTGTCG
PI 113961       GATCGATTAGGTAGATCGGGCCGTTACACTCCGCCCCCCCGCCCCCGCCTGCGCCTGTCG
CItr 3686       GATCGATTAGGTAGATCGGGCCGTTACACTCCGCCCCCCCGCCCCCGCCTGCGCCTGTCG
PI 585017       GATCGATTAGGTAGATCGGGCCGTTACACTCCGCCCCCCCGCCCCCGCCTGCGCCTGTCG
PI 532486       GATCGATTAGGTAGATCGGGCCGTTACACTCCGCCCCCCCGCCCCCGCCTGCGCCTGTCG
PI 352282       GATCGATTAGGTAGATCGGGCCGTTACACTCCGCCCCCCCGCCCCCGCCTGCGCCTGTCG
PI 352278       GATCGATTAGGTAGATCGGGCCGTTACACTCCGCCCCCCCGCCCCCGCCTGCGCCTGTCG
PI 283889       GATCGATTAGGTAGATCGGGCCGTTACACTCCGCCCCCCCGCCCCCGCCTGCGCCTGTCG
PI 251914       GATCGATTAGGTAGATCGGGCCGTTACACTCCGCCCCCCCGCCCCCGCCTGCGCCTGTCG
PI 115816       GATCGATTAGGTAGATCGGGCCGTTACACTCCGCCCCCCCGCCCCCGCCTGCGCCTGTCG
```

```
PI 61102        GATCGATTAGGTAGATCGGGCCGTTACACTCCGCCCCCCCGCCCCCGCCTGCGCCTGTCG
PI 283887       GATCGATTAGGTAGATCGGGCCGTTACACTCCGCCCCCCCGCCCCCGCCTGCGCCTGTCG
PI 94749        GATCGATTAGGTAGATCGGGCCGTTACACTCCGCCCCCCCGCCCCCGCCTGCGCCTGTCG
PI 115817       GATCGATTAGGTAGATCGGGCCGTTACACTCCGCCCCCCCGCCCCCGCCTGCGCCTGTCG
                ************************************************************

PI 499972       GAGTAGCCGTCGCGGTCTGCCGGTGTTGGAGGCTTGGGGTGTAGGGTTGGCCCCGTTCTC
CItr 7779       GAGTAGCCGTCGCGGTCTGCCGGTGTTGGAGGCTTGGGGTGTAGGGTTGGCCCCGTTCTC
PI 352281       GAGTAGCCGTCGCGGTCTGCCGGTGTTGGAGGCTTGGGGTGTAGGGTTGGCCCCGTTCTC
PI 286070       GAGTAGCCGTCGCGGTCTGCCGGTGTTGGAGGCTTGGGGTGTAGGGTTGGCCCCGTTCTC
PI 94748        GAGTAGCCGTCGCGGTCTGCCGGTGTTGGAGGCTTGGGGTGTAGGGTTGGCCCCGTTCTC
PS5             GAGTAGCCGTCGCGGTCTGCCGGTGTTGGAGGCTTGGGGTGTAGGGTTGGCCCCGTTCTC
PI 532501       GAGTAGCCGTCGCGGTCTGCCGGTGTTGGAGGCTTGGGGTGTAGGGTTGGCCCCGTTCTC
PI 532475       GAGTAGCCGTCGCGGTCTGCCGGTGTTGGAGGCTTGGGGTGTAGGGTTGGCCCCGTTCTC
PI 470732       GAGTAGCCGTCGCGGTCTGCCGGTGTTGGAGGCTTGGGGTGTAGGGTTGGCCCCGTTCTC
PI 349040       GAGTAGCCGTCGCGGTCTGCCGGTGTTGGAGGCTTGGGGTGTAGGGTTGGCCCCGTTCTC
PI 113961       GAGTAGCCGTCGCGGTCTGCCGGTGTTGGAGGCTTGGGGTGTAGGGTTGGCCCCGTTCTC
CItr 3686       GAGTAGCCGTCGCGGTCTGCCGGTGTTGGAGGCTTGGGGTGTAGGGTTGGCCCCGTTCTC
PI 585017       GAGTAGCCGTCGCGGTCTGCCGGTGTTGGAGGCTTGGGGTGTAGGGTTGGCCCCGTTCTC
PI 532486       GAGTAGCCGTCGCGGTCTGCCGGTGTTGGAGGCTTGGGGTGTAGGGTTGGCCCCGTTCTC
PI 352282       GAGTAGCCGTCGCGGTCTGCCGGTGTTGGAGGCTTGGGGTGTAGGGTTGGCCCCGTTCTC
PI 352278       GAGTAGCCGTCGCGGTCTGCCGGTGTTGGAGGCTTGGGGTGTAGGGTTGGCCCCGTTCTC
PI 283889       GAGTAGCCGTCGCGGTCTGCCGGTGTTGGAGGCTTGGGGTGTAGGGTTGGCCCCGTTCTC
PI 251914       GAGTAGCCGTCGCGGTCTGCCGGTGTTGGAGGCTTGGGGTGTAGGGTTGGCCCCGTTCTC
PI 115816       GAGTAGCCGTCGCGGTCTGCCGGTGTTGGAGGCTTGGGGTGTAGGGTTGGCCCCGTTCTC
PI 61102        GAGTAGCCGTCGCGGTCTGCCGGTGTTGGAGGCTTGGGGTGTAGGGTTGGCCCCGTTCTC
PI 283887       GAGTAGCCGTCGCGGTCTGCCGGTGTTGGAGGCTTGGGGTGTAGGGTTGGCCCCGTTCTC
PI 94749        GAGTAGCCGTCGCGGTCTGCCGGTGTTGGAGGCTTGGGGTGTAGGGTTGGCCCCGTTCTC
PI 115817       GAGTAGCCGTCGCGGTCTGCCGGTGTTGGAGGCTTGGGGTGTAGGGTTGGCCCCGTTCTC
                ************************************************************

PI 499972       CAGCGGAGATGGGGC
CItr 7779       CAGCGGAGATGGGGC
PI 352281       CAGCGGAGATGGGGC
PI 286070       CAGCGGAGATGGGGC
PI 94748        CAGCGGAGATGGGGC
PS5             CAGCGGAGATGGGGC
PI 532501       CAGCGGAGATGGGGC
PI 532475       CAGCGGAGATGGGGC
PI 470732       CAGCGGAGATGGGGC
PI 349040       CAGCGGAGATGGGGC
PI 113961       CAGCGGAGATGGGGC
CItr 3686       CAGCGGAGATGGGGC
PI 585017       CAGCGGAGATGGGGC
PI 532486       CAGCGGAGATGGGGC
PI 352282       CAGCGGAGATGGGGC
PI 352278       CAGCGGAGATGGGGC
PI 283889       CAGCGGAGATGGGGC
PI 251914       CAGCGGAGATGGGGC
PI 115816       CAGCGGAGATGGGGC
PI 61102        CAGCGGAGATGGGGC
PI 283887       CAGCGGAGATGGGGC
PI 94749        CAGCGGAGATGGGGC
PI 115817       CAGCGGAGATGGGGC
                ***************
```

Figure S7 Alignment of the sequences contains 5'- and 3'- ends of retrotrans_VRN in PI 94749 and other 22 tetraploid wheat accessions. The two CTCCG motifs are shown in red and highlighted in yellow.
