# Supplementary material for: A Novel Retrotransposon Inserted in the Dominant Vrn-B1 Allele Confers Spring Growth Habit in Tetraploid Wheat (Triticum turgidum L.)
Source: G3 (Bethesda). 2011 Dec 1;1(7):637–45. doi: 10.1534/g3.111.001131 (PMC3276170; doi:10.1534/g3.111.001131)
Supplement: Supporting Information [file supp_1.7.637_TableS1.pdf]

Table S1 Winter/spring growth habit in the 146 double haploid (DH) lines derived from the cross between durum wheat 'Lebsock' and *T. turgidum* subsp. *carthlicum* accession PI 94749 [a]

Spring DH lines with *Vrn-A1* and *Vrn-B1* alleles (31 lines):

LP749-6	LP749-7	LP749-13	LP749-16	LP749-22	LP749-27	LP749-29	LP749-37
LP749-42	LP749-49	LP749-52	LP749-55	LP749-57	LP749-64	LP749-66	LP749-68
LP749-71	LP749-80	LP749-81	LP749-85	LP749-87	LP749-89	LP749-90	LP749-92
LP749-93	LP749-96	LP749-107	LP749-111	LP749-117	LP749-122	LP749-138	

Spring DH lines with *Vrn-A1* and *vrn-B1* alleles (33 lines):

LP749-4	LP749-8	LP749-11	LP749-14	LP749-15	LP749-17	LP749-19	LP749-21
LP749-32	LP749-33	LP749-39	LP749-41	LP749-44	LP749-50	LP749-60	LP749-62
LP749-63	LP749-67	LP749-70	LP749-72	LP749-82	LP749-83	LP749-84	LP749-97
LP749-109	LP749-113	LP749-114	LP749-120	LP749-126	LP749-130	LP749-139	LP749-140
LP749-146							

Spring DH lines with *vrn-A1* and *Vrn-B1* alleles (37 lines):

LP749-2	LP749-5	LP749-10	LP749-18	LP749-20	LP749-23	LP749-35	LP749-40
LP749-43	LP749-47	LP749-54	LP749-56	LP749-58	LP749-59	LP749-65	LP749-73
LP749-74	LP749-75	LP749-76	LP749-78	LP749-86	LP749-88	LP749-91	LP749-102
LP749-103	LP749-104	LP749-108	LP749-110	LP749-116	LP749-118	LP749-123	LP749-124
LP749-125	LP749-133	LP749-137	LP749-141	LP749-145			

Winter DH lines with *vrn-A1* and *vrn-B1* alleles (45 lines):

LP749-1	LP749-3	LP749-9	LP749-12	LP749-24	LP749-25	LP749-26	LP749-28
LP749-30	LP749-31	LP749-34	LP749-36	LP749-38	LP749-45	LP749-46	LP749-48
LP749-51	LP749-53	LP749-61	LP749-69	LP749-77	LP749-79	LP749-94	LP749-95
LP749-98	LP749-99	LP749-100	LP749-101	LP749-105	LP749-106	LP749-112	LP749-115
LP749-119	LP749-121	LP749-127	LP749-128	LP749-129	LP749-131	LP749-132	LP749-134
LP749-135	LP749-136	LP749-142	LP749-143	LP749-144			

[a] *VRN*-1 alleles carried by each line were verified through gene specific marker developed in this study.
