# Supplementary material for: A Novel Retrotransposon Inserted in the Dominant Vrn-B1 Allele Confers Spring Growth Habit in Tetraploid Wheat (Triticum turgidum L.)
Source: G3 (Bethesda). 2011 Dec 1;1(7):637–45. doi: 10.1534/g3.111.001131 (PMC3276170; doi:10.1534/g3.111.001131)
Supplement: Supporting Information [file supp_1.7.637_TableS2.pdf]

Table S2 Frequency of *Vrn-B1* allele containing retrotrans_VRN in 154 spring type accessions or lines from six tetraploid wheat (*Triticum turgidum* L.) sub-species.

Accession no. or line [a]	Origin [b]	Sub-species	PCR amplification [c] VRNBPF1/VRNBPR1	PCR amplification [c] VRNBPF1/INSR1	Containing retrotrans_VRN [d]
CItr 7665	Russian Federation	*T. turgidum* subsp. *carthlicum*	1	0	-
PI 182471	Turkey	*T. turgidum* subsp. *carthlicum*	1	0	-
PI 341800	Russian Federation	*T. turgidum* subsp. *carthlicum*	1	0	-
PI 61102	Georgia	*T. turgidum* subsp. *carthlicum*	0	1	+
PI 94748	Georgia	*T. turgidum* subsp. *carthlicum*	0	1	+
PI 115816	Georgia	*T. turgidum* subsp. *carthlicum*	0	1	+
PI 115817	Georgia	*T. turgidum* subsp. *carthlicum*	0	1	+
PI 251914	Georgia	*T. turgidum* subsp. *carthlicum*	0	1	+
PI 283887	Iran	*T. turgidum* subsp. *carthlicum*	0	1	+
PI 283889	Iran	*T. turgidum* subsp. *carthlicum*	0	1	+
PI 286070	Poland	*T. turgidum* subsp. *carthlicum*	0	1	+
PI 349040	Armenia	*T. turgidum* subsp. *carthlicum*	0	1	+
PI 352278	Georgia	*T. turgidum* subsp. *carthlicum*	0	1	+
PI 352281	Caucasus region in Former Soviet Union	*T. turgidum* subsp. *carthlicum*	0	1	+
PI 352282	Georgia	*T. turgidum* subsp. *carthlicum*	0	1	+
PI 470732	Turkey	*T. turgidum* subsp. *carthlicum*	0	1	+
PI 499972	Georgia	*T. turgidum* subsp. *carthlicum*	0	1	+
PI 532475	Turkey	*T. turgidum* subsp. *carthlicum*	0	1	+
PI 532486	Turkey	*T. turgidum* subsp. *carthlicum*	0	1	+
PI 532501	Former Soviet Union	*T. turgidum* subsp. *carthlicum*	0	1	+
PI 585017	Georgia	*T. turgidum* subsp. *carthlicum*	0	1	+
PS5	China	*T. turgidum* subsp. *carthlicum*	0	1	+
CItr 3686	MN, USA	*T. turgidum* subsp. *dicoccum*	0	1	+
CItr 7779	Ethiopia	*T. turgidum* subsp. *dicoccum*	0	1	+
PI 113961	Georgia	*T. turgidum* subsp. *dicoccum*	0	1	+
CItr 14133	AZ, USA	*T. turgidum* subsp. *dicoccum*	1	0	-
CItr 4013	India	*T. turgidum* subsp. *dicoccum*	1	0	-
CItr 7685	Russian Federation	*T. turgidum* subsp. *dicoccum*	1	0	-
CItr 7962	Ethiopia	*T. turgidum* subsp. *dicoccum*	1	0	-
CItr 12214	India	*T. turgidum* subsp. *dicoccum*	1	0	-
CItr 14098	Ethiopia	*T. turgidum* subsp. *dicoccum*	1	0	-
CItr 14454	Ethiopia	*T. turgidum* subsp. *dicoccum*	1	0	-

CItr 14592	Ethiopia	*T. turgidum* subsp. *dicoccum*	1	0	-
CItr 14621	Ethiopia	*T. turgidum* subsp. *dicoccum*	1	0	-
CItr 14636	Ethiopia	*T. turgidum* subsp. *dicoccum*	1	0	-
CItr 14751	Ethiopia	*T. turgidum* subsp. *dicoccum*	1	0	-
CItr 14822	Eritrea	*T. turgidum* subsp. *dicoccum*	1	0	-
CItr 14868	Ethiopia	*T. turgidum* subsp. *dicoccum*	1	0	-
CItr 14919	Unknown	*T. turgidum* subsp. *dicoccum*	1	0	-
CItr 14970	Unknown	*T. turgidum* subsp. *dicoccum*	1	0	-
PI 101971	India	*T. turgidum* subsp. *dicoccum*	1	0	-
PI 133134	Peru	*T. turgidum* subsp. *dicoccum*	1	0	-
PI 154582	China	*T. turgidum* subsp. *dicoccum*	1	0	-
PI 164578	India	*T. turgidum* subsp. *dicoccum*	1	0	-
PI 168673	WI, USA	*T. turgidum* subsp. *dicoccum*	1	0	-
PI 190926	Belgium	*T. turgidum* subsp. *dicoccum*	1	0	-
PI 191091	Spain	*T. turgidum* subsp. *dicoccum*	1	0	-
PI 191387	Ethiopia	*T. turgidum* subsp. *dicoccum*	1	0	-
PI 193641	Ethiopia	*T. turgidum* subsp. *dicoccum*	1	0	-
PI 193873	Ethiopia	*T. turgidum* subsp. *dicoccum*	1	0	-
PI 193878	Ethiopia	*T. turgidum* subsp. *dicoccum*	1	0	-
PI 194041	Ethiopia	*T. turgidum* subsp. *dicoccum*	1	0	-
Alkabo	ND, USA	*T. turgidum* subsp. *durum*	1	0	-
Ben	ND, USA	*T. turgidum* subsp. *durum*	1	0	-
Golden Ball	South Africa	*T. turgidum* subsp. *durum*	1	0	-
Mountrail	ND, USA	*T. turgidum* subsp. *durum*	1	0	-
Nora	ND, USA	*T. turgidum* subsp. *durum*	1	0	-
Parshall	ND, USA	*T. turgidum* subsp. *durum*	1	0	-
Renville	ND, USA	*T. turgidum* subsp. *durum*	1	0	-
Rugby	ND, USA	*T. turgidum* subsp. *durum*	1	0	-
Wells	ND, USA	*T. turgidum* subsp. *durum*	1	0	-
TA 4154-1	CIMMYT	*T. turgidum* subsp. *durum*	1	0	-
TA 4154-2	CIMMYT	*T. turgidum* subsp. *durum*	1	0	-
TA 4154-3	CIMMYT	*T. turgidum* subsp. *durum*	1	0	-
TA 4154-4	CIMMYT	*T. turgidum* subsp. *durum*	1	0	-
TA 4154-5	CIMMYT	*T. turgidum* subsp. *durum*	1	0	-
TA 4154-6	CIMMYT	*T. turgidum* subsp. *durum*	1	0	-
TA 4154-7	CIMMYT	*T. turgidum* subsp. *durum*	1	0	-
TA 4154-8	CIMMYT	*T. turgidum* subsp. *durum*	1	0	-
TA 4154-9	CIMMYT	*T. turgidum* subsp. *durum*	1	0	-
TA 4154-10	CIMMYT	*T. turgidum* subsp. *durum*	1	0	-

TA 4154-11	CIMMYT	*T. turgidum* subsp. *durum*	1	0	-
TA 4154-12	CIMMYT	*T. turgidum* subsp. *durum*	1	0	-
TA 4154-13	CIMMYT	*T. turgidum* subsp. *durum*	1	0	-
TA 4154-14	CIMMYT	*T. turgidum* subsp. *durum*	1	0	-
TA 4154-15	CIMMYT	*T. turgidum* subsp. *durum*	1	0	-
TA 4154-16	CIMMYT	*T. turgidum* subsp. *durum*	1	0	-
TA 4154-17	CIMMYT	*T. turgidum* subsp. *durum*	1	0	-
TA 4154-18	CIMMYT	*T. turgidum* subsp. *durum*	1	0	-
TA 4154-19	CIMMYT	*T. turgidum* subsp. *durum*	1	0	-
TA 4154-20	CIMMYT	*T. turgidum* subsp. *durum*	1	0	-
TA 4154-21	CIMMYT	*T. turgidum* subsp. *durum*	1	0	-
TA 4154-22	CIMMYT	*T. turgidum* subsp. *durum*	1	0	-
TA 4154-23	CIMMYT	*T. turgidum* subsp. *durum*	1	0	-
TA 4154-25	CIMMYT	*T. turgidum* subsp. *durum*	1	0	-
PI 42209	Australia	*T. turgidum* subsp. *polonicum*	1	0	-
PI 56261	Portugal	*T. turgidum* subsp. *polonicum*	1	0	-
PI 56262	Portugal	*T. turgidum* subsp. *polonicum*	1	0	-
PI 167622	Turkey	*T. turgidum* subsp. *polonicum*	1	0	-
PI 190951	Portugal	*T. turgidum* subsp. *polonicum*	1	0	-
PI 191620	Portugal	*T. turgidum* subsp. *polonicum*	1	0	-
CItr 14139	Unknown	*T. turgidum* subsp. *polonicum*	1	0	-
CItr 17442	CA, USA	*T. turgidum* subsp. *polonicum*	1	0	-
PI 192666	Portugal	*T. turgidum* subsp. *polonicum*	1	0	-
PI 208911	Iraq	*T. turgidum* subsp. *polonicum*	1	0	-
PI 210845	Iran	*T. turgidum* subsp. *polonicum*	1	0	-
PI 223171	Jordan	*T. turgidum* subsp. *polonicum*	1	0	-
PI 266846	England, UK	*T. turgidum* subsp. *polonicum*	1	0	-
PI 272564	Hungary	*T. turgidum* subsp. *polonicum*	1	0	-
PI 272570	Hungary	*T. turgidum* subsp. *polonicum*	1	0	-
PI 286547	Ecuador	*T. turgidum* subsp. *polonicum*	1	0	-
PI 290512	Portugal	*T. turgidum* subsp. *polonicum*	1	0	-
PI 306548	Romania	*T. turgidum* subsp. *polonicum*	1	0	-
PI 330554	England, UK	*T. turgidum* subsp. *polonicum*	1	0	-
PI 330555	England, UK	*T. turgidum* subsp. *polonicum*	1	0	-
CItr 7809	Ethiopia	*T. turgidum* subsp. *turgidum*	1	0	-
CItr 7839	Ethiopia	*T. turgidum* subsp. *turgidum*	1	0	-
CItr 7859	Ethiopia	*T. turgidum* subsp. *turgidum*	1	0	-
CItr 13712	OR, USA	*T. turgidum* subsp. *turgidum*	1	0	-
PI 32039	China	*T. turgidum* subsp. *turgidum*	1	0	-

PI 41029	Georgia	*T. turgidum* subsp. *turgidum*	1	0	-
PI 60617	Ethiopia	*T. turgidum* subsp. *turgidum*	1	0	-
PI 60729	Egypt	*T. turgidum* subsp. *turgidum*	1	0	-
PI 67339	Australia	*T. turgidum* subsp. *turgidum*	1	0	-
PI 94689	Armenia	*T. turgidum* subsp. *turgidum*	1	0	-
PI 134947	Portugal	*T. turgidum* subsp. *turgidum*	1	0	-
PI 134948	Portugal	*T. turgidum* subsp. *turgidum*	1	0	-
PI 134951	Portugal	*T. turgidum* subsp. *turgidum*	1	0	-
PI 134953	Portugal	*T. turgidum* subsp. *turgidum*	1	0	-
PI 134954	Portugal	*T. turgidum* subsp. *turgidum*	1	0	-
PI 134956	Portugal	*T. turgidum* subsp. *turgidum*	1	0	-
PI 134957	Portugal	*T. turgidum* subsp. *turgidum*	1	0	-
PI 134959	Portugal	*T. turgidum* subsp. *turgidum*	1	0	-
PI 134962	Portugal	*T. turgidum* subsp. *turgidum*	1	0	-
PI 254206	Iran	*T. turgidum* subsp. *turanicum*	1	0	-
PI 254208	Iran	*T. turgidum* subsp. *turanicum*	1	0	-
PI 254212	Iran	*T. turgidum* subsp. *turanicum*	1	0	-
PI 254213	Turkey	*T. turgidum* subsp. *turanicum*	1	0	-
PI 256034	Spain	*T. turgidum* subsp. *turanicum*	1	0	-
PI 272601	Hungary	*T. turgidum* subsp. *turanicum*	1	0	-
PI 272602	Hungary	*T. turgidum* subsp. *turanicum*	1	0	-
PI 278350	Italy	*T. turgidum* subsp. *turanicum*	1	0	-
PI 283795	Afghanistan	*T. turgidum* subsp. *turanicum*	1	0	-
PI 286069	Poland	*T. turgidum* subsp. *turanicum*	1	0	-
PI 290530	Hungary	*T. turgidum* subsp. *turanicum*	1	0	-
PI 306665	France	*T. turgidum* subsp. *turanicum*	1	0	-
PI 317495	Afghanistan	*T. turgidum* subsp. *turanicum*	1	0	-
PI 321737	Afghanistan	*T. turgidum* subsp. *turanicum*	1	0	-
PI 337643	Afghanistan	*T. turgidum* subsp. *turanicum*	1	0	-
PI 347132	Afghanistan	*T. turgidum* subsp. *turanicum*	1	0	-
PI 349055	Russian Federation	*T. turgidum* subsp. *turanicum*	1	0	-
PI 352514	Azerbaijan	*T. turgidum* subsp. *turanicum*	1	0	-
PI 352515	Iran	*T. turgidum* subsp. *turanicum*	1	0	-
PI 362067	Romania	*T. turgidum* subsp. *turanicum*	1	0	-
PI 481582	Iraq	*T. turgidum* subsp. *turanicum*	1	0	-
PI 532136	Egypt	*T. turgidum* subsp. *turanicum*	1	0	-
PI 537992	Yemen	*T. turgidum* subsp. *turanicum*	1	0	-
PI 559976	Morocco	*T. turgidum* subsp. *turanicum*	1	0	-
PI 560896	Turkey	*T. turgidum* subsp. *turanicum*	1	0	-

PI 623629	Iran	*T. turgidum* subsp. *turanicum*	1	0	-
PI 624208	Iran	*T. turgidum* subsp. *turanicum*	1	0	-
PI 624217	Iran	*T. turgidum* subsp. *turanicum*	1	0	-
PI 624421	Iran	*T. turgidum* subsp. *turanicum*	1	0	-
PI 624422	Iran	*T. turgidum* subsp. *turanicum*	1	0	-

[a] TA and PI/CItr are the accession numbers in Wheat Genetic and Genomic Resource Center (WGGRC) at Kansas State University in Manhattan, Kansas and USDA National Small Grains Collection, Aberdeen, Idaho, respectively.

[b] Origin of the accessions was obtained through USDA National Plant Germplasm System (NPGS) (http://www.ars-grin.gov/npgs/). Durum lines with TA number were originally obtained from International Maize and Wheat Improvement Center (CIMMYT), Mexico.

[c] Two primer pairs VRNBPF1/ VRNBPR1 and VRNBPF1/ INSR1 can detect the absence and presence of retrotrans_VRN in *VRN-B1*, respectively. For PCR amplification, 1 and 0 means the presence and absence of the specific band, respectively.

[d] '+' and '-' represents presence and absence, respectively.
