# Supplementary material for: A Novel Retrotransposon Inserted in the Dominant Vrn-B1 Allele Confers Spring Growth Habit in Tetraploid Wheat (Triticum turgidum L.)
Source: G3 (Bethesda). 2011 Dec 1;1(7):637–45. doi: 10.1534/g3.111.001131 (PMC3276170; doi:10.1534/g3.111.001131)
Supplement: Supporting Information [file supp_1.7.637_TableS3.pdf]

Table S3 The growth habit and days to heading of the DH lines that were used in the expression experiment for *VRN-1* genes.

Line	Genotype	Growth habit	No. of plants used		Days to heading (days)	
			Unvernalized	vernalized	Unvernalized	Vernalized
Lebsock	*Vrn-A1Vrn-A1 vrn-B1vrn-B1*	Spring	5	-	51.6	-
PI 94749	*vrn-A1vrn-A1 Vrn-B1Vrn-B1*	Spring	5	-	67	-
LP749-7	*Vrn-A1Vrn-A1 Vrn-B1Vrn-B1*	Spring	5	-	54	-
LP749-16	*Vrn-A1Vrn-A1 Vrn-B1Vrn-B1*	Spring	5	-	47	-
LP749-71	*Vrn-A1Vrn-A1 Vrn-B1Vrn-B1*	Spring	4	-	50	-
LP749-80	*Vrn-A1Vrn-A1 Vrn-B1Vrn-B1*	Spring	5	-	49.2	-
LP749-8	*Vrn-A1Vrn-A1 vrn-B1vrn-B1*	Spring	5	-	54.4	-
LP749-11	*Vrn-A1Vrn-A1 vrn-B1vrn-B1*	Spring	4	-	52.5	-
LP749-82	*Vrn-A1Vrn-A1 vrn-B1vrn-B1*	Spring	5	-	56	-
LP749-97	*Vrn-A1Vrn-A1 vrn-B1vrn-B1*	Spring	5	-	56.4	-
LP749-10	*vrn-A1vrn-A1 Vrn-B1Vrn-B1*	Spring	4	-	50.3	-
LP749-18	*vrn-A1vrn-A1 Vrn-B1Vrn-B1*	Spring	5	-	55.4	-
LP749-20	*vrn-A1vrn-A1 Vrn-B1Vrn-B1*	Spring	4	-	52.3	-
LP749-43	*vrn-A1vrn-A1 Vrn-B1Vrn-B1*	Spring	5	-	56.6	-
LP749-54	*vrn-A1vrn-A1 Vrn-B1Vrn-B1*	Spring	5	-	58.8	-
LP749-58	*vrn-A1vrn-A1 Vrn-B1Vrn-B1*	Spring	5	-	55.4	-
LP749-65	*vrn-A1vrn-A1 Vrn-B1Vrn-B1*	Spring	4	-	47.8	-

LP749-88	*vrn-A1vrn-A1 Vrn-B1Vrn-B1*	Spring	5	-	49.4	-
LP749-25	*vrn-A1vrn-A1 vrn-B1vrn-B1*	Winter	5	-	92.6	-
LP749-28	*vrn-A1vrn-A1 vrn-B1vrn-B1*	Winter	5	-	105	-
LP749-30	*vrn-A1vrn-A1 vrn-B1vrn-B1*	Winter	5	-	145	-
LP749-34	*vrn-A1vrn-A1 vrn-B1vrn-B1*	Winter	3	-	97	-
LP749-53	*vrn-A1vrn-A1 vrn-B1vrn-B1*	Winter	4	-	111.3	-
LP749-36	*vrn-A1vrn-A1 vrn-B1vrn-B1*	Winter	5	5	145	112.8
LP749-61	*vrn-A1vrn-A1 vrn-B1vrn-B1*	Winter	5	5	145	106.8
